# Supplementary figures and images for: Lasiodiplodia mitidjana sp. nov. and other Botryosphaeriaceae species causing branch canker and dieback of Citrus sinensis in Algeria
Source: PLoS One. 2020 May 20;15(5):e0232448. doi: 10.1371/journal.pone.0232448 (PMC7239386; doi:10.1371/journal.pone.0232448)

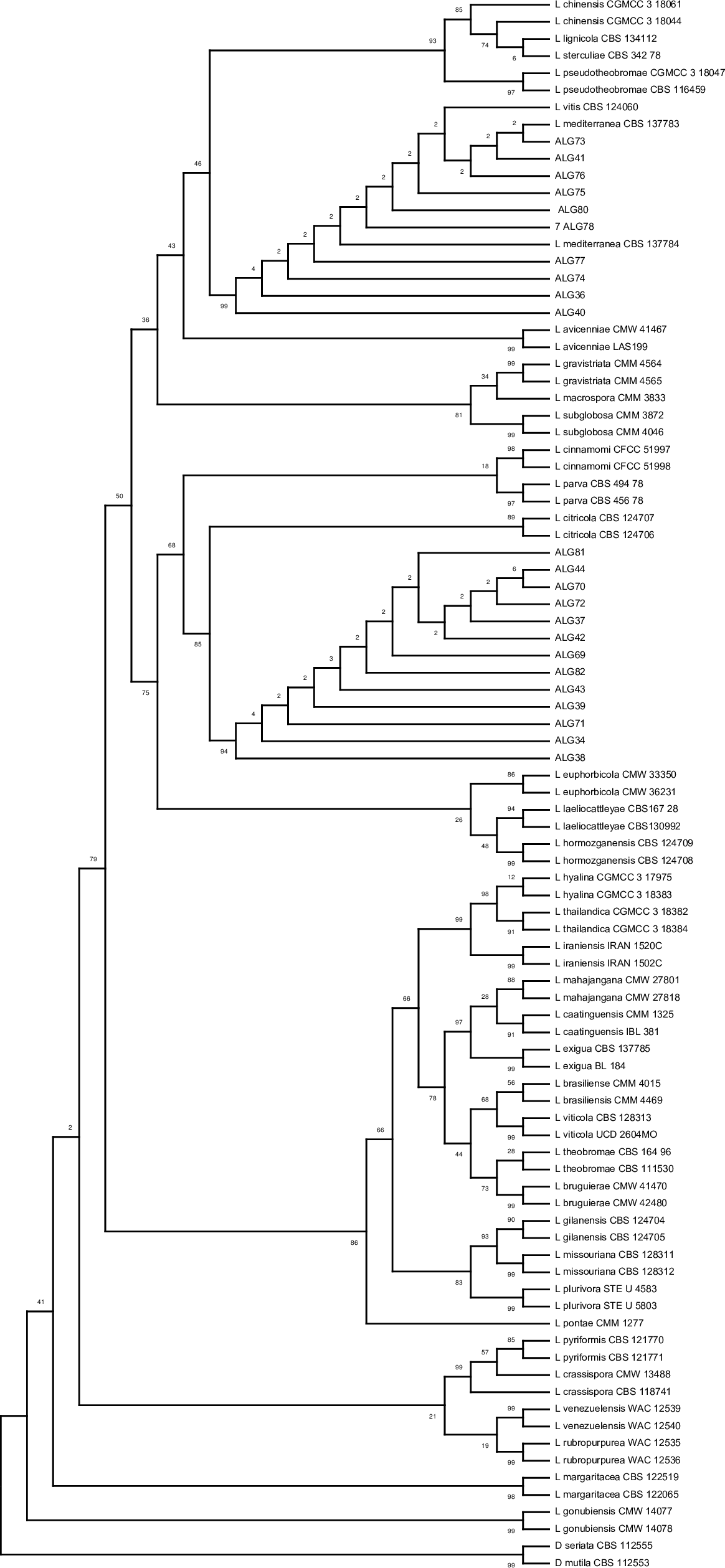

Supplement: S1 Fig — The tree was rooted to Diplodia mutila and Diplodia seriata. (TIF) [file pone.0232448.s001.tif]
