# Supplementary material for: Lasiodiplodia mitidjana sp. nov. and other Botryosphaeriaceae species causing branch canker and dieback of Citrus sinensis in Algeria
Source: PLoS One. 2020 May 20;15(5):e0232448. doi: 10.1371/journal.pone.0232448 (PMC7239386; doi:10.1371/journal.pone.0232448)
Supplement: S1 Table — (DOCX) [file pone.0232448.s002.docx]

**Supplementary table S1.** Details of strains included in the phylogenetic and/or morphological analyses.

| **Species** | **Culture collection number(s)^1^** | **Substrate** | **Country** | **Collector(s)** | **GenBank accession numbers** | |
| --- | --- | --- | --- | --- | --- | --- |
|  |  |  |  |  | **ITS** | ***tef1*** |
| *L. avicenniae* | CMW 41467 | *Avicennia,* asymptomatic branches | South Africa | J. A. Osorio & J. Roux | KP860835 | KP860680 |
| *L. avicenniae* | LAS199 | *Avicennia,* asymptomatic branches | South Africa | J. A. Osorio & J. Roux | KU587957 | KU587947 |
| *L. brasiliense* | CMM 4015, ex-type | *Mangifera*, stems | Brazil | M. W. Marques | JX464063 | JX464049 |
| *L. brasiliense* | CMM 4469 | *Anacardium* | Brazil | - | KT325574 | KT325580 |
| *L. bruguierae* | CMW 41470 | *Bruguiera¸* asymptomatic branches | South Africa | J. A. Osorio & J. Roux | KP860833 | KP860678 |
| *L. bruguierae* | CMW 42480 | *Bruguiera,* asymptomatic branches | South Africa | J. A. Osorio & J. Roux | KP860832 | KP860677 |
| *L. caatinguensis* | CMM 1325 | *Citrus* | Brazil | I. B. L. Coutinho & J. S. Lima | KT154760 | KT008006 |
| *L. caatinguensis* | IBL 381 | *Spondias* | Brazil | J. S. Lima & J. E. Cardoso | KT154757 | KT154751 |
| *L. chinensis* | CGMCC 3.18061 | *Unknown*, branch | China | W. He & Z. P. Dou | KX499889 | KX499927 |
| *L. chinensis* | CGMCC 3.18044 | *Vaccinium,* branch | China | J. H. Zhao | KX499875 | KX499913 |
| *L. cinnamomi* | CFCC 51997 | *Cinnamomum,* branch | China | N. Jiang | MG866028 | MH236799 |
| *L. cinnamomi* | CFCC 51998 | *Cinnamomum,* branch | China | N. Jiang | MG866029 | MH236800 |
| *L. citricola* | CBS 124706 | *Citrus* sp., twigs | Iran | A. Shekari | GU945353 | GU945339 |
| *L. citricola* | CBS 124707, ex-type | *Citrus*, twigs | Iran | J. Abdollahzadeh & A. Javadi | GU945354 | GU945340 |
| *L. crassispora* | CMW 13488 | *Eucalyptus,* wood | Venezuela | S. Mohali | DQ103552 | DQ103559 |
| *L. crassispora* | CBS 118741, ex-type | *Santalum* | Australia | T. I. Burgess & B. Dell | DQ103550 | EU673303 |
| *L. euphorbicola* | CMW 33350 | *Adansonia* | Botswana | - | KU887149 | KU887026 |
| *L. euphorbicola* | CMW 36231 | *Adansonia* | Zimbabwe | - | KU887187 | KU887063 |
| *L. exigua* | BL 184 | *Retama*, branch canker | Tunisia | B. T. Linaldeddu | KJ638318 | KJ638337 |
| *L. exigua* | CBS 137785, ex-type | *Retama*, branch canker | Tunisia | B. T. Linaldeddu | KJ638317 | KJ638336 |
| *L. gilanensis* | CBS 124704, ex-type | *Citrus*, fallen twigs | Iran | J. Abdollahzadeh & A. Javadi | GU945351 | GU945342 |
| *L. gilanensis* | CBS 124705 | *Citrus* sp., fallen twigs | Iran | J. Abdollahzadeh & A. Javadi | GU945352 | GU945341 |
| *L. gonubiensis* | CMW 14077, ex-type | *Syzygium* | South Africa | D. Pavlic | AY639595 | DQ103566 |
| *L. gonubiensis* | CMW 14078, ex-paratype | *Syzigium* | South Africa | D. Pavlic | AY639594 | DQ103567 |
| *L. gravistriata* | CMM 4564 | *Anacardium,* stems | Brazil | M. S. B. Netto | KT250949 | KT250950 |
| *L. gravistriata* | CMM 4565 | *Anacardium,* stems | Brazil | M. S. B. Netto | KT250947 | KT266812 |
| *L. hormozganensis* | CBS 124708 | *Mangifera*, twigs | Iran | J. Abdollahzadeh & A. Javadi | GU945356 | GU945344 |
| *L. hormozganensis* | CBS 124709, ex-type | *Olea*, twigs | Iran | J. Abdollahzadeh & A. Javadi | GU945355 | GU945343 |
| *L. hyalina* | CGMCC 3.17975 | *Acacia,* cankered stems | China | Y. Zhang & Y. P. Zhou | KX499879 | KX499917 |
| *L. hyalina* | CGMCC 3.18383 | unidentified woody plant, cankered branches | China | Z. P. Dou & Z. C. Liu | KY767661 | KY751302 |
| *L. iraniensis* | IRAN 1520C, ex-type | *Salvadora*, twigs | Iran | J. Abdollahzadeh & A. Javadi | GU945346 | GU945334 |
| *L. iraniensis* | IRAN 1502C | *Juglans*, twigs | Iran | A. Javadi | GU945347 | GU945335 |
| *L. laeliocattleyae* | CBS 167.28 | *Laeliocattleya*, leaves | Italy | C. Sibilia | KU507487 | KU507454 |
| *L. laeliocattleyae* | CBS 130992 | *Mangifera,* leaves | Egypt | A. M. Ismail | JN814397 | JN814424 |
| *L. lignicola* | CBS 134112 | Wood | Thailand | - | JX646797 | KU887003 |
| *L. macrospora* | CMM 3833, ex-type | *Jatropha*, collar and root rot | Brazil | A. R. Machado & O. L. Pereira | KF234557 | KF226718 |
| *L. mahajangana* | CMW 27801, ex-type | *Terminalia*, healthy branches | Madagascar | J. Roux | FJ900595 | FJ900641 |
| *L. mahajangana* | CMW 27820 | *Terminalia*, healthy branches | Madagascar | J. Roux | FJ900597 | FJ900643 |
| *L. margaritacea* | CBS 122519, ex-type | *Adansonia,* dying twigs | Australia | T. I. Burgess & M. J. Wingfield | EU144050 | EU144065 |
| *L. margaritacea* | CBS 122065 | *Adansonia gibbosa,* dying twigs | Australia | T. I. Burgess | EU144051 | EU144066 |
| *L. mediterranea* | CBS 137783, ex-type | *Quercus*, branch canker | Italy | B. T. Linaldeddu | KJ638312 | KJ638331 |
| *L. mediterranea* | CBS 137784 | *Vitis*, brown stripe under the bark | Italy | S. Serra | KJ170150 | KJ170151 |
| *L. missouriana* | CBS 128311, ex-type | Wedge-shape canker of grapevine cv. Catawba (complex hybrid of North America *Vitis* species) | USA | K. Striegler & G. M. Leavitt | HQ288225 | HQ288267 |
| *L. missouriana* | CBS 128312 | Wedge-shape canker of grapevine cv. Catawba (complex hybrid of North America *Vitis* species) | USA | K. Striegler & G. M. Leavitt | HQ288226 | HQ288268 |
| *L. parva* | CBS 456.78, ex-type | Cassava-field soil | Colombia | O. Rangel | EF622083 | EF622063 |
| *L. parva* | CBS 494.78 | Cassava-field soil | Colombia | O. Rangel | EF622084 | EF622064 |
| *L. plurivora* | STE-U 5803, ex-type | *Prunus*, wood canker | South Africa | U. Damm | EF445362 | EF445395 |
| *L. plurivora* | STE-U 4583 | *Vitis,* symptomatic | South Africa | F. Halleen | AY343482 | EF445396 |
| *L. pontae* | CMM 1277 | *Spondias,* necrotic canker | Brazil | J.S. Lima & F.C.O. Freire | KT151794 | KT151791 |
| *L. pseudotheobromae* | CBS 116459, ex-type | *Gmelina*, twigs | Costa Rica | J. Carranza- Velazquez | EF622077 | EF622057 |
| *L. pseudotheobromae* | CGMCC 3.18047 | *Pteridium,* twigs | China | - | KX499876 | KX499914 |
| *L. pyriformis* | CBS 121770, ex-type | *Pinus*, fruiting structures | Namibia | F. J. J. van der Walt & J. Roux | EU101307 | EU101352 |
| *L. pyriformis* | CBS 121771 | *Pinus*, fruiting structures | Namibia | F. J. J. van der Walt & J. Roux | EU101308 | EU101353 |
| *L. rubropurpurea* | WAC 12535, ex-type | *Eucalyptus*, canker | Australia | T. I. Burgess | DQ103553 | EU673304 |
| *L. rubropurpurea* | WAC 12536 | *Eucalyptus*, canker | Australia | T. I. Burgess | DQ103554 | DQ103572 |
| *L. sterculiae* | CBS 342.78, ex-type | *Sterculia* | Germany | S. Bruhn | KX464140 | KX464634 |
| *L. subglobosa* | CMM 3872, ex-type | *Jatropha*, collar and root rot | Brazil | A. R. Machado & O. L. Pereira | KF234558 | KF226721 |
| *L. subglobosa* | CMM 4046 | *Jatropha* | Brazil | A. R. Machado & O. L. Pereira | KF234560 | KF226723 |
| *L. thailandica* | CGMCC 3.18382 | *Podocarpus,* cankered branch | China | D. Zhipeng & L. Zuchen | KY767662 | KY751303 |
| *L. thailandica* | CGMCC 3.18384 | *Albizia,* cankered branch | China | Z.P. Dou & Z.C. Liu | KY767663 | KY751304 |
| *L. theobromae* | CBS 164.96, ex-neotype | Fruit along coral reef coast | Papua New Guinea | A. Aptroot | AY640255 | AY640258 |
| *L. theobromae* | CBS 111530 | *Leucospermum* | USA | J. E. Taylor | EF622074 | EF622054 |
| *L. venezuelensis* | WAC 12539, ex-type | *Acacia*, wood | Venezuela | S. Mohali | DQ103547 | EU673305 |
| *L. venezuelensis* | WAC 12540 | *Acacia*, wood | Venezuela | S. Mohali | DQ103548 | DQ103569 |
| *L. viticola* | CBS 128313, ex-type | Wedge-shape canker of grapevine cv. Vignoles (complex hybrid of North America *Vitis* species) | USA | R. D. Cartwright & W. D. Gubler | HQ288227 | HQ288269 |
| *L. viticola* | UCD 2604MO | *Vitis* | USA | J. R. Urbez-Torres | HQ288228 | HQ288270 |
| *L. vitis* | CBS 124060, ex-type | *Vitis*, wood fragment | Italy, Sicily | S. Burruano | KX464148 | KX464642 |
| *D seriata* | CBS 112555 | *Vitis*, dead stems | Portugal | A. J. L. Phillips | AY259094 | AY573220 |
| *D mutila* | CBS 112553 | *Vitis* | Portugal | A. Alves | AY259093 | AY573219 |

^1^ BL: Personal number of B.T. Linaldeddu; CBS: CBS-KNAW Fungal Biodiversity Centre, Utrecht, The Netherlands; CFCC: China Forestry Culture Collection Center, Beijing, China; CMM: Culture Collection of Phytopathogenic Fungi “Prof. Maria Menezes”, Universidade Federal Rural de Pernambuco, Recife, Brazil; CMW: Tree Patholgy Co-operative Program, Forestry and Agricultural Biotechnology Institute, University of Pretoria, South Africa; IRAN: Iranian Fungal Culture Collection, Iranian Research Institute of Plant Protection, Iran; WAC: Department of Agriculture, Western Australia Plant Pathogen Collection, South Perth, Western Australia.
