# Supplementary material for: Lasiodiplodia mitidjana sp. nov. and other Botryosphaeriaceae species causing branch canker and dieback of Citrus sinensis in Algeria
Source: PLoS One. 2020 May 20;15(5):e0232448. doi: 10.1371/journal.pone.0232448 (PMC7239386; doi:10.1371/journal.pone.0232448)
Supplement: S2 Table — (DOCX) [file pone.0232448.s003.docx]

**Supplementary table S2.** Distribution of the Botryosphaeriaceae species among the surveyed orchards

|  | **Region** | | | | | | | | | |  |
| --- | --- | --- | --- | --- | --- | --- | --- | --- | --- | --- | --- |
|  | **Oued El Alleug** | | | | **Chiffa** | | **Boufarik** | | **Staoueli** | |  |
| **Species/ Orchards** | **1** | **2** | **3** | **4** | **5** | **6** | **7** | **8** | **9** | **10** | **Total** |
| ***D. seriata*** | - | - | - | - | 2 | 1 | - | - | 3 | 4 | 10 |
| ***D. mutila*** | - | - | - | - | 3 | 2 | - | - | - | - | 5 |
| ***L. mediterr.*** | 2 | 2 | 3 | 1 | - | - | 2 | 2 | - | - | 12 |
| ***L. mitidjana*** | 0 | 3 | 3 | 3 | - | - | 2 | 2 | - | - | 13 |
| ***Doth. vitic.*** | 1 | - | 1 | - | 1 | 2 | - | - | 1 | 1 | 7 |
| **Total** | 3 | 5 | 7 | 4 | 6 | 5 | 4 | 4 | 4 | 5 | 47 |
