# Supplementary material for: Lasiodiplodia mitidjana sp. nov. and other Botryosphaeriaceae species causing branch canker and dieback of Citrus sinensis in Algeria
Source: PLoS One. 2020 May 20;15(5):e0232448. doi: 10.1371/journal.pone.0232448 (PMC7239386; doi:10.1371/journal.pone.0232448)
Supplement: Attachment — Submitted filename: PONE-D-19-29810_R1_reviewer.pdf [file pone.0232448.s005.pdf]

PLOS ONE

Lasiodiplodia mitidjana sp. nov. and other Botryosphaeriaceae species causing branch canker and dieback of Citrus sinensis in Algeria

--Manuscript Draft--

**Manuscript Number:**	PONE-D-19-29810R1
**Article Type:**	Research Article
**Full Title:**	Lasiodiplodia mitidjana sp. nov. and other Botryosphaeriaceae species causing branch canker and dieback of Citrus sinensis in Algeria
**Short Title:**	Botrryosphaeriaceae causing dieback on citrus.
**Corresponding Author:**	AKILA BERRAF-TEBBAL<br>Mendel University<br>Lednice, Brno, CZECH REPUBLIC
**Keywords:**	Citrus cultivation, trunk diseases, fungi, identification, taxonomy, pathogenicity
**Abstract:**	Several Botryosphaeriaceae species are known to occur worldwide, causing dieback, canker and fruit rot on various hosts. Surveys conducted in ten commercial citrus orchards in the northern region of Algeria revealed five species in Botryosphaeriaceae belonging to three genera associated with diseased trees. Morphological and cultural characteristics as well as phylogenetic analyses of the internal transcribed spacer (ITS) region and the translation elongation factor 1-alpha (tef1 -α) identified Diplodia mutila , Diplodia seriata , Dothiorella viticola, Lasiodiplodia mediterranea and Lasiodiplodia mithidjana , which is described in this paper as a new species. Of these, L. mithidjana (14.1%) and L. mediterranea (13%) were the most widespread and abundant species. Pathogenicity tests revealed that L. mediterranea and D. seriata were the most aggressive species on citrus shoots. This study highlights the importance of Botryosphaeriaceae species as agents of canker and dieback of citrus trees in Algeria.
**Order of Authors:**	AKILA BERRAF-TEBBAL
	ALLA EDDINE MAHAMEDI
	Wassila Aigoun-Mouhous
	MILAN Špetík
	Jana Čechová
	Robert Pokluda
	Miroslav Baránek
	Aleš Eichmeier
	Artur Alves
**Response to Reviewers:**	Answers to the reviewer 1<br>General comments:<br>Akila Berraf-Tebbal and collaborators present in this manuscript the results of a survey on Botryosphaeriaceae diversity and pathogenicity affecting symptomatic sweet orange (Citrus sinensis) in Algeria. Botryosphaeriacea species were identified in every orchard tested (n=10) and a total of five different species have been isolated from symptomatic samples, with frequencies ranging from 5.4% to 14.1% of the samples. One species appears to form a distinct monophyletic group - not described before - and is claimed by the authors to be a new species. The pathogenicity of two representative isolates for every species isolated (n=5) was tested experimentally on Citrus shoot and Koch's postulate was verified for all of them. Differences in pathogenicity was observed between isolates.<br>Overall, the manuscript is well written and there is no doubt about the efforts made by the authors to produce this work. The results presented are interesting and alert about the potential spread and threats brought by these pathogens, as mentioned by the authors.<br>My main comments to improve this manuscript is that authors often goes on

conclusions that are not completely supported by the results. I suggest therefore to reconsider these conclusions or change the way they are presented to stick more on the facts.
We took into consideration all the comments regarding the conclusions and we made sure to do all the required modifications.

Furthermore, the GenBank accession for the Tef1-α sequences are not available which hamper my reviewing conclusions. This is even more problematic because the genetic difference observed to discriminate the new species are brought by this marker.
The ITS and the tef1-α sequences have been deposited into GenBank; However, the tef1-α sequences are not automatically deposited into GenBank after being accessioned. Each sequence record is individually examined and processed by the GenBank annotation staff to ensure that it is free of errors or problems.
In this sense, a new species (Lasiodiplodia mitidja) is introduced in this study. This introduction is based on a two loci phylogeny, as well as morphological observations. I'm not a taxonomist myself, but are two SNPs (which I could not verified, and that is not illustrated by an alignment neither in the manuscript) and a bootstrap of 80 enough to consider the organism a new species? Concerning the morphological differences, as the authors mentioned, conidia "tend" to be larger and L/W ratio is different but for both measurements, no statistical significance is brought to the observations to confirm the difference. Can it represent a subpopulation of L. citricola? I presume it is not possible to test if those "species" outcross but if we have to be more rigorous, I would recommend to stay more prudent about the "new species" terminology and presented it more as a suggestion, or inform the readers that all the criteria to say it's a new species are not completely fulfil.
We agree that this may be debatable. In fact, we have discussed this previously within the team. However, it is clearly aligned with the current trend for introduction of novel Lasiodiplodia species.
In the future it may well be proven that in fact it is not a new species different from L. citricola. But taxonomy is dynamic and hence frequently changing. For the moment we would like to introduce the new species. The fact is that eventually it will be described as a new species, if not by our group, then by someone else.
As an example of a case which is similar to ours, here are the nucleotide differences for the following mentioned species: L. chinensis vs L. lignicola vs L. pseudotheobromae. For all 3 species ITS is 100 % identical
As for Tef1:
L. chinensis vs L. lignicola: 1 nucleotide difference
L. chinensis vs L. pseudotheobromae: 3 nucleotides differences
My second concern is the ambiguity made between the types of wood alteration/symptoms observed in Citrus trees and the presence and implication of Botryosphaeriaceae. Botryosphaeriaceae species can be isolated from certain types of alteration and yet not being responsible of these alterations. Knowing the "opportunist" behaviour of these pathogens, I would not be surprised if they take over the habitat after a disequilibrium was induced into the microbiome of trees following another pathogen attack.
We agree with the reviewer. After considering your other comments on the same part and your suggestion of removing it and given that it is not relevant for the paper we decided to delete it.

The fact that the isolates were able to provoke symptoms experimentally does not necessarily mean they are responsible of the ones observed on the diseased trees, especially since the symptoms observed after artificial inoculation are not correlated to the ones observed on fields.
We agree with the reviewer, however the goal was to test pathogenicity of the isolates and this is the way to do it. Of course we cannot be sure that they will behave the same way in the field but they have the potential to do so.
Similarly, the presence of basidiocarps on heavily symptomatic Citrus is confusing for me, at least the way it is presented. What is the link between the Botryosphaeriaceae and the basidiocarps emergence, which species correspond to this basidiocarp?
We described the health status of the orchards where the sampling has been carried out (branch and shoot cankers, abnormal growth of epicormic shoots; defoliation and leaf chlorosis). Basidiocarps are the fruiting bodies of the decay-causing fungi. Their presence on the trunk means that it is an already rotting trunk and that some

Powered by Editorial Manager® and ProduXion Manager® from Aries Systems Corporation

ascomycetes (Botryosphaeriaceae, Diatrypaceae…) and Basidiomycetes (Fomitiporia, Phellinus….) have already colonized the trunk.
In a similar fashion, Figure 4 is confusing as my conclusion on this figure is that Botryosphaeriaceae can be isolated from different types of symptoms and not that one species is more isolated from one type of symptoms than another as the authors tend to say. There is no statistic proving so, and a quick interpretation (but false) from a hurried reader would be that such species is responsible of such symptoms. At this point, those results are more detrimental that beneficial to the study. I either recommend to delete this part or erase those ambiguities by a deeper discussion and a clearer result presentation. What do we know about the multifactorial aspects of dieback diseases? Is there only one pathogen involved? I think study conducted on Botryosphaeriaceae and grapevine trunk disease can be related to this case. Furthermore, if this part is conserved, more insights on what is known about the different symptoms that can cause Botryosphaeriaceae could/should be presented in the introduction.
We agree with the reviewer and his comments. After pointing out these remarks we thoughtfully considered them and decided to delete the figure 4 as well as the paragraphs related to it.
Finally, the statistical methods used to test pathogenicity differences is either not well presented or the conclusions are not correct. This part needs to be improve. Have you tested species effect? Isolate effect? What are the p-value attributed to each ANOVA test, which factor has been tested by the ANOVA? Is LSD method (which is not described by the way) the more appropriate in your case?
We took into consideration your valuable comments and we made sure to change this part and we removed all the ambiguities.

Minor Comments:

L30: 14.1% percent of the samples and 13% of the samples
R: Revised as recommended
L31: what is the difference between widespread and abundant?
R: Widespread means that it is found or distributed over a large area. However, abundant means that it is existing or available in large quantities (it could have the same meaning as plentiful)

L42: I would erase (pomelos)
R: Revised as recommended

L43: Despite the high adaptation capacity of citrus trees to different climates (reference is missing)
R: Revised as recommended

L47: Citrus diseases are numerous and diverse, and are caused by phytopathogenic agents belonging to viruses, viroids, phytoplasmas, bacteria, and fungi (reference is missing).
R: Revised as recommended

L57: reference is missing
R: Revised as recommended
L63: colonize or affect?
R: We deleted ‘affect’

This part on Botryosphaeriaceae should be more documented: classification of Botryosphaeriaceae, how many genera, endophytes with symptomless period, etc…
R: Revised as recommended
L82: Surveys were conducted in ten commercial orchards in the northern region of Algeria, specifically, in the Mitidja plain at the base of the Tell Atlas Mountains (Table 1).

Table 1: I would add coordinates of the orchards and years of sampling.
R: Revised as recommended

L94: was the scalpel sterilized?
R: Yes, the scalpel was sterilized. This detail has been added to the manuscript.

Powered by Editorial Manager® and ProduXion Manager® from Aries Systems Corporation

L112: In this paragraph, can you add more infos about the PCR conditions?
R: Revised as recommended

L120-121: pyrosequencing?
R: the company used Sanger sequencing method.

L123: Newly generated sequences were deposited in GenBank (Table 2): the Tef1-α isn't accessible.
R: The sequences are available in GenBank.

L124: Sequences for both DNA regions were retrieved in BLAST searches from GenBank [34].
Check the meaning of this sentence. For example: "Homological sequences of the newly sequenced ones were retrieved from the GenBank by Blast."
R: Revised as recommended

L125: Table 2: Can you add more info on this table, like type of tissue (trunk/branches), type of symptoms (under your classification), Orchard, etc.…
R: Revised as recommended

L132: Please specify the request you made, or put the sequence in the supplemental files.
R: Revised as recommended (the sequences are in the supplementary files)

L136: what kind of adjustments?
R: The ITS and tef1-α sequences were initially aligned separately using ClustalX v. 1.83. The alignments were manually optimized by coding the missing sequences as "?". Ambiguous sequences at the start and the end were deleted and gaps were adjusted in BioEdit.
L153: diameter
R: Revised as recommended

L164: this part need to be more precise/improve. Which threshold to accept the significance of the ANOVA, which factor tested, what LSD means for, which soft did you use?
R: Revised as recommended
Detailed responses:
Which threshold to accept the significance of the ANOVA
R: when the P value is below the threshold (0.05), the difference between the means is considered as significant.

Which factor tested?
R: We tested the lesions produced by each fungal isolate of the different species.

What LSD means for?
R: We changed the statistical test by using Tukey's honestly significant difference (HSD) test.
Which soft did you use?
R: The R v. 3.5.1 statistical software was used to perform the statistical analysis.

L171: nothing is said about the distribution
R: We removed the word 'distribution' from the title

L172-173 and L174-175 could be fused for clarity purposes
R: We removed this paragraph as recommended.

L175: the total number of samples… Samples = branches?
R: The samples mean the different necrotic lesions found in the branches and the trunks of the 80 trees.
L176 – 177: this measurement is completely arbitrary and according to me abusive. If one pathogen would have occurred at 80% frequency, the difference between 11%(very frequent) and 4%(infrequent) would be meaningless. My advice, stick to the numbers and do not try to interpreted it in a frequent or infrequent way, that's too

subjective.
R: We removed the paragraph related to the frequency of occurrence, as recommended.

L186: On heavily infected trees, basidiocarps emerged: that's ambiguous, as said before.
R: We removed the description of the basidiocarps from the photoplate as well as from the text, as recommended.

L189: why wedge-shaped necrosis is not name WSN?
R: Revised as recommended

L191: similar BCN instead of BCN and YSW instead of NCC.
R: Revised as recommended

L196: the "e." is missing on the picture, but maybe see in it as a sign for not putting this picture…
R: Revised as recommended

L214: Why MP tree is not shown? At least in supplemental file?
R: Revised as recommended (the tree is in supplementary file)

L218: why 23 isolates and not 24 (10+14)
R: The sequence of one isolate was not good enough to use it for the phylogenetic analysis.

L228: The phylogenetic tree of only one method is presented, although the bootstrap values of the two methods are shown. Can we see the tree constructed with the second method in the supplemental files?
R: Revised as recommended

L279: can you have the sequence accessible please?
R: The sequence has been submitted to GenBank. It will be available online after verification of the annotation. We have included the accession number into the table 2.
L311: from what I've read, LSD test is not recommended anymore as sensitive to multiple comparison. Furthermore, as mentioned above, this test is not well conducted and presented. A histogram would be better, with a sign for significant difference, either at the isolate level and species level, with threshold use for significance. The 100% of re-isolation frequency for every isolates are not necessary in the table according to me, if you say it in the text.
R: Revised as recommended.
L317: Ambiguous: are you speaking of distribution in the wood? If yes, cut this paragraph in two: Frequency of occurrence / Distribution of Botryosphaeriaceae in the wood.
R: Revised as recommended

L320: I comment already the frequent and very frequent ranking, that's abusive according to me.
R: We deleted this paragraph.

L323: For each orchard, at least two different species were isolated, average per orchard?
R: In the paragraph L323, we described the widespread of the species in the orchards. It was only to mention their presence on each orchard.
For more details, here is a table containing all the information about species distribution among the surveyed orchards.
Region
Oued El AlleugChiffa BoufarikStaoueli
Species/ Orchards12345678910Total
D. seriata----21--3410
D. mutila----32----5
L. mediterr.2231--22--12
L. mitidjana0333--22--13
Doth. vitic.1-1-12--117

Total 357465444547

L328: new paragraph or no paragraph at all as mentioned above, I found this part confusing.
R: Revised as recommended (We removed the paragraph).

L370-371: According to these authors, L. mediterranea has been found only on V-shaped necrotic sectors of grapevine while it has been isolated from all the lesion types of citrus trees in this study.
R: Revised as recommended
L373: what the results of Andolfi et al. bring to your results?
R: Andolfi et al. (2016) isolated and characterized the main secondary metabolites produced by L. mediterranea. They also, evaluated its phytotoxic and antifungal activities. These findings support our results, which show the ability of this fungal species to colonize and cause damages in the wood.

L374-375: maybe if you had isolated only the 4 or 5 isolates that were from brown central necrosis, would you have said that the species was exclusively found in brown central necrosis? This part of the discussion is not really constructive.
R: we removed the paragraph, as well as the figure 4.

L387: More interesting that this, it confirms its wide geographical range.
R: revised as recommended

L402: Wedge shaped lesion?
R: The paragraph is about the pathogenicity trial and the lesions produced by each Botryosphaeriaceae species. We did not consider the shape of the lesions, for the pathogenicity test.

L403-405: this part of the discussion goes beyond what your data show, either improve your statistical analysis or moderate the message.
R: Revised as recommended

L408-415: similar, hard to have this kind of discussion with two isolates per strains, with one phenotyping trial.
R: Revised as recommended

L446: References have to be reformatted: species not in italic, capital letter on every first word letter for some references, etc.; some other problem like L508 Phililips is written bizarrely.
R: Revised as recommended

Answers to the reviewer 2
Reviewer #2: This is a nice study of Botryosphaeriaceae which are important plant pathogens, including fruit trees. Algeria is unexplored both from mycological and pathological perspective and it is nice to see collaborations like this resulting in a good piece of work. I agree with authors regarding Lasiodiplodia mediterranea and L.vitis situation, especially because pcr atifacts introduced by primer sequences are unfortunately a common thing these days (my personal experience). I have small suggestions that would improve the paper.

Abstract, line 25-of Botryosphaeriaeceae
R: Revised as recommended

Abstract, line 29- Delete which, add Lasiodiplodia mitidjana is described in this paper as a new species
R: Revised as recommended

Abstract, line 62- delete effect
R: Revised as recommended

Materials-line 98-dried on sterilized paper (towels, filter paper?)

Powered by Editorial Manager® and ProduXion Manager® from Aries Systems Corporation

R: Revised as recommended

Materials, line 100-The mycelium emerging from wood pieces was transferred…
R: Revised as recommended

Materials, line 105-Isolates that lacked pycnidia production…
R: Revised as recommended

Materials, line 150-How did you select representative isolates?
R: We selected two isolates, from each phylogenetically resolved species.

Materials, line 151-Shoots? But above you mentioned branches (line 148)
R: Revised as recommended (We have standardized using shoot instead of branch).

Materials, line 157-"… well watered and maintained under favorable conditions" What do you mean by this? Were the cuttings in soil or in water? How many times per week did you change the water? Ambient temperature? Light?
R: The inoculated cuttings were wrapped with wet sterile cotton to avoid the desiccation of the agar plug. The shoots were immediately transplanted into pots containing sterilized water as a growth substrate (10 shoots per pot), which were incubated at the ambient room temperature, under daily photoperiod. The water of the container was changed twice a week.

Results, line 182-Degrees of intensity? Where are they?
R: The degrees of intensity refer to the different levels of the dieback symptoms observed in the orchards.

Results, line 186-Basidiocarps of which species or genera?
R: We did not identify the basidiocarps. We described all the symptoms related to the citrus trees dieback, including the fruiting bodies emerged from the trunks.
Results, line 309-What about control plants?
R: We did not isolate any of the tested species from the negative control.

Results, line 313-Now you mention branches again
R: revised as recommended

Discussion, line 360-and seriously affected trees can become
R: revised as recommended

Discussion, lines 403-404- "However, D. seriata was significantly different compared to the rest of the isolates" But previously you said that both D. seriata and L. mediterranea were most aggressive species (based on lesion lengths). So which species was in fact most aggressive? Also, what about differences in aggressiveness between different isolates of the same species?
So which species was in fact most aggressive?
R: The significant difference was made based on a comparison between all the tested isolates. It was not about the pairwise comparisons that take one isolate and compare it with each of the rest of isolates. D. seriata and L. mediterranea were the most aggressive species when compared to the rest of the species. However, D. seriata was the most aggressive species, considering the length of the lesion for each isolate, separately.
Also, what about differences in aggressiveness between different isolates of the same species?
R: Significant variation in aggressiveness can occur within and among isolates from the same species. This aggressiveness refers to the quantitative variation of pathogenicity on the susceptible host infection efficiency, the latent period, the spore production rate and the infectious period of each strain. These components are closely related to the genetic variability within the strains of the same species.

Discussion, lines 408-412-Was this previous study also about Bot on citrus trees?
R: the study was about Lasiodiplodia species (Botryosphaeriaceae) on grapevine.

Discussion, lines 409 and 411-In line 404 you are talking about aggressiveness. Now about virulence. In line 413 you talk again about aggressiveness. Virulence and

Powered by Editorial Manager® and ProduXion Manager® from Aries Systems Corporation

	aggressiveness don't mean the same thing. Replace the term virulence with aggressiveness in lines 409 and 411.<br>R: Revised as recommended<br><br><br>Answers to the academic editor<br>L.273 ..we have shown…<br>R: Revised as recommended<br>L. 279 "…these 2 nt are not real." Are not real is confusing, maybe instead use " were correctly determined"?<br>R: Revised as recommended<br>L. 335 "The remaining species…" better mention the species' name here.<br>R: Revised as recommended<br>L. 351 A similar situation has been…<br>R: Revised as recommended<br>L. 363 worLd<br>R: Revised as recommended<br>L. 373 compared instead of comparing<br>R: Revised as recommended<br>L. 389 "…known for targeting economically important plants…" targeting sounds as if they are selecting the hosts based on the economical value. Maybe better say: …known to cause damage on several economically important species..."<br>R: Revised as recommended<br>l. 393 "The later…", better say "This latter species.." or just "It..."<br>R: Revised as recommended<br>L. 408 …with a previous study…<br>R: Revised as recommended<br>I would suggest to add some research perspectives at the end of the discussion. What kind of studies could help to better understand and develop management recommendations against citrus dieback?<br>R: Revised as recommended
**Additional Information:**	
**Question**	**Response**
**Financial Disclosure**<br><br>Enter a financial disclosure statement that describes the sources of funding for the work included in this submission. Review the submission guidelines for detailed requirements. View published research articles from *PLOS ONE* for specific examples.<br><br>This statement is required for submission and **will appear in the published article** if the submission is accepted. Please make sure it is accurate.	NO - Include this sentence at the end of your statement: The funders had no role in study design, data collection and analysis, decision to publish, or preparation of the manuscript.

**Unfunded studies**<br>Enter: *The author(s) received no specific funding for this work.*<br><br>**Funded studies**<br>Enter a statement with the following details:<br>• Initials of the authors who received each award<br>• Grant numbers awarded to each author<br>• The full name of each funder<br>• URL of each funder website<br>• Did the sponsors or funders play any role in the study design, data collection and analysis, decision to publish, or preparation of the manuscript?<br>• **NO** - Include this sentence at the end of your statement: *The funders had no role in study design, data collection and analysis, decision to publish, or preparation of the manuscript.*<br>• **YES** - Specify the role(s) played.<br><br>* typeset	
**Competing Interests**<br><br>Use the instructions below to enter a competing interest statement for this submission. On behalf of all authors, disclose any competing interests that could be perceived to bias this work—acknowledging all financial support and any other relevant financial or non-financial competing interests.<br><br>This statement **will appear in the published article** if the submission is accepted. Please make sure it is accurate. View published research articles from *PLOS ONE* for specific examples.	The authors have declared that no competing interests exist.

Powered by Editorial Manager® and ProduXion Manager® from Aries Systems Corporation

**NO authors have competing interests**<br><br>Enter: *The authors have declared that no competing interests exist.*<br><br>**Authors with competing interests**<br><br>Enter competing interest details beginning with this statement:<br><br>*I have read the journal's policy and the authors of this manuscript have the following competing interests: [insert competing interests here]*<br><br>* typeset	
**Ethics Statement**<br><br>Enter an ethics statement for this submission. This statement is required if the study involved:<br><br>• Human participants<br>• Human specimens or tissue<br>• Vertebrate animals or cephalopods<br>• Vertebrate embryos or tissues<br>• Field research<br><br>Write "N/A" if the submission does not require an ethics statement.<br><br>General guidance is provided below. Consult the submission guidelines for detailed instructions. **Make sure that all information entered here is included in the Methods section of the manuscript.**	No specific permits were required for the described field studies. This study did not involve endangered or protected species.

Powered by Editorial Manager® and ProduXion Manager® from Aries Systems Corporation

**Format for specific study types**<br><br>**Human Subject Research (involving human participants and/or tissue)**<br>• Give the name of the institutional review board or ethics committee that approved the study<br>• Include the approval number and/or a statement indicating approval of this research<br>• Indicate the form of consent obtained (written/oral) or the reason that consent was not obtained (e.g. the data were analyzed anonymously)<br><br>**Animal Research (involving vertebrate animals, embryos or tissues)**<br>• Provide the name of the Institutional Animal Care and Use Committee (IACUC) or other relevant ethics board that reviewed the study protocol, and indicate whether they approved this research or granted a formal waiver of ethical approval<br>• Include an approval number if one was obtained<br>• If the study involved *non-human primates*, add *additional details* about animal welfare and steps taken to ameliorate suffering<br>• If anesthesia, euthanasia, or any kind of animal sacrifice is part of the study, include briefly which substances and/or methods were applied<br><br>**Field Research**<br><br>Include the following details if this study involves the collection of plant, animal, or other materials from a natural setting:<br>• Field permit number<br>• Name of the institution or relevant body that granted permission	
**Data Availability**<br><br>Authors are required to make all data underlying the findings described fully available, without restriction, and from the time of publication. PLOS allows rare exceptions to address legal and ethical concerns. See the PLOS Data Policy and FAQ for detailed information.	Yes - all data are fully available without restriction

A Data Availability Statement describing where the data can be found is required at submission. Your answers to this question constitute the Data Availability Statement and **will be published in the article**, if accepted.<br><br>**Important:** Stating 'data available on request from the author' is not sufficient. If your data are only available upon request, select 'No' for the first question and explain your exceptional situation in the text box.<br><br>Do the authors confirm that all data underlying the findings described in their manuscript are fully available without restriction?	
**Describe where the data may be found in full sentences. If you are copying our sample text, replace any instances of XXX with the appropriate details.**<br><br>• If the data are **held or will be held in a public repository**, include URLs, accession numbers or DOIs. If this information will only be available after acceptance, indicate this by ticking the box below. For example: *All XXX files are available from the XXX database (accession number(s) XXX, XXX.*).<br>• If the data are all contained **within the manuscript and/or Supporting Information files**, enter the following: *All relevant data are within the manuscript and its Supporting Information files.*<br>• If neither of these applies but you are able to provide **details of access elsewhere**, with or without limitations, please do so. For example:<br><br>*Data cannot be shared publicly because of [XXX]. Data are available from the XXX Institutional Data Access / Ethics Committee (contact via XXX) for researchers who meet the criteria for access to confidential data.*<br><br>*The data underlying the results presented in the study are available from (include the name of the third party*	All relevant data are within the manuscript and its Supporting Information files.

and contact information or URL).<br>• This text is appropriate if the data are owned by a third party and authors do not have permission to share the data.<br><br>* typeset	
Additional data availability information:	Tick here if the URLs/accession numbers/DOIs will be available only after acceptance of the manuscript for publication so that we can ensure their inclusion before publication.

Powered by Editorial Manager® and ProduXion Manager® from Aries Systems Corporation

Manuscript Click here to access/download;Manuscript;Manuscript.docx

Lasiodiplodia mitidjana sp. nov. and other *Botryosphaeriaceae* species causing branch canker and dieback of *Citrus sinensis* in Algeria

Akila Berraf-Tebbal[1*], Alla Eddine Mahamedi[2,3], Wassila Aigoun-Mouhous[2,4], Milan Špetík[1], Jana Čechová[1], Robert Pokluda[1], Miroslav Baránek[1], Aleš Eichmeier[1], Artur Alves[5]

[1] Mendel University in Brno, Faculty of Horticulture, Mendeleum - Institute of Genetics, Valticka, Lednice, Czech Republic

[2] Laboratoire de Biologie des Systèmes Microbiens (LBSM), Département des Sciences Naturelles, Ecole Normale Supérieure de Kouba-Alger, Alger, Algeria

[3] Département de Biologie, Faculté des Sciences de la Nature et de la Vie et Sciences de la Terre, Université de Ghardaïa, Ghardaïa, Algeria

[4] Département des Biotechnologies, Faculté des Sciences de la Nature et de la Vie, Université de Blida, Blida, Algeria

[5] Departamento de Biologia, CESAM, Universidade de Aveiro, Aveiro, Portugal

* Corresponding author
E-mail: qqberraf@mendelu.cz

Abstract

Several *Botryosphaeriaceae* species are known to occur worldwide, causing dieback, canker and fruit rot on various hosts. Surveys conducted in ten commercial citrus orchards in the northern region of Algeria revealed five species of *Botryosphaeriaceae* belonging to three genera associated with diseased trees. Morphological and cultural characteristics as well as phylogenetic analyses of the internal transcribed spacer (ITS) region and the translation elongation factor 1-alpha (*tef1*-α) identified *Diplodia mutila*, *Diplodia seriata*, *Dothiorella viticola*, *Lasiodiplodia mediterranea* and a novel species which is here described as *Lasiodiplodia mithidjana* sp. nov.. Of these, *L. mithidjana* (14.1% of the samples) and *L. mediterranea* (13% of the samples) were the most widespread and abundant species. Pathogenicity tests revealed that *L. mediterranea* and *D. seriata* were the most aggressive species on citrus shoots. This study highlights the importance of *Botryosphaeriaceae* species as agents of canker and dieback of citrus trees in Algeria.

Key words: Citrus cultivation, trunk diseases, fungi, identification, taxonomy, pathogenicity.

Introduction

Citrus cultivation is one of the major contributors to Algerian wealth and is part of the traditional agriculture of the country. Many types of citrus are grown in Algeria, including oranges (48 400 ha), clementine (10 817 ha), mandarins (2 347 ha), lemons (4 409 ha) and grapefruits (83 ha) [1]. Despite the high adaptation capacity of citrus trees to different climates [2], a number of unfavourable factors has led to a decrease of the total citrus yield in Algeria. Among these factors, ageing trees, droughts, inappropriate cultural practices and the effects of various pests and pathogens are the most important [2, 3]. Citrus diseases are numerous and diverse, and are caused by phytopathogenic agents belonging to viruses, viroids, phytoplasmas, bacteria, and fungi [2] Some pathogens cause very serious diseases, predisposing to, and inciting dieback, while others are less serious [2-5].

Recently, trunk diseases have become a growing threat in both, old and newly established orchards of citrus, worldwide. Symptoms include leaves that become yellow and fall early, shoots and twigs die, increasing the risk of citrus decay as the damage expands to the trunk [2, 6-8]. To date, among the fungi that impact citrus, *Diaporthe* species are well known for causing stem-end rot and melanose of fruits, young leaf and shoot gummosis and blight of perennial branches and trunks, in Greece, Italy, Malta, Portugal, Spain, China, Korea, New Zealand, and the USA [8-11]. *Fusarium* and *Neocosmospora* have also been reported causing canker and dieback diseases of citrus, in Tunisia, Greece, Italy and Spain [12-14]. The *Diatrypaceae* are other canker and dieback pathogens impacting citrus orchards [15]. Several *Eutypella* spp. have been reported from *Citrus* sp. In southern California desert, three distinct species of *Eutypella* are found associated with citrus branch canker, namely: *Eutypella citricola*, *E. microtheca* and a *Eutypella* sp. [15-17].

In addition to the above fungal pathogens that compromise citrus crops, several *Botryosphaeriaceae* species are known to colonize citrus trees. The *Botryosphaeriaceae* family is recognized as an important and widely distributed plant pathogen, which impacts on a variety of economically important hosts. It comprises 24 genera encompassing 222 species, living as endophytes, saprobes, or plant pathogens [18, 19]. Recent studies carried out in California, Italy and Tunisia have highlighted the *Botryosphaeriaceae* as the most prevalent fungi that cause cankers, vascular necrosis and dieback of citrus trees [7, 15, 16, 20]. Adesemoye et al. [21] recovered various *Botryospahriaceae* species from necrotic tissues of citrus branch canker and rootstock, including, *Diplodia seriata*, *D. mutila*, *Dothiorella viticola*, *D. iberica*, *Lasiodiplodia parva*, *Neofusicoccum australe*, *N. luteum*, *N. mediterraneum*, *N. parvum* and *Neoscytalidium dimidiatum*. In Iran, Abdollahzadeh et al. [22], described *Lasiodiplodia citricola* from citrus trees showing symptoms of branch dieback.

In Algeria, members of the *Botryosphaeriaceae* have been reported to cause diseases on *Vitis vinifera* [23-25], *Quercus suber* [26] and *Cupressus macrocarpa* [27]. Linaldeddu et al. [28] isolated and described *Lasiodiplodia mediterranea* from a cankered branch of *Citrus sinensis* trees in northern Algeria. However, the impact of *Botryosphaeriaceae* species on citrus trees has not been studied in detail. Therefore, the aim of this study was to investigate

and determine the incidence of the *Botryosphaeriaceae* species associated with branch canker and dieback in the major citrus-growing region of Algeria.

Materials and Methods

Ethics Statement

No specific permits were required for the described field studies. This study did not involve endangered or protected species.

Field survey and sampling

Surveys were conducted in ten commercial orchards in the northern region of Algeria. Specifically, in the Mitidja plain at the base of the Tell Atlas Mountains. The sampling was done in four municipalities; namely Oued El Aleug (4 orchards), Chiffa (2 orchards), Boufarik (2 orchards) and the coastal town, Sidi Fredj, located within the territory of the Staoueli municipality, situated by the Mediterranean Sea (2 orchards). The field diagnosis and sampling were performed between April 2013 and March 2015. Samples were collected from the orchards with permission of landowners. Trunks and branches showing symptoms such as dead shoots, defoliation, cankers, wood necrosis, and dieback were collected, randomly. A total of 80 symptomatic sweet orange (*Citrus sinensis*) trees were sampled (Table 1).

Table 1. Citrus orchards surveyed and number of samples collected.

Locality	GPS coordinates	Orchards	Area (ha)	Number of trees sampled	Number of samples processed
Oued El Alleug	36°33'21"N 2°47'22"E	a	18	5	9
		b	16	5	6
		c	28	5	7
		d	6.8	5	6
Chiffa	36°27'44"N 2°44'27"E	a	25	10	13
		b	18	10	10
Boufarik	36°34'31"N 2°54'46"E	a	43	10	10
		b	27	10	11
Staoueli	36°45'12"N 2°53'17"E	a	32	10	10
		b	15	10	10

Fungal isolation and morphological characterization

In the laboratory, all samples were processed by peeling the outer bark surface with a sterilized scalpel. Longitudinal and transversal cuts were made to reveal the type and localization of the internal necrosis. From each lesion detected, ten pieces of wood, approx. 5 mm^2, were cut from the margins between necrotic and healthy tissues. These pieces were submerged in 4 % sodium hypochlorite for 15 min, washed thrice with sterile distilled water, dried with sterilized filter paper and placed onto the surface of potato dextrose agar (PDA, Difco Laboratories). Plates were incubated at 25 °C until growth was detected. The mycelium emerging from wood pieces were transferred onto fresh PDA plates and incubated under the same conditions.

Preliminary identifications to genus and tentative species level were based on colony and conidial morphology (colony colour, colony growth pattern, conidial size, shape, colour, striation, septation, conidiogenous cells, and presence of paraphyses) according to Phillips et al. [18]. Isolates that lacked pycnidia production on PDA were placed on autoclaved pine needles in ¼ strength PDA within 2–3 weeks, incubated at 25 °C under mixed near-UV and cool-white fluorescent light in a 12 h light 12 h dark regime for 2–6 weeks, to enhance fruiting body production. Conidiogenous layer and conidia were mounted in 100 % lactic acid and observed with a Nikon 80i light microscope.

DNA extraction, PCR amplification and sequencing

Total genomic DNA was extracted from 7 days old axenic cultures, grown on PDA at 25 °C, following Santos and Phillips [29]. PCR reactions were carried out with *Taq* DNA polymerase, nucleotides and buffers supplied by MBI Fermentas (Vilnius, Lithuania). PCR reaction mixtures were prepared as previously described by Alves et al. [30], with the addition of 5 % DMSO to improve the amplification of some difficult DNA templates. The ITS region plus D1/D2 domain of the LSU was amplified with the primer pair ITS1 [31] and NL4 [32]. The amplification conditions were initial denaturation of 5 min at 95 °C, followed by 29 cycles of 30 s at 94 °C, 30 s at 50 °C, and 1.5 min at 72 °C, and a final extension of 10 min at 72 °C. Part of the translation elongation factor 1 alpha gene (*tef1*-α) was amplified with primers EF1-688F and EF1-1251R [33]. The amplification conditions were: initial denaturation of 5 min at 95 C, followed by 30 cycles of 30 s at 94 °C, 45 s at 55 °C, 1½ min at 72 °C, and a final extension period of 10 min at 72 °C. ITS and *tef1*-α regions were sequenced in both directions by STAB Vida Lda (Portugal), using the Sanger method.

The nucleotide sequences were read and edited with BioEdit Alignment Editor V.7.0.9.0 [34]. Newly generated sequences were deposited in GenBank (Table 2). Homological sequences of the newly sequenced ones were retrieved from the GenBank using the Basic Local Alignment Search Tool (BLAST) [35].

Table 2. ***Botryosphaeriaceae*** **species included in this study**

**Species**	**Isolate number**	**Host/ Substrate**	**Origin**	**Collector**	**GenBank accession numbers**	
					**ITS**	***tef1-α***
Lasiodiplodia mediterranea	ALG77	*Citrus*/wood canker	Algeria, Boufarik	Akila Berraf-Tebbal	MN104094	MN159093
L. mediterranea	ALG76	*Citrus*/wood canker	Algeria, Oued El Alleug	Akila Berraf-Tebbal	MN104095	MN159094
L. mediterranea	ALG40	*Citrus*/wood canker	Algeria, Oued El Alleug	Akila Berraf-Tebbal	MN104096	MN159095
L. mediterranea	ALG78	*Citrus*/wood canker	Algeria, Oued El Alleug	Akila Berraf-Tebbal	MN104097	MN159096
L. mediterranea	ALG41	*Citrus*/wood canker	Algeria, Oued El Alleug	Akila Berraf-Tebbal	MN104098	MN159097
L. mediterranea	ALG36	*Citrus*/wood canker	Algeria, Oued El Alleug	Akila Berraf-Tebbal	MN104099	MN159098
L. mediterranea	ALG80	*Citrus*/wood canker	Algeria, Oued El Alleug	Akila Berraf-Tebbal	MN104100	MN159099
L. mediterranea	ALG73	*Citrus*/wood canker	Algeria, Boufarik	Akila Berraf-Tebbal	MN104101	MN159100
L. mediterranea	ALG74	*Citrus*/wood canker	Algeria, Boufarik	Akila Berraf-Tebbal	MN104102	MN159101
L. mediterranea	ALG75	*Citrus*/wood canker	Algeria, Boufarik	Akila Berraf-Tebbal	MN104103	MN159102
L. mediterranea	CBS 124060	*Vitis*, wood fragment	Italy, Sicily	S. Burruano	KX464148	MN938928
L. mitidjana	ALG81	*Citrus*/wood canker	Algeria, Oued El Alleug	Akila Berraf-Tebbal	MN104104	MN159103
L. mitidjana	ALG44	*Citrus*/wood canker	Algeria, Oued El Alleug	Akila Berraf-Tebbal	MN104105	MN159104

L. mitidjana	ALG39	*Citrus*/wood canker	Algeria, Oued El Alleug	Akila Berraf-Tebbal	MN104106	MN159105
L. mitidjana	ALG42	*Citrus*/wood canker	Algeria, Oued El Alleug	Akila Berraf-Tebbal	MN104107	MN159106
L. mitidjana	ALG38	*Citrus*/wood canker	Algeria, Oued El Alleug	Akila Berraf-Tebbal	MN104108	MN159107
L. mitidjana	ALG43	*Citrus*/wood canker	Algeria, Oued El Alleug	Akila Berraf-Tebbal	MN104109	MN159108
L. mitidjana	ALG37	*Citrus*/wood canker	Algeria, Oued El Alleug	Akila Berraf-Tebbal	MN104110	MN159109
L. mitidjana	ALG34	*Citrus*/wood canker	Algeria, Oued El Alleug	Akila Berraf-Tebbal	MN104111	MN159110
L. mitidjana	ALG82	*Citrus*/wood canker	Algeria, Oued El Alleug	Akila Berraf-Tebbal	MN104112	MN159111
L. mitidjana	ALG72	*Citrus*/wood canker	Algeria, Boufarik	Akila Berraf-Tebbal	MN104113	MN159112
L. mitidjana	ALG71	*Citrus*/wood canker	Algeria, Boufarik	Akila Berraf-Tebbal	MN104114	MN159113
L. mitidjana	ALG70 = MUM 19.90	*Citrus*/wood canker	Algeria, Boufarik	Akila Berraf-Tebbal	MN104115	MN159114
L. mitidjana	ALG69	*Citrus*/wood canker	Algeria, Boufarik	Akila Berraf-Tebbal	MN104116	MN159115
Diplodia seriata	ALG93	*Citrus*/wood canker	Algeria, Staoueli	Akila Berraf-Tebbal	MN104117	MN159116
D. seriata	ALG94	*Citrus*/wood canker	Algeria, Staoueli	Akila Berraf-Tebbal	MN104118	MN159117
D. seriata	ALG98	*Citrus*/wood canker	Algeria, Staoueli	Akila Berraf-Tebbal	MN104119	MN159118
D. seriata	ALG91	*Citrus*/wood canker	Algeria, Chiffa	Akila Berraf-Tebbal	MN104120	MN159119
D. seriata	ALG92	*Citrus*/wood canker	Algeria, Staoueli	Akila Berraf-Tebbal	MN104121	MN159120

D. seriata	ALG90	*Citrus*/wood canker	Algeria, Chiffa	Akila Berraf-Tebbal	MN104122	MN159121
D. seriata	ALG89	*Citrus*/wood canker	Algeria, Chiffa	Akila Berraf-Tebbal	MN104123	MN159122
D. seriata	ALG96	*Citrus*/wood canker	Algeria, Staoueli	Akila Berraf-Tebbal	MN104124	MN159123
D. seriata	ALG95	*Citrus*/wood canker	Algeria, Staoueli	Akila Berraf-Tebbal	MN104125	MN159124
D. seriata	ALG97	*Citrus*/wood canker	Algeria, Staoueli	Akila Berraf-Tebbal	MN104126	MN159125
D. mutila	ALG99	*Citrus*/wood canker	Algeria, Chiffa	Akila Berraf-Tebbal	MN104127	MN159126
D. mutila	ALG103	*Citrus*/wood canker	Algeria, Chiffa	Akila Berraf-Tebbal	MN104128	MN159127
D. mutila	ALG100	*Citrus*/wood canker	Algeria, Chiffa	Akila Berraf-Tebbal	MN104129	MN159128
D. mutila	ALG102	*Citrus*/wood canker	Algeria, Chiffa	Akila Berraf-Tebbal	MN104130	MN159129
D. mutila	ALG101	*Citrus*/wood canker	Algeria, Chiffa	Akila Berraf-Tebbal	MN104131	MN159130
Dothiorella viticola	ALG83	*Citrus*/wood canker	Algeria, Staoueli	Akila Berraf-Tebbal	MN104087	MN159086
Doth. viticola	ALG35	*Citrus*/wood canker	Algeria, Oued El Alleug	Akila Berraf-Tebbal	MN104088	MN159187
Doth. viticola	ALG84	*Citrus*/wood canker	Algeria, Chiffa	Akila Berraf-Tebbal	MN104089	MN159188
Doth. viticola	ALG85	*Citrus*/wood canker	Algeria, Staoueli	Akila Berraf-Tebbal	MN104090	MN159189
Doth. viticola	ALG86	*Citrus*/wood canker	Algeria, Oued El Alleug	Akila Berraf-Tebbal	MN104091	MN159190
Doth. viticola	ALG87	*Citrus*/wood canker	Algeria, Chiffa	Akila Berraf-Tebbal	MN104092	MN159191

Doth. viticola	ALG88	*Citrus*/wood canker	Algeria, Chiffa	Akila Berraf-Tebbal	MN104093	MN159192

Phylogenetic analysis

Sequences of all *Lasiodiplodia* species known from culture were retrieved from GenBank (S1 Table) and aligned with sequences of the isolates obtained in this study. Alignments were done with ClustalX v. 1.83 [36] using the following parameters: pairwise alignment parameters (gap opening = 10, gap extension = 0.1) and multiple alignment parameters (gap opening = 10, gap extension = 0.2, transition weight = 0.5, delay divergent sequences = 25%). Alignments were checked and manual adjustments made if necessary using BioEdit v. 7.2.5 [34]. Maximum Likelihood (ML) and Maximum Parsimony (MP) analyses were performed using MEGAX [37]. The best fitting DNA evolution model was determined also by MEGAX. ML analysis was performed on a Neighbour-Joining starting tree automatically generated by the software. Nearest-Neighbour-Interchange (NNI) was used as the heuristic method for tree inference. MP analysis was done using the Tree-Bisection-Regrafting (TBR) algorithm with search level 1 in which the initial trees were obtained by the random addition of sequences (10 replicates). The robustness of the trees (ML and MP) was evaluated by 1000 bootstrap replications.

Pathogenicity test

The ability of isolates to cause cankers was assessed *in vivo* on detached shoots collected from symptomless citrus trees. From each phylogenetically resolved species, two representative isolates were selected. Pathogenicity of each selected strain was tested on 1-year-old shoots of *Citrus sinensis*. The shoots with 25 mm in diameter were cut into equal length (25 cm long). They were then surface disinfected with 70% ethanol and wounded on an intermediate internode, with a scalpel. From each strain, a 5 mm diameter mycelial plug taken from a 5- day old colony growing on PDA was placed into the wound. Negative controls were inoculated with fresh, non-colonized, PDA plugs.
Subsequently, the cuttings were wrapped with wet sterile cotton and sealed with Parafilm® to prevent the desiccation of the agar plug. The shoots were immediately transplanted into pots containing sterilized water as a growth substrate. They were incubated at the ambient room temperature, under daily photoperiod. The water of the container was changed twice a week. There were 10 replicates per isolate, and the same number of cuttings was used as controls. One month after inoculation, lengths of lesions produced by each strain were measured. In an attempt to recover the inoculated fungi and complete Koch's postulates, necrotic tissue from the margin of the lesions was taken and placed onto PDA.

Statistical analyses

Data of lesion lengths caused by the fungal isolates belonging to the different species was subjected to one-way ANOVA (analysis of variance) with $P \leq 0.05$. Prior to analysis data were checked for normality, then, significance of differences between means was determined by

Turkey's honestly significant difference (HSD) test. Statistical analyses were performed on the software R v. 3. 5. 1 and a significance level of 0.05 was used.

Nomenclature

The electronic version of this article in Portable Document Format (PDF) in a work with an ISSN or ISBN will represent a published work according to the International Code of Nomenclature for algae, fungi, and plants, and hence the new names contained in the electronic publication of a PLOS article are effectively published under that Code from the electronic edition alone, so there is no longer any need to provide printed copies.

In addition, new names contained in this work have been submitted to MycoBank from where they will be made available to the Global Names Index. The unique MycoBank number can be resolved and the associated information viewed through any standard web browser by appending the MycoBank number contained in this publication to the prefix http://www.mycobank.org/MB/. The online version of this work is archived and available from the following digital repositories: [INSERT NAMES OF DIGITAL REPOSITORIES WHERE ACCEPTED MANUSCRIPT WILL BE SUBMITTED (PubMed Central, LOCKSS etc)].

Results

Disease symptoms

Citrus dieback was detected in all the orchards and regions investigated, with different degrees of intensity. Various external symptoms, including partial or complete dieback of the tree, branch and shoot cankers, abnormal growth of epicormic shoots; defoliation and leaf chlorosis were observed. Moreover, in certain orchards, bark cracking of the trunk and the branches was also noticeable (Fig 1).

The analysis of the 80 symptomatic sweet orange (*Citrus sinensis*) trees sampled to carry out the isolations, revealed the existence of 92 necrotic lesions in the trunks and the branches. They belonged to four types of wood alteration, including: wedge-shaped necrosis (WSN), that was the most prevalent lesion (n=30) of the total samples collected. The brown central necrosis (BCN) (n=26) was the second most prevalent lesion, followed by the black spots in the xylem (BS) (n=24) and yellow soft wood rot (YSW) (n=12).

Fig 1. Citrus tree with dieback symptoms (a), bark cracking of the trunk and gummosis (b), main internal symptoms of sectioned branches and trunks (c–f).

Fungal isolation and identification

Isolation carried out from ninety-two samples yielded a total of forty-seven fungal colonies belonging to *Botryosphaeriaceae*. On the basis of morphological characteristics, it was possible to distinguish three morphological groups according to colour and shape of conidia. Twenty-five isolates with brown sub-globose and striate conidia were grouped as *Lasiodiplodia*-like fungi. Fifteen isolates with brown oblong to ovoid conidia as *Diplodia*-like fungi. A further seven isolates with brown, ovoid thick walled and 1-septate conidia were considered as *Dothiorella*-like fungi. The identification of the isolates was confirmed by analysis of ITS and *tef1*-α sequences, which distinguished five separate species. The BLAST searches in GenBank showed 99–100% identity with reference sequences of representative isolates including that of the ex-type. The identified species were: *Diplodia seriata* (10 isolates), *Diplodia mutila* (5 isolates), *Dothiorella viticola* (7 isolates), *Lasiodiplodia mediterranea* (10 isolates) and a *Lasiodiplodia* sp. (14 isolates) that could not be assigned to any of the currently known species.

Phylogenetic analysis

Phylogenetic analysis was performed using ITS and *tef1*-α sequences. Fragments of approximately 500 and 300 bases were determined for ITS and *tef1*-α regions, respectively. The ML and the MP trees are presented in figure 2 and figure S1, respectively. The combined ITS and *tef1*-α dataset of *Lasiodiplodia* consisted of 23 isolates aligned with sequences of 69 isolates retrieved from GenBank, representing a selection of all known *Lasiodiplodia* and 2 outgroup taxa (*Diplodia seriata* CBS 112555 and *Diplodia mutila* CBS 112553). In the ML phylogenetic tree (figure 2), the isolates obtained in this study grouped in two clades. The first clade comprised 10 isolates, which clustered together, with the ex-type strain of *Lasiodiplodia mediterranea* (CBS 137783) and the ex-type strain of *Lasiodiplodia vitis* (CBS 124060) (Table S1), forming a single monophyletic group. The second group contained 14 isolates, which formed a distinct clade, with a high bootstrap support (ML/MP = 80/94), was considered to represent a distinct species, which is described here as *Lasiodiplodia mitidjana* sp. nov. (Fig 2).

Fig 2. Maximum likelihood tree generated from the combined analysis of ITS and *tef1*-α sequence data. ML/MP bootstrap values are given at the nodes. Support values less than 50 % are omitted or indicated with '–'. The tree was rooted to *Diplodia mutila* and *Diplodia seriata*.

Taxonomy

Lasiodiplodia mitidjana A. Alves, A.E. Mahamedi & A. Berraf-Tebbal **sp. nov.** (Fig 3) [urn:lsid:mycobank.org:names: MB 832823]. Algeria, Mitidja, isolated from a branch canker of *Citrus sinensis*, June 2015, Akila Berraf-Tebbal, HOLOTYPE AVE-F-7, a dried culture sporulating on pine needles twigs deposited in the Herbarium Universitatis Aveirensis

(AVE), culture ex-holotype MUM 19.90 (=ALG70). Other isolates examined are listed in Table 2.

Etymology: named after Mitidja where the fungus was discovered.

Sexual state: Not seen. Asexual state: Conidiomata stromatic, pycnidial, produced on pine needles on ¼ strength PDA within 2–3 wks, dark brown to black, covered with dense mycelium, superficial or immersed in the host becoming erumpent when mature, mostly uniloculate, solitary, globose, thick-walled. Paraphyses hyaline, cylindrical, thin-walled, initially aseptate, becoming septate when mature, rounded at apex. Conidiogenous cells holoblastic, discrete, hyaline, smooth, thin-walled, cylindrical, sometimes slightly swollen at the base. Conidia subovoid to ellipsoid-ovoid, apex rounded, occasionally tapering to truncate base, widest in middle to upper third, thick-walled, with granular content, initially hyaline and aseptate, remaining so for a long time, becoming dark brown and 1-septate, with longitudinal striations, (22.6–)27.7(−31.9) × (13.5–)16.7(−19.6) µm, 95 % confidence limits = 27.3–28 × 16.5–16.9 µm (av. of 125 conidia ± SD = 27.7 ± 1.9 × 16.7 ± 1.1 µm, L/W ratio = 1.7).

Cultural characteristics: Colonies on PDA with moderate to dense aerial mycelium, initially white to smoke-grey, turning greenish grey on the surface and reverse, becoming dark slate blue with age.

Cardinal temperatures for growth: Minimum <10 °C, maximum < 40 °C and optimum 25 – 35 °C, covering the medium surface (90 mm) before 7 days at 25 °C in the dark.

Habitat: Twigs and branches of *Citrus sinensis*.

Known geographic distribution: Algeria.

Notes: Phylogenetically it is very closely related to *L. citricola* being distinguished by three bp in the *tef1*-α locus. Conidia tend to be larger than those of *L. citricola*, 95 % confidence limits = 24.1–24.9 × 15–15.7 µm (av. ± S.D. = 24.5 ± 0.2 × 15.4 ± 1.8 µm) and have a lower L/W ratio = 1.6.

Lasiodiplodia mediterranea Linaldeddu, Deidda & Berraf-Tebbal **sp. nov.** (Linaldeddu et al. 2015. Fungal diversity 71:207)

MycoBank: MB 808356

Synonym: ***Lasiodiplodia vitis*** Yang & Crous, **sp. nov.** (Yang et al. 2017. Fungal Biology 121)

MycoBank: MB817635

Notes: Yang et al. [38] described *L. vitis* as a novel species, clearly distinct from the species recognised on *Vitis vinifera* in Italy. However, in their study Yang et al. [38] did not include any representative of *L. mediterranea* which was described by Linaldeddu et al. [28] from

several hosts, including *V. vinifera* in Italy. However, we have shown that *L. vitis* is phylogenetically indistinguishable from *L. mediterranea*. Their ITS sequences are 100% identical and the *L. vitis tef1*-α sequence deposited in GenBank differs from the *tef1*-α of *L. mediterranea* in 2 nt positions (1 missing G and a C instead of a T in the EF-986R primer binding region). We re-sequenced the *tef1*-α region of *L. vitis* CBS124060 (Table 2) using primers EF1-688F and EF1-1251R [33] which span a larger region than primers EF1-728F and EF-986R used by Yang et al. [38] and verified that these 2 nt are not real. There is no missing G in the *L. vitis* sequence and the C instead of a T is an artefact in *L. mediterranea* sequence introduced by the EF-986R primer sequence. Thus, the *tef1*-α region of *L. vitis* CBS124060 is 100% identical to the *tef1*-α sequence of *L. mediterranea*.

Fig 3. ***Lasiodiplodia mitidjana*. (a,b). Pycnidia formed on pine needles. (c). Conidiogenous layer with conidia developing on conidiogenous cells. (d). Conidia developing on conidiogenous cells and paraphyses. (e,f,i). Hyaline aseptate conidia. (g,h). Hyaline aseptate brown 1-septate conidia in two focal planes showing the striations on the inner surface of the wall. (j). Aseptate conidia, one becoming brown. (k,l). Brown 1-septate conidia in two focal planes to show the striations in the inner surface of the wall. Scale bars: e = 20 µm, c,d,f–j = 10 µm; k–l = 5 µm.**

Pathogenicity test

All the *Botryosphaeriaceae* isolates tested in the pathogenicity test were pathogenic to the citrus shoots. On the wood tissue under the bark, black to brown lesions developed, upward and downward from the inoculation point, within 30 days. The control plants did not develop any symptoms. Lesion lengths varied between the species and among the isolates of each species tested, with a significant difference ($F=10.874$; $P < 0.001$) (Table 3).

The most aggressive isolates were ALG91 (*D. seriata*) and ALG36 (*L. mediterranea*), which produced the longest lesions (5.49±2.65 cm and 4.39±1.31 cm, respectively) with a statistically significant difference recorded between ALG91 and the rest of the species, except for *L. mediterranea*. No significant difference in lesions size was observed between the isolates ALG40 (*L. mediterranea*) and ALG39 (*L. mitidjana*), which presented intermediate lesion lengths (3.83±0.97 and 3.88±1.24 cm, respectively). However, the smallest lesion size was produced by *Doth. viticola* ALG84 with 2.1±0.67 cm and both *D. mutila* isolates ALG102 (2.04±0.54) and ALG103 (2.05±0.4 cm). *D. seriata* was the only species that showed significant difference in lesion length between its two isolates (Table 3).

Koch's postulates were confirmed by a successful re-isolation of all tested fungal species from the necrotic tissues (Table 3).

Table 3. Mean lesion lengths (cm) caused by *Doth. viticola, D. mutila, D. seriata, L. mediterranea* and *L. mitidjana* species implicated in citrus dieback in northern Algeria,

30 days after inoculation of detached green branches with mycelium-colonized agar plugs.

**Species**	**Isolate**	**Mean lesion length (cm) ± SD**
D. seriata	ALG91	5.49±2.65 a
D. seriata	ALG98	2.35±0.55 cd
L. mediterranea	ALG36	4.39±1.31 ab
L. mediterranea	ALG40	3.83±0.97 abc
Doth. viticola	ALG86	2.82±0.86 bcd
Doth. viticola	ALG84	2.1±0.67 d
D. mutila	ALG102	2.04±0.54 d
D. mutila	ALG103	2.03±0.29 d
L. mitidjana	ALG39	3.88±1.24 abc
L. mitidjana	ALG34	2.2±0.67 cd

The same letter after numbers refers to the isolates that do not differ significantly according to Turkey's HSD test at $P \leq 0.05$.

Distribution of *Botryosphaeriaceae* species

Overall, the *Botryosphaeriaceae* species occurred in 42 of the 80 citrus trees showing canker and dieback symptoms. Five distinct *Botryosphaeriaceae* species were obtained in this study. Each species was found with its respective frequency, as follow: *L. citricola* (14.1%), *L. mediterranea* (13%) and *D. seriata* (10.9%), *Doth. viticola* (7.6%) and *D. mutila* (5.4%).

At least, two different species were found in each orchard. *L. mediterranea* and *Doth. viticola* were found in six of the ten surveyed orchards. They were followed by *L. mitidjana*, recorded from five orchards of two municipalities. *D. seriata* was found in four sampling sites; whereas, *D. mutila* was recovered from only two orchards of the same municipality.

Discussion

This study aimed to evaluate and characterize the diversity of the *Botryosphaeriaceae* species associated with dieback of *Citrus sinensis*. It represents the first survey and preliminary investigation of these species in the main citrus orchards in northern Algeria.

Citrus canker and dieback were detected in all regions surveyed. Several external symptoms, including partial or complete dieback of the tree, branch and shoot cankers were observed. Over time, the disease can increase and seriously affected trees can become barren and eventually, die. Similar situation has been described in several citrus orchards, worldwide [7, 15, 20, 21, 39]. According to some authors, abiotic factors, including drought, severe sunburn or freezing predispose the trees to xylem dysfunction, leading to these diseases [2, 3, 39].

In this study, five species belonging to three different genera of the *Botryosphaeriaceae* were recovered from symptomatic citrus trees, namely: *L. mediterranea*, *D. seriata*, *D. mutila*, *Doth. viticola* and *L. mitidjana*. The latter is introduced here, as new species. To our knowledge, except for *D. seriata* and *L. mediterranea*, this is the first report of *D. mutila*, *Doth. viticola* and *L. mitidjana*, causing branch canker disease on citrus and any crop, in Algeria. The *Botryosphaeriaceae* species were recovered from more than half of the trees sampled and were found in all the prospected orchards.

Lasiodiplodia was the most commonly isolated genus that was found in six of the ten surveyed orchards. This fact is consistent with previous studies, which showed that *Lasiodiplodia* species have the ability to target a wide variety of plants, distributed worldwide [40-42]. In fact, *Lasiodiplodia* species do not only occur as latent endophytes in asymptomatic plants but are also associated with different symptoms occurring on a variety of hosts including stem-end rot, fruit rot, decline, canker and dieback [41, 43-45]. In this study, *L. mediterranea* and *L. mitidjana* sp. nov. were the most frequently encountered species. *L. mediterranea* has been reported as the causal agent of canker and dieback of grapevine, holm oak as well as citrus, indicating its capability to target different hosts [28]. The latter findings lead Andolfi et al. [46] to isolate and characterize the main secondary metabolites produced by *L. mediterranea,* as well as to evaluate its phytotoxic and antifungal activities. According to the former authors [28], *L. mediterranea* has been found only on V-shaped necrotic sectors of grapevine while, it has been isolated from all the lesion types of citrus trees in this study. *L. mitidjana* sp. nov. was found in the five surveyed orchards. Isolates of this species were present predominantly in the wedge-shaped necrosis.

Dothiorella viticola was isolated at low frequency compared to *Lasiodiplodia* species found in this study. Interestingly, it was detected in six sampling sites. This species was first described as *Spencermartinisia viticola* by Phillips et al. [47]. It was obtained for the first time from *Vitis vinifera* in Spain. Recently, Yang et al. [38] regarded *Spencermartinsia* a synonym of *Dothiorella* and thus transferred the epithet *viticola* to *Dothiorella* as *Doth. viticola*. This taxonomic change was supported by a multi-gene phylogeny that included *Spencermartinsia* in *Dothiorella* genus [38, 48]. *Dothiorella viticola* has been reported from a wide range of woody hosts, including citrus trees [16, 21]. Recently, it has been also described as the causal agent of gummosis on citrus trees, in Tunisia [20]. According to Phillips et al. [18] and Dissanayake et al. [49], this species is known from China, Chile, USA, Spain, France, Australia, South Africa and Tunisia. Therefore, this study constitutes the first record of *Doth. viticola* in Algeria, which thus expands its known geographical range.

Two species of *Diplodia* genus, *D. seriata* and *D. mutila* were isolated from the surveyed orchards. *Diplodia* species are well known to cause damage on several economically important species and causing numerous disease symptoms including blight, dieback, rot diseases and canker [22, 28, 50-54]. In this study, *D. seriata* was frequently recovered from the sampling sites, which matches the findings of previous studies indicating the cosmopolitan nature of this species. This latter species is commonly reported as a pathogen on a large number of hosts and has been reported from hundreds of plant species [18, 49, 50]. *Diplodia mutila*, the second *Diplodia* species isolated in this study, was less frequently found in the prospected orchards. Moreover, to our knowledge, this is the first report of this species

in our country. In addition to Algeria, the USA is the only other country in which both *D. seriata* and *D. mutila* have been associated with citrus dieback [21]. These species have been found on apples in the USA [55, 56], Chile [57], France [58], Germany [59], Uruguay [60] and South Africa [61]; as well as in pear trees [60, 61], plum [62], peach and apricot [60, 63] and walnut [51].

All the *Botryosphaeriaceae* species of this study caused necrosis on the citrus shoots, with differences in the lengths of the lesions. These differences were observed between the species and also among isolates of the same species. Thus, the results suggest that *Diplodia seriata* and *L. mediterranea* could be considered as being the most aggressive, since they produced the longest lesion. However, *D. seriata* was significantly different compared to the rest of the isolates, which is consistent with previous studies that showed significant impact of *D. seriata* on several hosts, across the globe [28, 64-66].

For *L. mediterranea*, our results are in accordance with a previous study, which highlighted its aggressiveness in artificial inoculation experiments [28]. The least aggressive species of this case were *D. mutila* and *Doth. viticola*, producing the smallest lesion size. Nevertheless, these findings contradict previous study in which *D. mutila* was found to be the most aggressive based on lesion length [60]. According to Linaldeddu et al. [28] and Chakusary et al. [50], these differences in aggressiveness maybe due to several factors including genetic variability of isolates, age, type of host tissue, differences in susceptibility as well as inoculation methods and experimental conditions. In this case, extensive sampling from citrus as well as other hosts are required to further emphasise the findings and draw a final solid conclusion.

Overall, almost all the *Botryosphaeriaceae* species we identified have previously been detected on citrus trees with the exception of *L. mitidjana*, which was described for the first time associated with citrus dieback. Given the major impact of the *Botryosphaeriaceae* species isolated on declining trees, worldwide, it is important to emphasize the urgent need to implement prevention techniques and management strategies in order to minimize the incidence of these pathogens and to prevent their spread to new orchards. For a better understanding of citrus dieback, it is necessary to set up larger surveys that include all citrus production areas. These surveys would assess, more accurately, the impact of the trunk diseases pathogens and eventually identify the factors that influence the dieback. This will be set in order to identify a number of practices to prevent their development.

Acknowledgements

This work was supported by EFRR "Multidisciplinary research to increase application potential of nanomaterials in agricultural practice" (No. CZ.02.1.01/0.0/0.0/16_025/0007314). A. Alves acknowledges the financial support from FCT/MCTES to CESAM (UID/AMB/50017/2019), through national funds.

Author Contributions

Conceptualization: Akila Berraf-Tebbal, Artur Alves.

Data curation: Akila Berraf-Tebbal, Alla Eddine Mahamedi.

Formal analysis: Akila Berraf-Tebbal, Artur Alves.

Investigation: Akila Berraf-Tebbal, Wassila Aigoun-Mouhous, Milan Špetík, Aleš Eichmeier.

Methodology: Akila Berraf-Tebbal, Artur Alves, Alla Eddine Mahamedi.

Project administration: Akila Berraf-Tebbal, Jana Čechová, Robert Pokluda, Miroslav Baránek, Aleš Eichmeier.

Supervision: Akila Berraf-Tebbal, Artur Alves.

Writing – original draft: Akila Berraf-Tebbal**,** Alla Eddine Mahamedi, Wassila Aigoun-Mouhous.

Writing – review & editing: Akila Berraf-Tebbal, Artur Alves.

References

1. MADR. National agricultural statistics 2015. Algiers: Ministry of Agriculture and Rural Development; 2015.

2. Khanchouch K, Pane A, Chriki A, Cacciola SO. Major and emerging fungal diseases of *Citrus* in the Mediterranean region. Citrus Pathology. 2017:1. https://doi.org/10.5772/66943.

3. Bové JM. Virus and virus-like diseases of citrus in the Near East region. Rome: FAO; 1995.

4. Cohen M. Diagnosis of young tree decline, blight and sand hill decline of citrus by measurement of water uptake using gravity injection. Plant Disease Reporter. 1974;58(9):801-5.

5. Brlansky R, Lee R, Collins M. Structural comparison of xylem occlusions in the trunks of *Citrus* trees with blight and other decline diseases. Phytopathology. 1985;75(2):145-50. https://doi.org/10.1094/Phyto-75-145.

6. Elena K, Fischer M, Dimou D, Dimou DM. *Fomitiporia mediterranea* infecting citrus trees in Greece. Phytopathologia Mediterranea. 2006;45(1):35-9. https://doi.org/10.14601/Phytopathol_Mediterr-1813.

7. Polizzi G, Aiello D, Vitale A, Giuffrida F, Groenewald J, Crous P. First report of shoot blight, canker, and gummosis caused by *Neoscytalidium dimidiatum* on citrus in Italy. Plant Disease. 2009;93(11):1215-. https://doi.org/10.1094/PDIS-93-11-1215A. PMID: 30754593

8. Huang F, Hou X, Dewdney MM, Fu Y, Chen G, Hyde KD, Li H. *Diaporthe* species occurring on citrus in China. Fungal Diversity. 2013;61(1):237-50. https://doi.org/10.1007/s13225-013-0245-6.

9. Timmer L, Solel Z, Orozco-Santos M. *Alternaria* brown spot of mandarins. Compendium of citrus diseases. 2000;2:19-2000.

10. Udayanga D, Castlebury L, Rossman A, Hyde K. Species limits in *Diaporthe*: molecular re-assessment of *D. citri*, *D. cytosporella*, *D. foeniculina* and *D. rudis*. Persoonia: Molecular Phylogeny and Evolution of Fungi. 2014;32:83-101. https://doi.org/10.3767/003158514X679984. PMID: 25264384

11. Guarnaccia V, Groenewald JZ, Polizzi G, Crous PW. High species diversity in *Colletotrichum* associated with citrus diseases in Europe. Persoonia. 2017;39:32-50. https://doi.org/10.3767/persoonia.2017.39.02. PMID: 29503469

12. Polizzi G, editor Magnano di San Lio G., Catara A., 1992. Dry root rot of citranges in Italy. Proceedings of the International Society of Citriculture VII International Citrus Congress, Acireale 1992; 1992.

13. Hannachi I, Rezgui S, Cherif M. First report of mature citrus trees being affected by *Fusarium* wilt in Tunisia. Plant Disease. 2014;98(4):566. https://doi.org/10.1094/PDIS-12-12-1134-PDN. PMID: 30708695

14. Sandoval-Denis M, Guarnaccia V, Polizzi G, Crous P. Symptomatic Citrus trees reveal a new pathogenic lineage in *Fusarium* and two new *Neocosmospora* species. Persoonia: Molecular Phylogeny and Evolution of Fungi. 2018;40:1-25. https://doi.org/10.3767/persoonia.2018.40.01. PMID: 30504994

15. Mayorquin JS, Wang DH, Twizeyimana M, Eskalen A. Identification, distribution, and pathogenicity of *Diatrypaceae* and *Botryosphaeriaceae* associated with Citrus branch canker in the southern California desert. Plant Disease. 2016;100(12):2402-13. https://doi.org/10.1094/PDIS-03-16-0362-RE. PMID: 30686172

16. Adesemoye AO, Eskalen A. First Report of *Spencermartinsia viticola*, *Neofusicoccum australe*, and *N. parvum* Causing Branch Canker of Citrus in California. Plant Dis. 2011;95(6):770. https://doi.org/10.1094/PDIS-02-11-0092. PMID: 30731919

17. Trouillas FP, Pitt WM, Sosnowski MR, Huang R, Peduto F, Loschiavo A, Savocchia S, Scott ES, Gubler WD. Taxonomy and DNA phylogeny of *Diatrypaceae* associated with *Vitis vinifera* and other woody plants in Australia. Fungal Diversity. 2011;49(1):203-23. https://doi.org/10.1007/s13225-011-0094-0.

18. Phillips AJL, Alves A, Abdollahzadeh J, Slippers B, Wingfield MJ, Groenewald JZ, Crous PW. The *Botryosphaeriaceae*: genera and species known from culture. Stud Mycol. 2013;76(1):51-167. https://doi.org/10.3114/sim0021. PMID: 24302790

19. Burgess TI, Tan YP, Garnas J, Edwards J, Scarlett KA, Shuttleworth LA, Daniel R, Dann EK, Parkinson LE, Dinh Q. Current status of the Botryosphaeriaceae in Australia. Australasian Plant Pathology. 2018;48(1):35-44. https://doi.org/10.1007/s13313-018-0577-5.

20. Hamrouni N, Nouri M, Trouillas F, Said A, Sadfi-Zouaoui N, Hajlaoui M. *Dothiorella* gummosis caused by *Dothiorella viticola*, first record from citrus in Tunisia. New Disease Reports. 2018;38:10-. https://doi.org/10.5197/j.2044-0588.2018.038.010.

21. Adesemoye AO, Mayorquin JS, Wang DH, Twizeyimana M, Lynch SC, Eskalen A. Identification of Species of *Botryosphaeriaceae* Causing Bot Gummosis in Citrus in California. Plant Dis. 2014;98(1):55-61. https://doi.org/10.1094/PDIS-05-13-0492-RE. PMID: 30708572

22. Abdollahzadeh J, Javadi A, Mohammadi Goltapeh E, Zare R, Phillips AJ. Phylogeny and morphology of four new species of *Lasiodiplodia* from Iran. Persoonia. 2010;25:1-10. https://doi.org/10.3767/003158510X524150. PMID : 21339962

23. Berraf A, Péros J-P. Importance of Eutypa dieback and esca in Algeria and structure of the associated fungal community. OENO One. 2005;39(3):121-8. https://doi.org/10.20870/oeno-one.2005.39.3.896.

24. Ammad F, Benchabane M, Toumi M, Belkacem N, Guesmi A, Ameur C, Lecomte P, Merah O. Occurrence of *Botryosphaeriaceae* species associated with grapevine dieback in Algeria. Turkish Journal of Agriculture and Forestry. 2014;38(6):865-76. https://doi.org/10.3906/tar-1404-15.

25. Berraf-Tebbal A, Guereiro MA, Phillips AJ. Phylogeny of *Neofusicoccum* species associated with grapevine trunk diseases in Algeria, with description of *Neofusicoccum algeriense* sp. nov. Phytopathologia Mediterranea. 2014;53(3):416-27. https://doi.org/14385/14273.

26. Smahi H, Belhoucine-Guezouli L, Berraf-Tebbal A, Chouih S, Arkam M, Franceschini A, Linaldeddu B, Phillips A. Molecular characterization and pathogenicity of *Diplodia corticola* and other *Botryosphaeriaceae* species associated with canker and dieback of *Quercus* suber in Algeria. Mycosphere. 2017;8(2):1261-72. https://doi.org/10.5943/mycosphere/8/2/10.

27. Azouaoui-Idjer G, Della Rocca G, Pecchioli A, Bouznad Z, Danti R. First report of *Botryosphaeria iberica* associated with dieback and tree mortality of Monterey cypress (*Cupressus macrocarpa*) in Algeria. Plant Disease. 2012;96(7):1073-. https://doi.org/10.1094/PDIS-10-11-0901-PDN. PMID: 30727248

28. Linaldeddu BT, Deidda A, Scanu B, Franceschini A, Serra S, Berraf-Tebbal A, Boutiti MZ, Jamâa MB, Phillips A. Diversity of *Botryosphaeriaceae* species associated with grapevine and other woody hosts in Italy, Algeria and Tunisia, with descriptions of *Lasiodiplodia exigua* and *Lasiodiplodia mediterranea* sp. nov. Fungal Diversity. 2014;71(1):201-14. https://doi.org/10.1007/s13225-014-0301-x.

29. Santos J, Phillips A. Resolving the complex of *Diaporthe* (*Phomopsis*) species occurring on *Foeniculum vulgare* in Portugal. Fungal Diversity. 2009;34(11):111-25.

30. Alves A, Correia A, Luque J, Phillips A. *Botryosphaeria corticola*, sp. nov. on *Quercus* species, with notes and description of *Botryosphaeria stevensii* and its anamorph, *Diplodia mutila*. Mycologia. 2004;96(3):598-613. PMID: 21148880

31. White TJ, Bruns T, Lee S, Taylor J. Amplification and direct sequencing of fungal ribosomal RNA genes for phylogenetics. PCR protocols: a guide to methods and applications. 1990;18(1):315-22.

32. O'Donnell K, Cigelnik E, Nirenberg HI. Molecular systematics and phylogeography of the *Gibberella fujikuroi* species complex. Mycologia. 1998;90(3):465-93. https://doi.org/10.1080/00275514.1998.12026933.

33. Alves A, Crous PW, Correia A, Phillips A. Morphological and molecular data reveal cryptic speciation in *Lasiodiplodia theobromae*. Fungal Diversity. 2008;28:1-13. https://doi.org/10.2307/3762177.

34. Hall TA, editor BioEdit: a user-friendly biological sequence alignment editor and analysis program for Windows 95/98/NT. Nucleic acids symposium series; 1999: [London]: Information Retrieval Ltd., c1979-c2000.

35. Altschul SF, Gish W, Miller W, Myers EW, Lipman DJ. Basic local alignment search tool. J Mol Biol. 1990;215(3):403-10. https://doi.org/10.1016/S0022-2836(05)80360-2.

36. Thompson JD, Gibson TJ, Plewniak F, Jeanmougin F, Higgins DG. The CLUSTAL_X windows interface: flexible strategies for multiple sequence alignment aided by quality analysis tools. Nucleic acids research. 1997;25(24):4876-82. https://doi.org/10.1093/nar/25.24.4876. PMID: 9396791

37. Kumar S, Stecher G, Li M, Knyaz C, Tamura K. MEGA X: Molecular Evolutionary Genetics Analysis across Computing Platforms. Mol Biol Evol. 2018;35(6):1547-9. https://doi.org/10.1093/molbev/msy096. PMID: 29722887

38. Yang T, Groenewald JZ, Cheewangkoon R, Jami F, Abdollahzadeh J, Lombard L, Crous PW. Families, genera, and species of Botryosphaeriales. Fungal biology. 2017;121(4):322-46. https://doi.org/10.1016/j.funbio.2016.11.001. PMID: 28317538

39. Raimondo F, Nardini A, Salleo S, Cacciola SO, Gullo MAL. A tracheomycosis as a tool for studying the impact of stem xylem dysfunction on leaf water status and gas exchange in *Citrus aurantium* L. Trees. 2010;24(2):327-33. https://doi.org/10.1007/s00468-009-0402-4.

40. Slippers B, Johnson GI, Crous PW, Coutinho TA, Wingfield BD, Wingfield MJ. Phylogenetic and morphological re-evaluation of the *Botryosphaeria* species causing diseases of *Mangifera indica*. Mycologia. 2005;97(1):99-110. https://doi.org/10.3852/mycologia.97.1.99. PMID: 16389961

41. Sakalidis ML, Ray JD, Lanoiselet V, Hardy GES, Burgess TI. Pathogenic *Botryosphaeriaceae* associated with Mangifera indica in the Kimberley region of Western Australia. European journal of plant pathology. 2011;130(3):379-91. https://doi.org/10.1007/s10658-011-9760-z.

42. Rodríguez-Gálvez E, Guerrero P, Barradas C, Crous PW, Alves A. Phylogeny and pathogenicity of *Lasiodiplodia* species associated with dieback of mango in Peru. Fungal biology. 2017;121(4):452-65. https://doi.org/10.1016/j.funbio.2016.06.004. PMID: 28317545

43. Ismail A, Cirvilleri G, Polizzi G, Crous P, Groenewald J, Lombard L. *Lasiodiplodia* species associated with dieback disease of mango (*Mangifera indica*) in Egypt. Australasian Plant Pathology. 2012;41(6):649-60. https://doi.org/10.1007/s13313-012-0163-1.

44. Marques MW, Lima NB, de Morais MA, Barbosa MAG, Souza BO, Michereff SJ, Phillips AJ, Câmara MP. Species of *Lasiodiplodia* associated with mango in Brazil. Fungal Diversity. 2013;61(1):181-93. https://doi.org/10.1007/s13225-013-0231-z.

45. Trakunyingcharoen T, Lombard L, Groenewald JZ, Cheewangkoon R, To-Anun C, Crous P. Caulicolous Botryosphaeriales from Thailand. Persoonia: Molecular Phylogeny and Evolution of Fungi. 2015;34:87–99. https://doi.org/10.3767/003158515X685841. PMID: 26240447

46. Andolfi A, Basso S, Giambra S, Conigliaro G, Lo Piccolo S, Alves A, Burruano S. Lasiolactols A and B produced by the grapevine fungal pathogen *Lasiodiplodia mediterranea*. Chemistry & biodiversity. 2016;13(4):395-402. https://doi.org/10.1002/cbdv.201500104. PMID: 26938016

47. Phillips A, Alves A, Pennycook S, Johnston P, Ramaley A, Akulov A, Crous P. Resolving the phylogenetic and taxonomic status of dark-spored teleomorph genera in the *Botryosphaeriaceae*. Persoonia: Molecular Phylogeny and Evolution of Fungi. 2008;21:29-55. https://doi.org/10.3767/003158508X340742. PMID: 20396576

48. Slippers B, Boissin E, Phillips A, Groenewald JZ, Lombard L, Wingfield MJ, Postma A, Burgess T, Crous PW. Phylogenetic lineages in the Botryosphaeriales: a systematic and evolutionary framework. Studies in Mycology. 2013;76(1):31-49. https://doi.org/10.3114/sim0020. PMID: 24302789

49. Dissanayake A, Phillips A, Li X, Hyde K. *Botryosphaeriaceae*: current status of genera and species. Mycosphere. 2016;7(7):1001-73. https://doi.org/10.5943/mycosphere/si/1b/13.

50. Chakusary MK, Mohammadi H, Khodaparast SA. Diversity and pathogenicity of *Botryosphaeriaceae* species on forest trees in the north of Iran. European Journal of Forest Research. 2019:1-20. https://doi.org/10.1007/s10342-019-01200-7.

51. Chen S, Morgan DP, Hasey JK, Anderson K, Michailides TJ. Phylogeny, morphology, distribution, and pathogenicity of *Botryosphaeriaceae* and *Diaporthaceae* from English Walnut in California. Plant Dis. 2014;98(5):636-52. https://doi.org/10.1094/PDIS-07-13-0706-RE. PMID: 30708543

52. Lazzizera C, Frisullo S, Alves A, Lopes J, Phillips A. Phylogeny and morphology of *Diplodia* species on olives in southern Italy and description of *Diplodia olivarum* sp. nov. Fungal Divers. 2008;31:63-71.

53. Phillips AJ, Crous PW, Alves A. *Diplodia seriata*, the anamorph of “*Botryosphaeria*” *obtusa*. Fungal Diversity. 2007;25:141-55.

54. Úrbez-Torres J, Peduto F, Vossen P, Krueger W, Gubler W. Olive twig and branch dieback: etiology, incidence, and distribution in California. Plant Disease. 2013;97(2):231-44. https://doi.org/10.1094/PDIS-04-12-0390-RE. PMID: 30722318

55. Crespo M, Moral J, Michailides T, Trouillas F. First report of black rot on apple fruit caused by *Diplodia seriata* in California. Plant Disease. 2018;102(4):824. https://doi.org/10.1094/PDIS-07-17-1023-PDN.

56. Kim Y, Kwak J, Aguilar C, Xiao C. First report of black rot on apple fruit caused by *Diplodia seriata* in Washington State. Plant Disease. 2016;100(7):1499-. https://doi.org/10.1094/PDIS-12-15-1463-PDN.

57. Díaz GA, Latorre BA, Ferrada E, Lolas M. Identification and characterization of *Diplodia mutila*, *D. seriata*, *Phacidiopycnis washingtonensis* and *Phacidium lacerum* obtained from apple (*Malusx domestica*) fruit rot in Maule region, Chile. European journal of plant pathology. 2019;153(1):211-25. https://doi.org/10.1007/s10658-018-01640-8.

58. Giraud M. Apple Black Rot. Infos-Ctifl. 2009(257):36-41.

59. Weber R, Dralle N. Fungi associated with blossom-end rot of apples in Germany. Eur J Hort Sci. 2013;78:97-105.

60. Sessa L, Abreo E, Bettucci L, Lupo S. *Botryosphaeriaceae* species associated with wood diseases of stone and pome fruits trees: symptoms and virulence across different hosts in Uruguay. European journal of plant pathology. 2016;146(3):519-30. https://doi.org/10.1007/s10658-016-0936-4.

61. Cloete M, Fourie PH, Damm U, Crous PW, Mostert L. Fungi associated with dieback symptoms of apple and pear trees, a possible inoculum source of grapevine trunk disease pathogens. Phytopathologia Mediterranea. 2011;50:S176-S90. https://doi.org/10.14601/Phytopathol_Mediterr-9004.

62. Damm U, Crous PW, Fourie PH. *Botryosphaeriaceae* as potential pathogens of Prunus species in South Africa, with descriptions of *Diplodia africana* and *Lasiodiplodia plurivora* sp. nov. Mycologia. 2007;99(5):664-80. https://doi.org/10.3852/mycologia.99.5.664. PMID: 18268901

63. Laundon G. *Botryosphaeria obtusa*, *B. stevensii*, and *Otthia spiraeae* in New Zealand. Transactions of the British Mycological Society. 1973;61(2):369-IN17. https://doi.org/10.1016/S0007-1536(73)80158-5.

64. Larignon P. Maladies cryptogamiques du bois de la vigne: symptomatologie et agents pathogènes. Institut Français de la Vigne et du Vin, Grau du Roi dans le Gard. 2012

65. Luque J, Martos S, Aroca A, Raposo R, Garcia-Figueres F. Symptoms and fungi associated with declining mature grapevine plants in northeast Spain. Journal of Plant Pathology. 2009:381-90.

66. Morales A, Latorre BA, Piontelli E, Besoain X. *Botryosphaeriaceae* species affecting table grape vineyards in Chile and cultivar susceptibility. Ciencia e investigación agraria. 2012;39(3):445-58. https://doi.org/10.4067/S0718-16202012000300005.

Supplementary table S1. Details of strains included in the phylogenetic and/or morphological analyses.

**Species**	**Culture collection number(s)**[1]	**Substrate**	**Country**	**Collector(s)**	**GenBank accession numbers**	
					**ITS**	***tef1***
L. avicenniae	CMW 41467	*Avicennia*, asymptomatic branches	South Africa	J. A. Osorio & J. Roux	KP860835	KP860680
L. avicenniae	LAS199	*Avicennia*, asymptomatic branches	South Africa	J. A. Osorio & J. Roux	KU587957	KU587947
L. brasiliense	CMM 4015, ex-type	*Mangifera*, stems	Brazil	M. W. Marques	JX464063	JX464049
L. brasiliense	CMM 4469	*Anacardium*	Brazil	-	KT325574	KT325580
L. bruguierae	CMW 41470	*Bruguiera*¸ asymptomatic branches	South Africa	J. A. Osorio & J. Roux	KP860833	KP860678
L. bruguierae	CMW 42480	*Bruguiera*, asymptomatic branches	South Africa	J. A. Osorio & J. Roux	KP860832	KP860677
L. caatinguensis	CMM 1325	*Citrus*	Brazil	I. B. L. Coutinho & J. S. Lima	KT154760	KT008006
L. caatinguensis	IBL 381	*Spondias*	Brazil	J. S. Lima & J. E. Cardoso	KT154757	KT154751
L. chinensis	CGMCC 3.18061	*Unknown*, branch	China	W. He & Z. P. Dou	KX499889	KX499927
L. chinensis	CGMCC 3.18044	*Vaccinium*, branch	China	J. H. Zhao	KX499875	KX499913
L. cinnamomi	CFCC 51997	*Cinnamomum*, branch	China	N. Jiang	MG866028	MH236799
L. cinnamomi	CFCC 51998	*Cinnamomum*, branch	China	N. Jiang	MG866029	MH236800

L. citricola	CBS 124706	*Citrus* sp., twigs	Iran	A. Shekari	GU945353	GU945339
L. citricola	CBS 124707, ex-type	*Citrus*, twigs	Iran	J. Abdollahzadeh & A. Javadi	GU945354	GU945340
L. crassispora	CMW 13488	*Eucalyptus*, wood	Venezuela	S. Mohali	DQ103552	DQ103559
L. crassispora	CBS 118741, ex-type	*Santalum*	Australia	T. I. Burgess & B. Dell	DQ103550	EU673303
L. euphorbicola	CMW 33350	*Adansonia*	Botswana	-	KU887149	KU887026
L. euphorbicola	CMW 36231	*Adansonia*	Zimbabwe	-	KU887187	KU887063
L. exigua	BL 184	*Retama*, branch canker	Tunisia	B. T. Linaldeddu	KJ638318	KJ638337
L. exigua	CBS 137785, ex-type	*Retama*, branch canker	Tunisia	B. T. Linaldeddu	KJ638317	KJ638336
L. gilanensis	CBS 124704, ex-type	*Citrus*, fallen twigs	Iran	J. Abdollahzadeh & A. Javadi	GU945351	GU945342
L. gilanensis	CBS 124705	*Citrus* sp., fallen twigs	Iran	J. Abdollahzadeh & A. Javadi	GU945352	GU945341
L. gonubiensis	CMW 14077, ex-type	*Syzygium*	South Africa	D. Pavlic	AY639595	DQ103566
L. gonubiensis	CMW 14078, ex-paratype	*Syzigium*	South Africa	D. Pavlic	AY639594	DQ103567
L. gravistriata	CMM 4564	*Anacardium*, stems	Brazil	M. S. B. Netto	KT250949	KT250950
L. gravistriata	CMM 4565	*Anacardium*, stems	Brazil	M. S. B. Netto	KT250947	KT266812
L. hormozganensis	CBS 124708	*Mangifera*, twigs	Iran	J. Abdollahzadeh & A. Javadi	GU945356	GU945344

L. hormozganensis	CBS 124709, ex-type	*Olea*, twigs	Iran	J. Abdollahzadeh & A. Javadi	GU945355	GU945343
L. hyalina	CGMCC 3.17975	*Acacia*, cankered stems	China	Y. Zhang & Y. P. Zhou	KX499879	KX499917
L. hyalina	CGMCC 3.18383	unidentified woody plant, cankered branches	China	Z. P. Dou & Z. C. Liu	KY767661	KY751302
L. iraniensis	IRAN 1520C, ex-type	*Salvadora*, twigs	Iran	J. Abdollahzadeh & A. Javadi	GU945346	GU945334
L. iraniensis	IRAN 1502C	*Juglans*, twigs	Iran	A. Javadi	GU945347	GU945335
L. laeliocattleyae	CBS 167.28	*Laeliocattleya*, leaves	Italy	C. Sibilia	KU507487	KU507454
L. laeliocattleyae	CBS 130992	*Mangifera*, leaves	Egypt	A. M. Ismail	JN814397	JN814424
L. lignicola	CBS 134112	Wood	Thailand	-	JX646797	KU887003
L. macrospora	CMM 3833, ex-type	*Jatropha*, collar and root rot	Brazil	A. R. Machado & O. L. Pereira	KF234557	KF226718
L. mahajangana	CMW 27801, ex-type	*Terminalia*, healthy branches	Madagascar	J. Roux	FJ900595	FJ900641
L. mahajangana	CMW 27820	*Terminalia*, healthy branches	Madagascar	J. Roux	FJ900597	FJ900643
L. margaritacea	CBS 122519, ex-type	*Adansonia*, dying twigs	Australia	T. I. Burgess & M. J. Wingfield	EU144050	EU144065
L. margaritacea	CBS 122065	*Adansonia gibbosa*, dying twigs	Australia	T. I. Burgess	EU144051	EU144066
L. mediterranea	CBS 137783, ex-type	*Quercus*, branch canker	Italy	B. T. Linaldeddu	KJ638312	KJ638331
L. mediterranea	CBS 137784	*Vitis*, brown stripe under the bark	Italy	S. Serra	KJ170150	KJ170151

L. missouriana	CBS 128311, ex-type	Wedge-shape canker of grapevine cv. Catawba (complex hybrid of North America *Vitis* species)	USA	K. Striegler & G. M. Leavitt	HQ288225	HQ288267
L. missouriana	CBS 128312	Wedge-shape canker of grapevine cv. Catawba (complex hybrid of North America *Vitis* species)	USA	K. Striegler & G. M. Leavitt	HQ288226	HQ288268
L. parva	CBS 456.78, ex-type	Cassava-field soil	Colombia	O. Rangel	EF622083	EF622063
L. parva	CBS 494.78	Cassava-field soil	Colombia	O. Rangel	EF622084	EF622064
L. plurivora	STE-U 5803, ex-type	*Prunus*, wood canker	South Africa	U. Damm	EF445362	EF445395
L. plurivora	STE-U 4583	*Vitis*, symptomatic	South Africa	F. Halleen	AY343482	EF445396
L. pontae	CMM 1277	*Spondias*, necrotic canker	Brazil	J.S. Lima & F.C.O. Freire	KT151794	KT151791
L. pseudotheobromae	CBS 116459, ex-type	*Gmelina*, twigs	Costa Rica	J. Carranza- Velazquez	EF622077	EF622057
L. pseudotheobromae	CGMCC 3.18047	*Pteridium*, twigs	China	-	KX499876	KX499914
L. pyriformis	CBS 121770, ex-type	*Pinus*, fruiting structures	Namibia	F. J. J. van der Walt & J. Roux	EU101307	EU101352
L. pyriformis	CBS 121771	*Pinus*, fruiting structures	Namibia	F. J. J. van der Walt & J. Roux	EU101308	EU101353
L. rubropurpurea	WAC 12535, ex-type	*Eucalyptus*, canker	Australia	T. I. Burgess	DQ103553	EU673304

L. rubropurpurea	WAC 12536	*Eucalyptus*, canker	Australia	T. I. Burgess	DQ103554	DQ103572
L. sterculiae	CBS 342.78, ex-type	*Sterculia*	Germany	S. Bruhn	KX464140	KX464634
L. subglobosa	CMM 3872, ex-type	*Jatropha*, collar and root rot	Brazil	A. R. Machado & O. L. Pereira	KF234558	KF226721
L. subglobosa	CMM 4046	*Jatropha*	Brazil	A. R. Machado & O. L. Pereira	KF234560	KF226723
L. thailandica	CGMCC 3.18382	*Podocarpus*, cankered branch	China	D. Zhipeng & L. Zuchen	KY767662	KY751303
L. thailandica	CGMCC 3.18384	*Albizia*, cankered branch	China	Z.P. Dou & Z.C. Liu	KY767663	KY751304
L. theobromae	CBS 164.96, ex-neotype	Fruit along coral reef coast	Papua New Guinea	A. Aptroot	AY640255	AY640258
L. theobromae	CBS 111530	*Leucospermum*	USA	J. E. Taylor	EF622074	EF622054
L. venezuelensis	WAC 12539, ex-type	*Acacia*, wood	Venezuela	S. Mohali	DQ103547	EU673305
L. venezuelensis	WAC 12540	*Acacia*, wood	Venezuela	S. Mohali	DQ103548	DQ103569
L. viticola	CBS 128313, ex-type	Wedge-shape canker of grapevine cv. Vignoles (complex hybrid of North America *Vitis* species)	USA	R. D. Cartwright & W. D. Gubler	HQ288227	HQ288269
L. viticola	UCD 2604MO	*Vitis*	USA	J. R. Urbez-Torres	HQ288228	HQ288270
L. vitis	CBS 124060, ex-type	*Vitis*, wood fragment	Italy, Sicily	S. Burruano	KX464148	KX464642
D seriata	CBS 112555	*Vitis*, dead stems	Portugal	A. J. L. Phillips	AY259094	AY573220

D mutila	CBS 112553	*Vitis*	Portugal	A. Alves	AY259093	AY573219

[1] BL: Personal number of B.T. Linaldeddu; CBS: CBS-KNAW Fungal Biodiversity Centre, Utrecht, The Netherlands; CFCC: China Forestry Culture Collection Center, Beijing, China; CMM: Culture Collection of Phytopathogenic Fungi “Prof. Maria Menezes”, Universidade Federal Rural de Pernambuco, Recife, Brazil; CMW: Tree Patholgy Co-operative Program, Forestry and Agricultural Biotechnology Institute, University of Pretoria, South Africa; IRAN: Iranian Fungal Culture Collection, Iranian Research Institute of Plant Protection, Iran; WAC: Department of Agriculture, Western Australia Plant Pathogen Collection, South Perth, Western Australia.

Supplementary figure S1.

Maximum Parsimony phylogenetic tree resulting from the analysis of the combined ITS and *tef1*-α sequence data from *Lasiodiplodia* species. The tree was rooted to *Diplodia mutila* and *Diplodia seriata*.

 Click here to access/download;Figure;Fig 1.tif

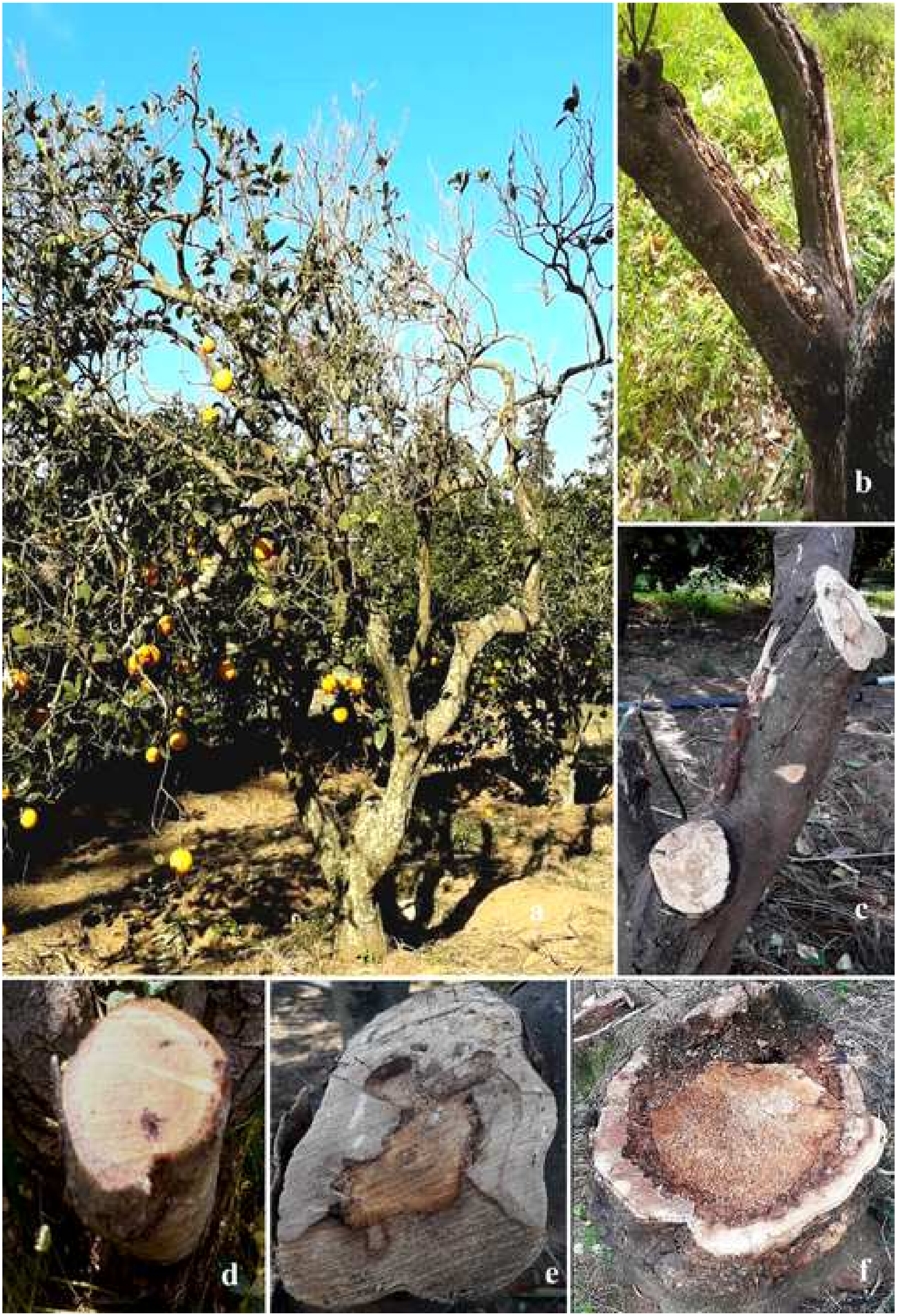

Click here to access/download;Figure;Fig 2.tif

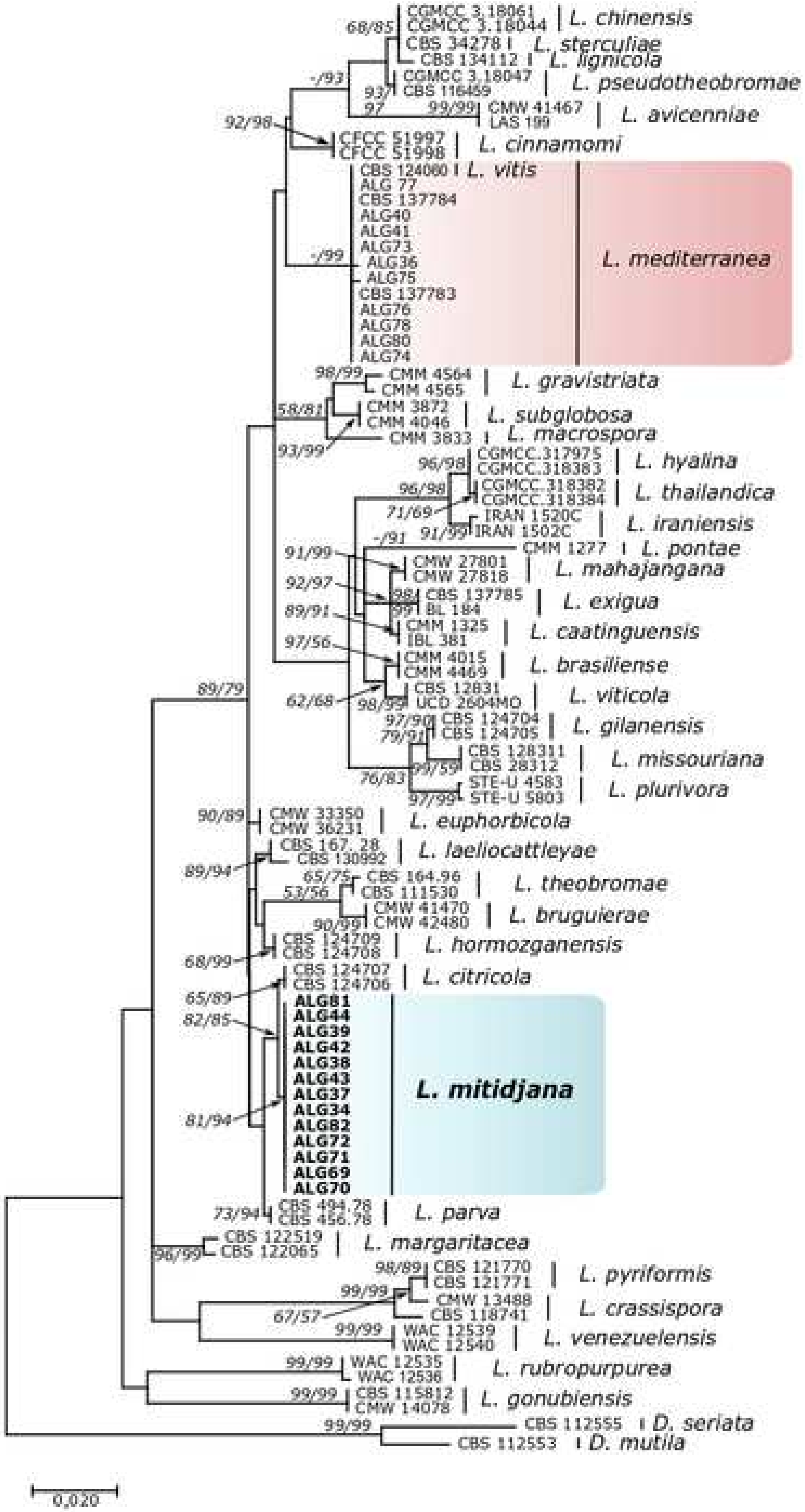

 Click here to access/download;Figure;Fig 3.tif

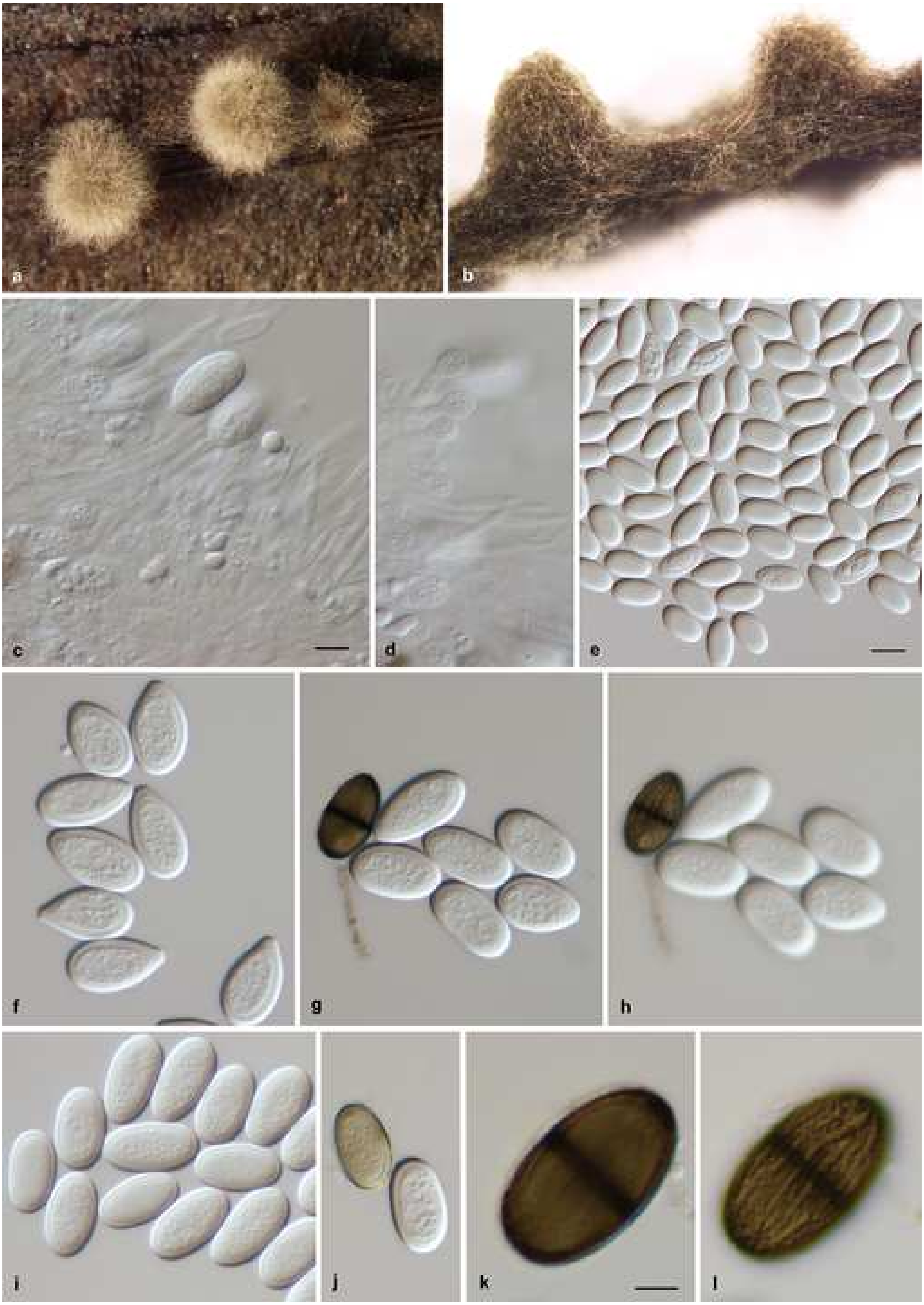

Click here to access/download
Supporting Information
Fig S1.tif

Lasiodiplodia mitidjana sp. nov. and other *Botryosphaeriaceae* species causing branch canker and dieback of *Citrus sinensis* in Algeria

Akila Berraf-Tebbal[1*], Alla Eddine Mahamedi[2,3], Wassila Aigoun-Mouhous[2,4], Milan Špetík[1], Jana Čechová[1], Robert Pokluda[1], Miroslav Baránek[1], Aleš Eichmeier[1], Artur Alves[5]

[1] Mendel University in Brno, Faculty of Horticulture, Mendeleum - Institute of Genetics, Valticka, Lednice, Czech Republic

[2] Laboratoire de Biologie des Systèmes Microbiens (LBSM), Département des Sciences Naturelles, Ecole Normale Supérieure de Kouba-Alger, Alger, Algeria

[3] Département de Biologie, Faculté des Sciences de la Nature et de la Vie et Sciences de la Terre, Université de Ghardaïa, Ghardaïa, Algeria

[4] Département des Biotechnologies, Faculté des Sciences de la Nature et de la Vie, Université de Blida, Blida, Algeria

[5] Departamento de Biologia, CESAM, Universidade de Aveiro, Aveiro, Portugal

* Corresponding author
E-mail: qqberraf@mendelu.cz

Abstract

Several *Botryosphaeriaceae* species are known to occur worldwide, causing dieback, canker and fruit rot on various hosts. Surveys conducted in ten commercial citrus orchards in the northern region of Algeria revealed five species of *Botryosphaeriaceae* belonging to three genera associated with diseased trees. Morphological and cultural characteristics as well as phylogenetic analyses of the internal transcribed spacer (ITS) region and the translation elongation factor 1-alpha (*tef1*-α) identified *Diplodia mutila*, *Diplodia seriata*, *Dothiorella viticola*, *Lasiodiplodia mediterranea* and a novel species which is here described as *Lasiodiplodia mithidjana* sp. nov.. Of these, *L. mithidjana* (14.1% of the samples) and *L. mediterranea* (13% of the samples) were the most widespread and abundant species. Pathogenicity tests revealed that *L. mediterranea* and *D. seriata* were the most aggressive species on citrus shoots. This study highlights the importance of *Botryosphaeriaceae* species as agents of canker and dieback of citrus trees in Algeria.

Key words: Citrus cultivation, trunk diseases, fungi, identification, taxonomy, pathogenicity.

Introduction

Citrus cultivation is one of the major contributors to Algerian wealth and is part of the traditional agriculture of the country. Many types of citrus are grown in Algeria, including oranges (48 400 ha), clementine (10 817 ha), mandarins (2 347 ha), lemons (4 409 ha) and grapefruits (83 ha) [1]. Despite the high adaptation capacity of citrus trees to different climates [2], a number of unfavourable factors has led to a decrease of the total citrus yield in Algeria. Among these factors, ageing trees, droughts, inappropriate cultural practices and the effects of various pests and pathogens are the most important [2, 3]. Citrus diseases are numerous and diverse, and are caused by phytopathogenic agents belonging to viruses, viroids, phytoplasmas, bacteria, and fungi [2] Some pathogens cause very serious diseases, predisposing to, and inciting dieback, while others are less serious [2-5].

Recently, trunk diseases have become a growing threat in both, old and newly established orchards of citrus, worldwide. Symptoms include leaves that become yellow and fall early, shoots and twigs die, increasing the risk of citrus decay as the damage expands to the trunk [2, 6-8]. To date, among the fungi that impact citrus, *Diaporthe* species are well known for causing stem-end rot and melanose of fruits, young leaf and shoot gummosis and blight of perennial branches and trunks, in Greece, Italy, Malta, Portugal, Spain, China, Korea, New Zealand, and the USA [8-11]. *Fusarium* and *Neocosmospora* have also been reported causing canker and dieback diseases of citrus, in Tunisia, Greece, Italy and Spain [12-14]. The *Diatrypaceae* are other canker and dieback pathogens impacting citrus orchards [15]. Several *Eutypella* spp. have been reported from *Citrus* sp. In southern California desert, three distinct species of *Eutypella* are found associated with citrus branch canker, namely: *Eutypella citricola*, *E. microtheca* and a *Eutypella* sp. [15-17].

In addition to the above fungal pathogens that compromise citrus crops, several *Botryosphaeriaceae* species are known to colonize citrus trees. The *Botryosphaeriaceae* family is recognized as an important and widely distributed plant pathogen, which impacts on a variety of economically important hosts. It comprises 24 genera encompassing 222 species, living as endophytes, saprobes, or plant pathogens [18, 19]. Recent studies carried out in California, Italy and Tunisia have highlighted the *Botryosphaeriaceae* as the most prevalent fungi that cause cankers, vascular necrosis and dieback of citrus trees [7, 15, 16, 20]. Adesemoye et al. [21] recovered various *Botryosphariaceae* species from necrotic tissues of citrus branch canker and rootstock, including, *Diplodia seriata*, *D. mutila*, *Dothiorella viticola*, *D. iberica*, *Lasiodiplodia parva*, *Neofusicoccum australe*, *N. luteum*, *N. mediterraneum*, *N. parvum* and *Neoscytalidium dimidiatum*. In Iran, Abdollahzadeh et al. [22], described *Lasiodiplodia citricola* from citrus trees showing symptoms of branch dieback.

In Algeria, members of the *Botryosphaeriaceae* have been reported to cause diseases on *Vitis vinifera* [23-25], *Quercus suber* [26] and *Cupressus macrocarpa* [27]. Linaldeddu et al. [28] isolated and described *Lasiodiplodia mediterranea* from a cankered branch of *Citrus sinensis* trees in northern Algeria. However, the impact of *Botryosphaeriaceae* species on citrus trees has not been studied in detail. Therefore, the aim of this study was to investigate

and determine the incidence of the *Botryosphaeriaceae* species associated with branch canker and dieback in the major citrus-growing region of Algeria.

Materials and Methods

Ethics Statement

No specific permits were required for the described field studies. This study did not involve endangered or protected species.

Field survey and sampling

Surveys were conducted in ten commercial orchards in the northern region of Algeria. Specifically, in the Mitidja plain at the base of the Tell Atlas Mountains. The sampling was done in four municipalities; namely Oued El Aleug (4 orchards), Chiffa (2 orchards), Boufarik (2 orchards) and the coastal town, Sidi Fredj, located within the territory of the Staoueli municipality, situated by the Mediterranean Sea (2 orchards). The field diagnosis and sampling were performed between April 2013 and March 2015. Samples were collected from the orchards with permission of landowners. Trunks and branches showing symptoms such as dead shoots, defoliation, cankers, wood necrosis, and dieback were collected, randomly. A total of 80 symptomatic sweet orange (*Citrus sinensis*) trees were sampled (Table 1).

Table 1. Citrus orchards surveyed and number of samples collected.

Locality	GPS coordinates	Orchards	Area (ha)	Number of trees sampled	Number of samples processed
Oued El Alleug	36°33'21"N 2°47'22"E	a	18	5	9
		b	16	5	6
		c	28	5	7
		d	6.8	5	6
Chiffa	36°27'44"N 2°44'27"E	a	25	10	13
		b	18	10	10
Boufarik	36°34'31"N 2°54'46"E	a	43	10	10
		b	27	10	11
Staoueli	36°45'12"N 2°53'17"E	a	32	10	10
		b	15	10	10

Fungal isolation and morphological characterization

In the laboratory, all samples were processed by peeling the outer bark surface with a sterilized scalpel. Longitudinal and transversal cuts were made to reveal the type and localization of the internal necrosis. From each lesion detected, ten pieces of wood, approx. 5 mm^2, were cut from the margins between necrotic and healthy tissues. These pieces were submerged in 4 % sodium hypochlorite for 15 min, washed thrice with sterile distilled water, dried with sterilized filter paper and placed onto the surface of potato dextrose agar (PDA, Difco Laboratories). Plates were incubated at 25 °C until growth was detected. The mycelium emerging from wood pieces were transferred onto fresh PDA plates and incubated under the same conditions.

Preliminary identifications to genus and tentative species level were based on colony and conidial morphology (colony colour, colony growth pattern, conidial size, shape, colour, striation, septation, conidiogenous cells, and presence of paraphyses) according to Phillips et al. [18]. Isolates that lacked pycnidia production on PDA were placed on autoclaved pine needles in ¼ strength PDA within 2–3 weeks, incubated at 25 °C under mixed near-UV and cool-white fluorescent light in a 12 h light 12 h dark regime for 2–6 weeks, to enhance fruiting body production. Conidiogenous layer and conidia were mounted in 100 % lactic acid and observed with a Nikon 80i light microscope.

DNA extraction, PCR amplification and sequencing

Total genomic DNA was extracted from 7 days old axenic cultures, grown on PDA at 25 °C, following Santos and Phillips [29]. PCR reactions were carried out with *Taq* DNA polymerase, nucleotides and buffers supplied by MBI Fermentas (Vilnius, Lithuania). PCR reaction mixtures were prepared as previously described by Alves et al. [30], with the addition of 5 % DMSO to improve the amplification of some difficult DNA templates. The ITS region plus D1/D2 domain of the LSU was amplified with the primer pair ITS1 [31] and NL4 [32]. The amplification conditions were initial denaturation of 5 min at 95 °C, followed by 29 cycles of 30 s at 94 °C, 30 s at 50 °C, and 1.5 min at 72 °C, and a final extension of 10 min at 72 °C. Part of the translation elongation factor 1 alpha gene (*tef1*-α) was amplified with primers EF1-688F and EF1-1251R [33]. The amplification conditions were: initial denaturation of 5 min at 95 C, followed by 30 cycles of 30 s at 94 °C, 45 s at 55 °C, 1½ min at 72 °C, and a final extension period of 10 min at 72 °C. ITS and *tef1*-α regions were sequenced in both directions by STAB Vida Lda (Portugal), using the Sanger method.

The nucleotide sequences were read and edited with BioEdit Alignment Editor V.7.0.9.0 [34]. Newly generated sequences were deposited in GenBank (Table 2). Homological sequences of the newly sequenced ones were retrieved from the GenBank using the Basic Local Alignment Search Tool (BLAST) [35].

Table 2. ***Botryosphaeriaceae*** **species included in this study**

**Species**	**Isolate number**	**Host/ Substrate**	**Origin**	**Collector**	**GenBank accession numbers**	
					**ITS**	***tef1-α***
Lasiodiplodia mediterranea	ALG77	*Citrus*/wood canker	Algeria, Boufarik	Akila Berraf-Tebbal	MN104094	MN159093
L. mediterranea	ALG76	*Citrus*/wood canker	Algeria, Oued El Alleug	Akila Berraf-Tebbal	MN104095	MN159094
L. mediterranea	ALG40	*Citrus*/wood canker	Algeria, Oued El Alleug	Akila Berraf-Tebbal	MN104096	MN159095
L. mediterranea	ALG78	*Citrus*/wood canker	Algeria, Oued El Alleug	Akila Berraf-Tebbal	MN104097	MN159096
L. mediterranea	ALG41	*Citrus*/wood canker	Algeria, Oued El Alleug	Akila Berraf-Tebbal	MN104098	MN159097
L. mediterranea	ALG36	*Citrus*/wood canker	Algeria, Oued El Alleug	Akila Berraf-Tebbal	MN104099	MN159098
L. mediterranea	ALG80	*Citrus*/wood canker	Algeria, Oued El Alleug	Akila Berraf-Tebbal	MN104100	MN159099
L. mediterranea	ALG73	*Citrus*/wood canker	Algeria, Boufarik	Akila Berraf-Tebbal	MN104101	MN159100
L. mediterranea	ALG74	*Citrus*/wood canker	Algeria, Boufarik	Akila Berraf-Tebbal	MN104102	MN159101
L. mediterranea	ALG75	*Citrus*/wood canker	Algeria, Boufarik	Akila Berraf-Tebbal	MN104103	MN159102
L. mediterranea	CBS 124060	*Vitis*, wood fragment	Italy, Sicily	S. Burruano	KX464148	MN938928
L. mitidjana	ALG81	*Citrus*/wood canker	Algeria, Oued El Alleug	Akila Berraf-Tebbal	MN104104	MN159103
L. mitidjana	ALG44	*Citrus*/wood canker	Algeria, Oued El Alleug	Akila Berraf-Tebbal	MN104105	MN159104

L. mitidjana	ALG39	*Citrus*/wood canker	Algeria, Oued El Alleug	Akila Berraf-Tebbal	MN104106	MN159105
L. mitidjana	ALG42	*Citrus*/wood canker	Algeria, Oued El Alleug	Akila Berraf-Tebbal	MN104107	MN159106
L. mitidjana	ALG38	*Citrus*/wood canker	Algeria, Oued El Alleug	Akila Berraf-Tebbal	MN104108	MN159107
L. mitidjana	ALG43	*Citrus*/wood canker	Algeria, Oued El Alleug	Akila Berraf-Tebbal	MN104109	MN159108
L. mitidjana	ALG37	*Citrus*/wood canker	Algeria, Oued El Alleug	Akila Berraf-Tebbal	MN104110	MN159109
L. mitidjana	ALG34	*Citrus*/wood canker	Algeria, Oued El Alleug	Akila Berraf-Tebbal	MN104111	MN159110
L. mitidjana	ALG82	*Citrus*/wood canker	Algeria, Oued El Alleug	Akila Berraf-Tebbal	MN104112	MN159111
L. mitidjana	ALG72	*Citrus*/wood canker	Algeria, Boufarik	Akila Berraf-Tebbal	MN104113	MN159112
L. mitidjana	ALG71	*Citrus*/wood canker	Algeria, Boufarik	Akila Berraf-Tebbal	MN104114	MN159113
L. mitidjana	ALG70 = MUM 19.90	*Citrus*/wood canker	Algeria, Boufarik	Akila Berraf-Tebbal	MN104115	MN159114
L. mitidjana	ALG69	*Citrus*/wood canker	Algeria, Boufarik	Akila Berraf-Tebbal	MN104116	MN159115
Diplodia seriata	ALG93	*Citrus*/wood canker	Algeria, Staoueli	Akila Berraf-Tebbal	MN104117	MN159116
D. seriata	ALG94	*Citrus*/wood canker	Algeria, Staoueli	Akila Berraf-Tebbal	MN104118	MN159117
D. seriata	ALG98	*Citrus*/wood canker	Algeria, Staoueli	Akila Berraf-Tebbal	MN104119	MN159118
D. seriata	ALG91	*Citrus*/wood canker	Algeria, Chiffa	Akila Berraf-Tebbal	MN104120	MN159119
D. seriata	ALG92	*Citrus*/wood canker	Algeria, Staoueli	Akila Berraf-Tebbal	MN104121	MN159120

D. seriata	ALG90	*Citrus*/wood canker	Algeria, Chiffa	Akila Berraf-Tebbal	MN104122	MN159121
D. seriata	ALG89	*Citrus*/wood canker	Algeria, Chiffa	Akila Berraf-Tebbal	MN104123	MN159122
D. seriata	ALG96	*Citrus*/wood canker	Algeria, Staoueli	Akila Berraf-Tebbal	MN104124	MN159123
D. seriata	ALG95	*Citrus*/wood canker	Algeria, Staoueli	Akila Berraf-Tebbal	MN104125	MN159124
D. seriata	ALG97	*Citrus*/wood canker	Algeria, Staoueli	Akila Berraf-Tebbal	MN104126	MN159125
D. mutila	ALG99	*Citrus*/wood canker	Algeria, Chiffa	Akila Berraf-Tebbal	MN104127	MN159126
D. mutila	ALG103	*Citrus*/wood canker	Algeria, Chiffa	Akila Berraf-Tebbal	MN104128	MN159127
D. mutila	ALG100	*Citrus*/wood canker	Algeria, Chiffa	Akila Berraf-Tebbal	MN104129	MN159128
D. mutila	ALG102	*Citrus*/wood canker	Algeria, Chiffa	Akila Berraf-Tebbal	MN104130	MN159129
D. mutila	ALG101	*Citrus*/wood canker	Algeria, Chiffa	Akila Berraf-Tebbal	MN104131	MN159130
Dothiorella viticola	ALG83	*Citrus*/wood canker	Algeria, Staoueli	Akila Berraf-Tebbal	MN104087	MN159086
Doth. viticola	ALG35	*Citrus*/wood canker	Algeria, Oued El Alleug	Akila Berraf-Tebbal	MN104088	MN159187
Doth. viticola	ALG84	*Citrus*/wood canker	Algeria, Chiffa	Akila Berraf-Tebbal	MN104089	MN159188
Doth. viticola	ALG85	*Citrus*/wood canker	Algeria, Staoueli	Akila Berraf-Tebbal	MN104090	MN159189
Doth. viticola	ALG86	*Citrus*/wood canker	Algeria, Oued El Alleug	Akila Berraf-Tebbal	MN104091	MN159190
Doth. viticola	ALG87	*Citrus*/wood canker	Algeria, Chiffa	Akila Berraf-Tebbal	MN104092	MN159191

Doth. viticola	ALG88	*Citrus*/wood canker	Algeria, Chiffa	Akila Berraf-Tebbal	MN104093	MN159192

Phylogenetic analysis

Sequences of all *Lasiodiplodia* species known from culture were retrieved from GenBank (S1 Table) and aligned with sequences of the isolates obtained in this study. Alignments were done with ClustalX v. 1.83 [36] using the following parameters: pairwise alignment parameters (gap opening = 10, gap extension = 0.1) and multiple alignment parameters (gap opening = 10, gap extension = 0.2, transition weight = 0.5, delay divergent sequences = 25%). Alignments were checked and manual adjustments made if necessary using BioEdit v. 7.2.5 [34]. Maximum Likelihood (ML) and Maximum Parsimony (MP) analyses were performed using MEGAX [37]. The best fitting DNA evolution model was determined also by MEGAX. ML analysis was performed on a Neighbour-Joining starting tree automatically generated by the software. Nearest-Neighbour-Interchange (NNI) was used as the heuristic method for tree inference. MP analysis was done using the Tree-Bisection-Regrafting (TBR) algorithm with search level 1 in which the initial trees were obtained by the random addition of sequences (10 replicates). The robustness of the trees (ML and MP) was evaluated by 1000 bootstrap replications.

Pathogenicity test

The ability of isolates to cause cankers was assessed *in vivo* on detached shoots collected from symptomless citrus trees. From each phylogenetically resolved species, two representative isolates were selected. Pathogenicity of each selected strain was tested on 1-year-old shoots of *Citrus sinensis.* The shoots with 25 mm in diameter were cut into equal length (25 cm long). They were then surface disinfected with 70% ethanol and wounded on an intermediate internode, with a scalpel. From each strain, a 5 mm diameter mycelial plug taken from a 5- day old colony growing on PDA was placed into the wound. Negative controls were inoculated with fresh, non-colonized, PDA plugs.
Subsequently, the cuttings were wrapped with wet sterile cotton and sealed with Parafilm® to prevent the desiccation of the agar plug. The shoots were immediately transplanted into pots containing sterilized water as a growth substrate. They were incubated at the ambient room temperature, under daily photoperiod. The water of the container was changed twice a week. There were 10 replicates per isolate, and the same number of cuttings was used as controls. One month after inoculation, lengths of lesions produced by each strain were measured. In an attempt to recover the inoculated fungi and complete Koch's postulates, necrotic tissue from the margin of the lesions was taken and placed onto PDA.

Statistical analyses

Data of lesion lengths caused by the fungal isolates belonging to the different species was subjected to one-way ANOVA (analysis of variance) with $P \leq 0.05$. Prior to analysis data were checked for normality, then, significance of differences between means was determined by

Turkey's honestly significant difference (HSD) test. Statistical analyses were performed on the software R v. 3. 5. 1 and a significance level of 0.05 was used.

Nomenclature

The electronic version of this article in Portable Document Format (PDF) in a work with an ISSN or ISBN will represent a published work according to the International Code of Nomenclature for algae, fungi, and plants, and hence the new names contained in the electronic publication of a PLOS article are effectively published under that Code from the electronic edition alone, so there is no longer any need to provide printed copies.

In addition, new names contained in this work have been submitted to MycoBank from where they will be made available to the Global Names Index. The unique MycoBank number can be resolved and the associated information viewed through any standard web browser by appending the MycoBank number contained in this publication to the prefix http://www.mycobank.org/MB/. The online version of this work is archived and available from the following digital repositories: [INSERT NAMES OF DIGITAL REPOSITORIES WHERE ACCEPTED MANUSCRIPT WILL BE SUBMITTED (PubMed Central, LOCKSS etc)].

Results

Disease symptoms

Citrus dieback was detected in all the orchards and regions investigated, with different degrees of intensity. Various external symptoms, including partial or complete dieback of the tree, branch and shoot cankers, abnormal growth of epicormic shoots; defoliation and leaf chlorosis were observed. Moreover, in certain orchards, bark cracking of the trunk and the branches was also noticeable (Fig 1).

The analysis of the 80 symptomatic sweet orange (*Citrus sinensis*) trees sampled to carry out the isolations, revealed the existence of 92 necrotic lesions in the trunks and the branches. They belonged to four types of wood alteration, including: wedge-shaped necrosis (WSN), that was the most prevalent lesion (n=30) of the total samples collected. The brown central necrosis (BCN) (n=26) was the second most prevalent lesion, followed by the black spots in the xylem (BS) (n=24) and yellow soft wood rot (YSW) (n=12).

Fig 1. Citrus tree with dieback symptoms (a), bark cracking of the trunk and gummosis (b), main internal symptoms of sectioned branches and trunks (c–f).

Fungal isolation and identification

Isolation carried out from ninety-two samples yielded a total of forty-seven fungal colonies belonging to *Botryosphaeriaceae*. On the basis of morphological characteristics, it was possible to distinguish three morphological groups according to colour and shape of conidia. Twenty-five isolates with brown sub-globose and striate conidia were grouped as *Lasiodiplodia*-like fungi. Fifteen isolates with brown oblong to ovoid conidia as *Diplodia*-like fungi. A further seven isolates with brown, ovoid thick walled and 1-septate conidia were considered as *Dothiorella*-like fungi. The identification of the isolates was confirmed by analysis of ITS and *tef1*-α sequences, which distinguished five separate species. The BLAST searches in GenBank showed 99–100% identity with reference sequences of representative isolates including that of the ex-type. The identified species were: *Diplodia seriata* (10 isolates), *Diplodia mutila* (5 isolates), *Dothiorella viticola* (7 isolates), *Lasiodiplodia mediterranea* (10 isolates) and a *Lasiodiplodia* sp. (14 isolates) that could not be assigned to any of the currently known species.

Phylogenetic analysis

Phylogenetic analysis was performed using ITS and *tef1*-α sequences. Fragments of approximately 500 and 300 bases were determined for ITS and *tef1*-α regions, respectively. The ML and the MP trees are presented in figure 2 and figure S1, respectively. The combined ITS and *tef1*-α dataset of *Lasiodiplodia* consisted of 23 isolates aligned with sequences of 69 isolates retrieved from GenBank, representing a selection of all known *Lasiodiplodia* and 2 outgroup taxa (*Diplodia seriata* CBS 112555 and *Diplodia mutila* CBS 112553). In the ML phylogenetic tree (figure 2), the isolates obtained in this study grouped in two clades. The first clade comprised 10 isolates, which clustered together, with the ex-type strain of *Lasiodiplodia mediterranea* (CBS 137783) and the ex-type strain of *Lasiodiplodia vitis* (CBS 124060) (Table S1), forming a single monophyletic group. The second group contained 14 isolates, which formed a distinct clade, with a high bootstrap support (ML/MP = 80/94), was considered to represent a distinct species, which is described here as *Lasiodiplodia mitidjana* sp. nov. (Fig 2).

Fig 2. Maximum likelihood tree generated from the combined analysis of ITS and *tef1*-α sequence data. ML/MP bootstrap values are given at the nodes. Support values less than 50 % are omitted or indicated with '–'. The tree was rooted to *Diplodia mutila* and *Diplodia seriata*.

Taxonomy

Lasiodiplodia mitidjana A. Alves, A.E. Mahamedi & A. Berraf-Tebbal **sp. nov.** (Fig 3) [urn:lsid:mycobank.org:names: MB 832823]. Algeria, Mitidja, isolated from a branch canker of *Citrus sinensis*, June 2015, Akila Berraf-Tebbal, HOLOTYPE AVE-F-7, a dried culture sporulating on pine needles twigs deposited in the Herbarium Universitatis Aveirensis

(AVE), culture ex-holotype MUM 19.90 (=ALG70). Other isolates examined are listed in Table 2.

Etymology: named after Mitidja where the fungus was discovered.

Sexual state: Not seen. Asexual state: Conidiomata stromatic, pycnidial, produced on pine needles on ¼ strength PDA within 2–3 wks, dark brown to black, covered with dense mycelium, superficial or immersed in the host becoming erumpent when mature, mostly uniloculate, solitary, globose, thick-walled. Paraphyses hyaline, cylindrical, thin-walled, initially aseptate, becoming septate when mature, rounded at apex. Conidiogenous cells holoblastic, discrete, hyaline, smooth, thin-walled, cylindrical, sometimes slightly swollen at the base. Conidia subovoid to ellipsoid-ovoid, apex rounded, occasionally tapering to truncate base, widest in middle to upper third, thick-walled, with granular content, initially hyaline and aseptate, remaining so for a long time, becoming dark brown and 1-septate, with longitudinal striations, (22.6–)27.7(−31.9) × (13.5–)16.7(−19.6) µm, 95 % confidence limits = 27.3–28 × 16.5–16.9 µm (av. of 125 conidia ± SD = 27.7 ± 1.9 × 16.7 ± 1.1 µm, L/W ratio = 1.7).

Cultural characteristics: Colonies on PDA with moderate to dense aerial mycelium, initially white to smoke-grey, turning greenish grey on the surface and reverse, becoming dark slate blue with age.

Cardinal temperatures for growth: Minimum <10 °C, maximum < 40 °C and optimum 25 – 35 °C, covering the medium surface (90 mm) before 7 days at 25 °C in the dark.

Habitat: Twigs and branches of *Citrus sinensis*.

Known geographic distribution: Algeria.

Notes: Phylogenetically it is very closely related to *L. citricola* being distinguished by three bp in the *tef1*-α locus. Conidia tend to be larger than those of *L. citricola*, 95 % confidence limits = 24.1–24.9 × 15–15.7 µm (av. ± S.D. = 24.5 ± 0.2 × 15.4 ± 1.8 µm) and have a lower L/W ratio = 1.6.

Lasiodiplodia mediterranea Linaldeddu, Deidda & Berraf-Tebbal **sp. nov.** (Linaldeddu et al. 2015. Fungal diversity 71:207)

MycoBank: MB 808356

Synonym: ***Lasiodiplodia vitis*** Yang & Crous, **sp. nov.** (Yang et al. 2017. Fungal Biology 121)

MycoBank: MB817635

Notes: Yang et al. [38] described *L. vitis* as a novel species, clearly distinct from the species recognised on *Vitis vinifera* in Italy. However, in their study Yang et al. [38] did not include any representative of *L. mediterranea* which was described by Linaldeddu et al. [28] from

several hosts, including *V. vinifera* in Italy. However, we have shown that *L. vitis* is phylogenetically indistinguishable from *L. mediterranea*. Their ITS sequences are 100% identical and the *L. vitis tef1*-α sequence deposited in GenBank differs from the *tef1*-α of *L. mediterranea* in 2 nt positions (1 missing G and a C instead of a T in the EF-986R primer binding region). We re-sequenced the *tef1*-α region of *L. vitis* CBS124060 (Table 2) using primers EF1-688F and EF1-1251R [33] which span a larger region than primers EF1-728F and EF-986R used by Yang et al. [38] and verified that these 2 nt are not real. There is no missing G in the *L. vitis* sequence and the C instead of a T is an artefact in *L. mediterranea* sequence introduced by the EF-986R primer sequence. Thus, the *tef1*-α region of *L. vitis* CBS124060 is 100% identical to the *tef1*-α sequence of *L. mediterranea*.

Fig 3. ***Lasiodiplodia mitidjana*. (a,b). Pycnidia formed on pine needles. (c). Conidiogenous layer with conidia developing on conidiogenous cells. (d). Conidia developing on conidiogenous cells and paraphyses. (e,f,i). Hyaline aseptate conidia. (g,h). Hyaline aseptate brown 1-septate conidia in two focal planes showing the striations on the inner surface of the wall. (j). Aseptate conidia, one becoming brown. (k,l). Brown 1-septate conidia in two focal planes to show the striations in the inner surface of the wall. Scale bars: e = 20 µm, c,d,f–j = 10 µm; k–l = 5 µm.**

Pathogenicity test

All the *Botryosphaeriaceae* isolates tested in the pathogenicity test were pathogenic to the citrus shoots. On the wood tissue under the bark, black to brown lesions developed, upward and downward from the inoculation point, within 30 days. The control plants did not develop any symptoms. Lesion lengths varied between the species and among the isolates of each species tested, with a significant difference ($F=10.874$; $P < 0.001$) (Table 3).

The most aggressive isolates were ALG91 (*D. seriata*) and ALG36 (*L. mediterranea*), which produced the longest lesions (5.49±2.65 cm and 4.39±1.31 cm, respectively) with a statistically significant difference recorded between ALG91 and the rest of the species, except for *L. mediterranea*. No significant difference in lesions size was observed between the isolates ALG40 (*L. mediterranea*) and ALG39 (*L. mitidjana*), which presented intermediate lesion lengths (3.83±0.97 and 3.88±1.24 cm, respectively). However, the smallest lesion size was produced by *Doth. viticola* ALG84 with 2.1±0.67 cm and both *D. mutila* isolates ALG102 (2.04±0.54) and ALG103 (2.05±0.4 cm). *D. seriata* was the only species that showed significant difference in lesion length between its two isolates (Table 3).

Koch's postulates were confirmed by a successful re-isolation of all tested fungal species from the necrotic tissues (Table 3).

Table 3. Mean lesion lengths (cm) caused by *Doth. viticola, D. mutila, D. seriata, L. mediterranea* and *L. mitidjana* species implicated in citrus dieback in northern Algeria,

30 days after inoculation of detached green branches with mycelium-colonized agar plugs.

**Species**	**Isolate**	**Mean lesion length (cm) ± SD**
D. seriata	ALG91	5.49±2.65 a
D. seriata	ALG98	2.35±0.55 cd
L. mediterranea	ALG36	4.39±1.31 ab
L. mediterranea	ALG40	3.83±0.97 abc
Doth. viticola	ALG86	2.82±0.86 bcd
Doth. viticola	ALG84	2.1±0.67 d
D. mutila	ALG102	2.04±0.54 d
D. mutila	ALG103	2.03±0.29 d
L. mitidjana	ALG39	3.88±1.24 abc
L. mitidjana	ALG34	2.2±0.67 cd

The same letter after numbers refers to the isolates that do not differ significantly according to Turkey's HSD test at $P \leq 0.05$.

Distribution of *Botryosphaeriaceae* species

Overall, the *Botryosphaeriaceae* species occurred in 42 of the 80 citrus trees showing canker and dieback symptoms. Five distinct *Botryosphaeriaceae* species were obtained in this study. Each species was found with its respective frequency, as follow: *L. citricola* (14.1%), *L. mediterranea* (13%) and *D. seriata* (10.9%), *Doth. viticola* (7.6%) and *D. mutila* (5.4%).

At least, two different species were found in each orchard. *L. mediterranea* and *Doth. viticola* were found in six of the ten surveyed orchards. They were followed by *L. mitidjana*, recorded from five orchards of two municipalities. *D. seriata* was found in four sampling sites; whereas, *D. mutila* was recovered from only two orchards of the same municipality.

Discussion

This study aimed to evaluate and characterize the diversity of the *Botryosphaeriaceae* species associated with dieback of *Citrus sinensis*. It represents the first survey and preliminary investigation of these species in the main citrus orchards in northern Algeria.

Citrus canker and dieback were detected in all regions surveyed. Several external symptoms, including partial or complete dieback of the tree, branch and shoot cankers were observed. Over time, the disease can increase and seriously affected trees can become barren and eventually, die. Similar situation has been described in several citrus orchards, worldwide [7, 15, 20, 21, 39]. According to some authors, abiotic factors, including drought, severe sunburn or freezing predispose the trees to xylem dysfunction, leading to these diseases [2, 3, 39].

In this study, five species belonging to three different genera of the *Botryosphaeriaceae* were recovered from symptomatic citrus trees, namely: *L. mediterranea*, *D. seriata*, *D. mutila*, *Doth. viticola* and *L. mitidjana*. The latter is introduced here, as new species. To our knowledge, except for *D. seriata* and *L. mediterranea*, this is the first report of *D. mutila*, *Doth. viticola* and *L. mitidjana*, causing branch canker disease on citrus and any crop, in Algeria. The *Botryosphaeriaceae* species were recovered from more than half of the trees sampled and were found in all the prospected orchards.

Lasiodiplodia was the most commonly isolated genus that was found in six of the ten surveyed orchards. This fact is consistent with previous studies, which showed that *Lasiodiplodia* species have the ability to target a wide variety of plants, distributed worldwide [40-42]. In fact, *Lasiodiplodia* species do not only occur as latent endophytes in asymptomatic plants but are also associated with different symptoms occurring on a variety of hosts including stem-end rot, fruit rot, decline, canker and dieback [41, 43-45]. In this study, *L. mediterranea* and *L. mitidjana* sp. nov. were the most frequently encountered species. *L. mediterranea* has been reported as the causal agent of canker and dieback of grapevine, holm oak as well as citrus, indicating its capability to target different hosts [28]. The latter findings lead Andolfi et al. [46] to isolate and characterize the main secondary metabolites produced by *L. mediterranea*, as well as to evaluate its phytotoxic and antifungal activities. According to the former authors [28], *L. mediterranea* has been found only on V-shaped necrotic sectors of grapevine while, it has been isolated from all the lesion types of citrus trees in this study. *L. mitidjana* sp. nov. was found in the five surveyed orchards. Isolates of this species were present predominantly in the wedge-shaped necrosis.

Dothiorella viticola was isolated at low frequency compared to *Lasiodiplodia* species found in this study. Interestingly, it was detected in six sampling sites. This species was first described as *Spencermartinisia viticola* by Phillips et al. [47]. It was obtained for the first time from *Vitis vinifera* in Spain. Recently, Yang et al. [38] regarded *Spencermartinsia* a synonym of *Dothiorella* and thus transferred the epithet *viticola* to *Dothiorella* as *Doth. viticola*. This taxonomic change was supported by a multi-gene phylogeny that included *Spencermartinsia* in *Dothiorella* genus [38, 48]. *Dothiorella viticola* has been reported from a wide range of woody hosts, including citrus trees [16, 21]. Recently, it has been also described as the causal agent of gummosis on citrus trees, in Tunisia [20]. According to Phillips et al. [18] and Dissanayake et al. [49], this species is known from China, Chile, USA, Spain, France, Australia, South Africa and Tunisia. Therefore, this study constitutes the first record of *Doth. viticola* in Algeria, which thus expands its known geographical range.

Two species of *Diplodia* genus, *D. seriata* and *D. mutila* were isolated from the surveyed orchards. *Diplodia* species are well known to cause damage on several economically important species and causing numerous disease symptoms including blight, dieback, rot diseases and canker [22, 28, 50-54]. In this study, *D. seriata* was frequently recovered from the sampling sites, which matches the findings of previous studies indicating the cosmopolitan nature of this species. This latter species is commonly reported as a pathogen on a large number of hosts and has been reported from hundreds of plant species [18, 49, 50]. *Diplodia mutila*, the second *Diplodia* species isolated in this study, was less frequently found in the prospected orchards. Moreover, to our knowledge, this is the first report of this species

in our country. In addition to Algeria, the USA is the only other country in which both *D. seriata* and *D. mutila* have been associated with citrus dieback [21]. These species have been found on apples in the USA [55, 56], Chile [57], France [58], Germany [59], Uruguay [60] and South Africa [61]; as well as in pear trees [60, 61], plum [62], peach and apricot [60, 63] and walnut [51].

All the *Botryosphaeriaceae* species of this study caused necrosis on the citrus shoots, with differences in the lengths of the lesions. These differences were observed between the species and also among isolates of the same species. Thus, the results suggest that *Diplodia seriata* and *L. mediterranea* could be considered as being the most aggressive, since they produced the longest lesion. However, *D. seriata* was significantly different compared to the rest of the isolates, which is consistent with previous studies that showed significant impact of *D. seriata* on several hosts, across the globe [28, 64-66].

For *L. mediterranea*, our results are in accordance with a previous study, which highlighted its aggressiveness in artificial inoculation experiments [28]. The least aggressive species of this case were *D. mutila* and *Doth. viticola*, producing the smallest lesion size. Nevertheless, these findings contradict previous study in which *D. mutila* was found to be the most aggressive based on lesion length [60]. According to Linaldeddu et al. [28] and Chakusary et al. [50], these differences in aggressiveness maybe due to several factors including genetic variability of isolates, age, type of host tissue, differences in susceptibility as well as inoculation methods and experimental conditions. In this case, extensive sampling from citrus as well as other hosts are required to further emphasise the findings and draw a final solid conclusion.

Overall, almost all the *Botryosphaeriaceae* species we identified have previously been detected on citrus trees with the exception of *L. mitidjana*, which was described for the first time associated with citrus dieback. Given the major impact of the *Botryosphaeriaceae* species isolated on declining trees, worldwide, it is important to emphasize the urgent need to implement prevention techniques and management strategies in order to minimize the incidence of these pathogens and to prevent their spread to new orchards. For a better understanding of citrus dieback, it is necessary to set up larger surveys that include all citrus production areas. These surveys would assess, more accurately, the impact of the trunk diseases pathogens and eventually identify the factors that influence the dieback. This will be set in order to identify a number of practices to prevent their development.

Acknowledgements

This work was supported by EFRR "Multidisciplinary research to increase application potential of nanomaterials in agricultural practice" (No. CZ.02.1.01/0.0/0.0/16_025/0007314). A. Alves acknowledges the financial support from FCT/MCTES to CESAM (UID/AMB/50017/2019), through national funds.

Author Contributions

Conceptualization: Akila Berraf-Tebbal, Artur Alves.

Data curation: Akila Berraf-Tebbal, Alla Eddine Mahamedi.

Formal analysis: Akila Berraf-Tebbal, Artur Alves.

Investigation: Akila Berraf-Tebbal, Wassila Aigoun-Mouhous, Milan Špetík, Aleš Eichmeier.

Methodology: Akila Berraf-Tebbal, Artur Alves, Alla Eddine Mahamedi.

Project administration: Akila Berraf-Tebbal, Jana Čechová, Robert Pokluda, Miroslav Baránek, Aleš Eichmeier.

Supervision: Akila Berraf-Tebbal, Artur Alves.

Writing – original draft: Akila Berraf-Tebbal**,** Alla Eddine Mahamedi, Wassila Aigoun-Mouhous.

Writing – review & editing: Akila Berraf-Tebbal, Artur Alves.

References

1. MADR. National agricultural statistics 2015. Algiers: Ministry of Agriculture and Rural Development; 2015.

2. Khanchouch K, Pane A, Chriki A, Cacciola SO. Major and emerging fungal diseases of *Citrus* in the Mediterranean region. Citrus Pathology. 2017:1. https://doi.org/10.5772/66943.

3. Bové JM. Virus and virus-like diseases of citrus in the Near East region. Rome: FAO; 1995.

4. Cohen M. Diagnosis of young tree decline, blight and sand hill decline of citrus by measurement of water uptake using gravity injection. Plant Disease Reporter. 1974;58(9):801-5.

5. Brlansky R, Lee R, Collins M. Structural comparison of xylem occlusions in the trunks of *Citrus* trees with blight and other decline diseases. Phytopathology. 1985;75(2):145-50. https://doi.org/10.1094/Phyto-75-145.

6. Elena K, Fischer M, Dimou D, Dimou DM. *Fomitiporia mediterranea* infecting citrus trees in Greece. Phytopathologia Mediterranea. 2006;45(1):35-9. https://doi.org/10.14601/Phytopathol_Mediterr-1813.

7. Polizzi G, Aiello D, Vitale A, Giuffrida F, Groenewald J, Crous P. First report of shoot blight, canker, and gummosis caused by *Neoscytalidium dimidiatum* on citrus in Italy. Plant Disease. 2009;93(11):1215-. https://doi.org/10.1094/PDIS-93-11-1215A. PMID: 30754593

8. Huang F, Hou X, Dewdney MM, Fu Y, Chen G, Hyde KD, Li H. *Diaporthe* species occurring on citrus in China. Fungal Diversity. 2013;61(1):237-50. https://doi.org/10.1007/s13225-013-0245-6.

9. Timmer L, Solel Z, Orozco-Santos M. *Alternaria* brown spot of mandarins. Compendium of citrus diseases. 2000;2:19-2000.

10. Udayanga D, Castlebury L, Rossman A, Hyde K. Species limits in *Diaporthe*: molecular re-assessment of *D. citri, D. cytosporella, D. foeniculina* and *D. rudis*. Persoonia: Molecular Phylogeny and Evolution of Fungi. 2014;32:83-101. https://doi.org/10.3767/003158514X679984. PMID: 25264384

11. Guarnaccia V, Groenewald JZ, Polizzi G, Crous PW. High species diversity in *Colletotrichum* associated with citrus diseases in Europe. Persoonia. 2017;39:32-50. https://doi.org/10.3767/persoonia.2017.39.02. PMID: 29503469

12. Polizzi G, editor Magnano di San Lio G., Catara A., 1992. Dry root rot of citranges in Italy. Proceedings of the International Society of Citriculture VII International Citrus Congress, Acireale 1992; 1992.

13. Hannachi I, Rezgui S, Cherif M. First report of mature citrus trees being affected by *Fusarium* wilt in Tunisia. Plant Disease. 2014;98(4):566. https://doi.org/10.1094/PDIS-12-12-1134-PDN. PMID: 30708695

14. Sandoval-Denis M, Guarnaccia V, Polizzi G, Crous P. Symptomatic Citrus trees reveal a new pathogenic lineage in *Fusarium* and two new *Neocosmospora* species. Persoonia: Molecular Phylogeny and Evolution of Fungi. 2018;40:1-25. https://doi.org/10.3767/persoonia.2018.40.01. PMID: 30504994

15. Mayorquin JS, Wang DH, Twizeyimana M, Eskalen A. Identification, distribution, and pathogenicity of *Diatrypaceae* and *Botryosphaeriaceae* associated with Citrus branch canker in the southern California desert. Plant Disease. 2016;100(12):2402-13. https://doi.org/10.1094/PDIS-03-16-0362-RE. PMID: 30686172

16. Adesemoye AO, Eskalen A. First Report of *Spencermartinsia viticola*, *Neofusicoccum australe*, and *N. parvum* Causing Branch Canker of Citrus in California. Plant Dis. 2011;95(6):770. https://doi.org/10.1094/PDIS-02-11-0092. PMID: 30731919

17. Trouillas FP, Pitt WM, Sosnowski MR, Huang R, Peduto F, Loschiavo A, Savocchia S, Scott ES, Gubler WD. Taxonomy and DNA phylogeny of *Diatrypaceae* associated with *Vitis vinifera* and other woody plants in Australia. Fungal Diversity. 2011;49(1):203-23. https://doi.org/10.1007/s13225-011-0094-0.

18. Phillips AJL, Alves A, Abdollahzadeh J, Slippers B, Wingfield MJ, Groenewald JZ, Crous PW. The *Botryosphaeriaceae*: genera and species known from culture. Stud Mycol. 2013;76(1):51-167. https://doi.org/10.3114/sim0021. PMID: 24302790

19. Burgess TI, Tan YP, Garnas J, Edwards J, Scarlett KA, Shuttleworth LA, Daniel R, Dann EK, Parkinson LE, Dinh Q. Current status of the Botryosphaeriaceae in Australia. Australasian Plant Pathology. 2018;48(1):35-44. https://doi.org/10.1007/s13313-018-0577-5.

20. Hamrouni N, Nouri M, Trouillas F, Said A, Sadfi-Zouaoui N, Hajlaoui M. *Dothiorella* gummosis caused by *Dothiorella viticola*, first record from citrus in Tunisia. New Disease Reports. 2018;38:10-. https://doi.org/10.5197/j.2044-0588.2018.038.010.

21. Adesemoye AO, Mayorquin JS, Wang DH, Twizeyimana M, Lynch SC, Eskalen A. Identification of Species of *Botryosphaeriaceae* Causing Bot Gummosis in Citrus in California. Plant Dis. 2014;98(1):55-61. https://doi.org/10.1094/PDIS-05-13-0492-RE. PMID: 30708572

22. Abdollahzadeh J, Javadi A, Mohammadi Goltapeh E, Zare R, Phillips AJ. Phylogeny and morphology of four new species of *Lasiodiplodia* from Iran. Persoonia. 2010;25:1-10. https://doi.org/10.3767/003158510X524150. PMID : 21339962

23. Berraf A, Péros J-P. Importance of Eutypa dieback and esca in Algeria and structure of the associated fungal community. OENO One. 2005;39(3):121-8. https://doi.org/10.20870/oeno-one.2005.39.3.896.

24. Ammad F, Benchabane M, Toumi M, Belkacem N, Guesmi A, Ameur C, Lecomte P, Merah O. Occurrence of *Botryosphaeriaceae* species associated with grapevine dieback in Algeria. Turkish Journal of Agriculture and Forestry. 2014;38(6):865-76. https://doi.org/10.3906/tar-1404-15.

25. Berraf-Tebbal A, Guereiro MA, Phillips AJ. Phylogeny of *Neofusicoccum* species associated with grapevine trunk diseases in Algeria, with description of *Neofusicoccum algeriense* sp. nov. Phytopathologia Mediterranea. 2014;53(3):416-27. https://doi.org/14385/14273.

26. Smahi H, Belhoucine-Guezouli L, Berraf-Tebbal A, Chouih S, Arkam M, Franceschini A, Linaldeddu B, Phillips A. Molecular characterization and pathogenicity of *Diplodia corticola* and other *Botryosphaeriaceae* species associated with canker and dieback of *Quercus* suber in Algeria. Mycosphere. 2017;8(2):1261-72. https://doi.org/10.5943/mycosphere/8/2/10.

27. Azouaoui-Idjer G, Della Rocca G, Pecchioli A, Bouznad Z, Danti R. First report of *Botryosphaeria iberica* associated with dieback and tree mortality of Monterey cypress (*Cupressus macrocarpa*) in Algeria. Plant Disease. 2012;96(7):1073-. https://doi.org/10.1094/PDIS-10-11-0901-PDN. PMID: 30727248

28. Linaldeddu BT, Deidda A, Scanu B, Franceschini A, Serra S, Berraf-Tebbal A, Boutiti MZ, Jamâa MB, Phillips A. Diversity of *Botryosphaeriaceae* species associated with grapevine and other woody hosts in Italy, Algeria and Tunisia, with descriptions of *Lasiodiplodia exigua* and *Lasiodiplodia mediterranea* sp. nov. Fungal Diversity. 2014;71(1):201-14. https://doi.org/10.1007/s13225-014-0301-x.

29. Santos J, Phillips A. Resolving the complex of *Diaporthe* (*Phomopsis*) species occurring on *Foeniculum vulgare* in Portugal. Fungal Diversity. 2009;34(11):111-25.

30. Alves A, Correia A, Luque J, Phillips A. *Botryosphaeria corticola*, sp. nov. on *Quercus* species, with notes and description of *Botryosphaeria stevensii* and its anamorph, *Diplodia mutila*. Mycologia. 2004;96(3):598-613. PMID: 21148880

31. White TJ, Bruns T, Lee S, Taylor J. Amplification and direct sequencing of fungal ribosomal RNA genes for phylogenetics. PCR protocols: a guide to methods and applications. 1990;18(1):315-22.

32. O'Donnell K, Cigelnik E, Nirenberg HI. Molecular systematics and phylogeography of the *Gibberella fujikuroi* species complex. Mycologia. 1998;90(3):465-93. https://doi.org/10.1080/00275514.1998.12026933.

33. Alves A, Crous PW, Correia A, Phillips A. Morphological and molecular data reveal cryptic speciation in *Lasiodiplodia theobromae*. Fungal Diversity. 2008;28:1-13. https://doi.org/10.2307/3762177.

34. Hall TA, editor BioEdit: a user-friendly biological sequence alignment editor and analysis program for Windows 95/98/NT. Nucleic acids symposium series; 1999: [London]: Information Retrieval Ltd., c1979-c2000.

35. Altschul SF, Gish W, Miller W, Myers EW, Lipman DJ. Basic local alignment search tool. J Mol Biol. 1990;215(3):403-10. https://doi.org/10.1016/S0022-2836(05)80360-2.

36. Thompson JD, Gibson TJ, Plewniak F, Jeanmougin F, Higgins DG. The CLUSTAL_X windows interface: flexible strategies for multiple sequence alignment aided by quality analysis tools. Nucleic acids research. 1997;25(24):4876-82. https://doi.org/10.1093/nar/25.24.4876. PMID: 9396791

37. Kumar S, Stecher G, Li M, Knyaz C, Tamura K. MEGA X: Molecular Evolutionary Genetics Analysis across Computing Platforms. Mol Biol Evol. 2018;35(6):1547-9. https://doi.org/10.1093/molbev/msy096. PMID: 29722887

38. Yang T, Groenewald JZ, Cheewangkoon R, Jami F, Abdollahzadeh J, Lombard L, Crous PW. Families, genera, and species of Botryosphaeriales. Fungal biology. 2017;121(4):322-46. https://doi.org/10.1016/j.funbio.2016.11.001. PMID: 28317538

39. Raimondo F, Nardini A, Salleo S, Cacciola SO, Gullo MAL. A tracheomycosis as a tool for studying the impact of stem xylem dysfunction on leaf water status and gas exchange in *Citrus aurantium* L. Trees. 2010;24(2):327-33. https://doi.org/10.1007/s00468-009-0402-4.

40. Slippers B, Johnson GI, Crous PW, Coutinho TA, Wingfield BD, Wingfield MJ. Phylogenetic and morphological re-evaluation of the *Botryosphaeria* species causing diseases of *Mangifera indica*. Mycologia. 2005;97(1):99-110. https://doi.org/10.3852/mycologia.97.1.99. PMID: 16389961

41. Sakalidis ML, Ray JD, Lanoiselet V, Hardy GES, Burgess TI. Pathogenic *Botryosphaeriaceae* associated with Mangifera indica in the Kimberley region of Western Australia. European journal of plant pathology. 2011;130(3):379-91. https://doi.org/10.1007/s10658-011-9760-z.

42. Rodríguez-Gálvez E, Guerrero P, Barradas C, Crous PW, Alves A. Phylogeny and pathogenicity of *Lasiodiplodia* species associated with dieback of mango in Peru. Fungal biology. 2017;121(4):452-65. https://doi.org/10.1016/j.funbio.2016.06.004. PMID: 28317545

43. Ismail A, Cirvilleri G, Polizzi G, Crous P, Groenewald J, Lombard L. *Lasiodiplodia* species associated with dieback disease of mango (*Mangifera indica*) in Egypt. Australasian Plant Pathology. 2012;41(6):649-60. https://doi.org/10.1007/s13313-012-0163-1.

44. Marques MW, Lima NB, de Morais MA, Barbosa MAG, Souza BO, Michereff SJ, Phillips AJ, Câmara MP. Species of *Lasiodiplodia* associated with mango in Brazil. Fungal Diversity. 2013;61(1):181-93. https://doi.org/10.1007/s13225-013-0231-z.

45. Trakunyingcharoen T, Lombard L, Groenewald JZ, Cheewangkoon R, To-Anun C, Crous P. Caulicolous Botryosphaeriales from Thailand. Persoonia: Molecular Phylogeny and Evolution of Fungi. 2015;34:87–99. https://doi.org/10.3767/003158515X685841. PMID: 26240447

46. Andolfi A, Basso S, Giambra S, Conigliaro G, Lo Piccolo S, Alves A, Burruano S. Lasiolactols A and B produced by the grapevine fungal pathogen *Lasiodiplodia mediterranea*. Chemistry & biodiversity. 2016;13(4):395-402. https://doi.org/10.1002/cbdv.201500104. PMID: 26938016

47. Phillips A, Alves A, Pennycook S, Johnston P, Ramaley A, Akulov A, Crous P. Resolving the phylogenetic and taxonomic status of dark-spored teleomorph genera in the *Botryosphaeriaceae*. Persoonia: Molecular Phylogeny and Evolution of Fungi. 2008;21:29-55. https://doi.org/10.3767/003158508X340742. PMID: 20396576

48. Slippers B, Boissin E, Phillips A, Groenewald JZ, Lombard L, Wingfield MJ, Postma A, Burgess T, Crous PW. Phylogenetic lineages in the Botryosphaeriales: a systematic and evolutionary framework. Studies in Mycology. 2013;76(1):31-49. https://doi.org/10.3114/sim0020. PMID: 24302789

49. Dissanayake A, Phillips A, Li X, Hyde K. *Botryosphaeriaceae*: current status of genera and species. Mycosphere. 2016;7(7):1001-73. https://doi.org/10.5943/mycosphere/si/1b/13.

50. Chakusary MK, Mohammadi H, Khodaparast SA. Diversity and pathogenicity of *Botryosphaeriaceae* species on forest trees in the north of Iran. European Journal of Forest Research. 2019:1-20. https://doi.org/10.1007/s10342-019-01200-7.

51. Chen S, Morgan DP, Hasey JK, Anderson K, Michailides TJ. Phylogeny, morphology, distribution, and pathogenicity of *Botryosphaeriaceae* and *Diaporthaceae* from English Walnut in California. Plant Dis. 2014;98(5):636-52. https://doi.org/10.1094/PDIS-07-13-0706-RE. PMID: 30708543

52. Lazzizera C, Frisullo S, Alves A, Lopes J, Phillips A. Phylogeny and morphology of *Diplodia* species on olives in southern Italy and description of *Diplodia olivarum* sp. nov. Fungal Divers. 2008;31:63-71.

53. Phillips AJ, Crous PW, Alves A. *Diplodia seriata*, the anamorph of "*Botryosphaeria*" *obtusa*. Fungal Diversity. 2007;25:141-55.

54. Úrbez-Torres J, Peduto F, Vossen P, Krueger W, Gubler W. Olive twig and branch dieback: etiology, incidence, and distribution in California. Plant Disease. 2013;97(2):231-44. https://doi.org/10.1094/PDIS-04-12-0390-RE. PMID: 30722318

55. Crespo M, Moral J, Michailides T, Trouillas F. First report of black rot on apple fruit caused by *Diplodia seriata* in California. Plant Disease. 2018;102(4):824. https://doi.org/10.1094/PDIS-07-17-1023-PDN.

56. Kim Y, Kwak J, Aguilar C, Xiao C. First report of black rot on apple fruit caused by *Diplodia seriata* in Washington State. Plant Disease. 2016;100(7):1499-. https://doi.org/10.1094/PDIS-12-15-1463-PDN.

57. Díaz GA, Latorre BA, Ferrada E, Lolas M. Identification and characterization of *Diplodia mutila*, *D. seriata*, *Phacidiopycnis washingtonensis* and *Phacidium lacerum* obtained from apple (*Malusx domestica*) fruit rot in Maule region, Chile. European journal of plant pathology. 2019;153(1):211-25. https://doi.org/10.1007/s10658-018-01640-8.

58. Giraud M. Apple Black Rot. Infos-Ctifl. 2009(257):36-41.

59. Weber R, Dralle N. Fungi associated with blossom-end rot of apples in Germany. Eur J Hort Sci. 2013;78:97-105.

60. Sessa L, Abreo E, Bettucci L, Lupo S. *Botryosphaeriaceae* species associated with wood diseases of stone and pome fruits trees: symptoms and virulence across different hosts in Uruguay. European journal of plant pathology. 2016;146(3):519-30. https://doi.org/10.1007/s10658-016-0936-4.

61. Cloete M, Fourie PH, Damm U, Crous PW, Mostert L. Fungi associated with dieback symptoms of apple and pear trees, a possible inoculum source of grapevine trunk disease pathogens. Phytopathologia Mediterranea. 2011;50:S176-S90. https://doi.org/10.14601/Phytopathol_Mediterr-9004.

62. Damm U, Crous PW, Fourie PH. *Botryosphaeriaceae* as potential pathogens of Prunus species in South Africa, with descriptions of *Diplodia africana* and *Lasiodiplodia plurivora* sp. nov. Mycologia. 2007;99(5):664-80. https://doi.org/10.3852/mycologia.99.5.664. PMID: 18268901

63. Laundon G. *Botryosphaeria obtusa*, *B. stevensii*, and *Otthia spiraeae* in New Zealand. Transactions of the British Mycological Society. 1973;61(2):369-IN17. https://doi.org/10.1016/S0007-1536(73)80158-5.

64. Larignon P. Maladies cryptogamiques du bois de la vigne: symptomatologie et agents pathogènes. Institut Français de la Vigne et du Vin, Grau du Roi dans le Gard. 2012

65. Luque J, Martos S, Aroca A, Raposo R, Garcia-Figueres F. Symptoms and fungi associated with declining mature grapevine plants in northeast Spain. Journal of Plant Pathology. 2009:381-90.

66. Morales A, Latorre BA, Piontelli E, Besoain X. *Botryosphaeriaceae* species affecting table grape vineyards in Chile and cultivar susceptibility. Ciencia e investigación agraria. 2012;39(3):445-58. https://doi.org/10.4067/S0718-16202012000300005.

Supplementary table S1. Details of strains included in the phylogenetic and/or morphological analyses.

**Species**	**Culture collection number(s)**[1]	**Substrate**	**Country**	**Collector(s)**	**GenBank accession numbers**	
					**ITS**	***tef1***
L. avicenniae	CMW 41467	*Avicennia*, asymptomatic branches	South Africa	J. A. Osorio & J. Roux	KP860835	KP860680
L. avicenniae	LAS199	*Avicennia*, asymptomatic branches	South Africa	J. A. Osorio & J. Roux	KU587957	KU587947
L. brasiliense	CMM 4015, ex-type	*Mangifera*, stems	Brazil	M. W. Marques	JX464063	JX464049
L. brasiliense	CMM 4469	*Anacardium*	Brazil	-	KT325574	KT325580
L. bruguierae	CMW 41470	*Bruguiera*¸ asymptomatic branches	South Africa	J. A. Osorio & J. Roux	KP860833	KP860678
L. bruguierae	CMW 42480	*Bruguiera*, asymptomatic branches	South Africa	J. A. Osorio & J. Roux	KP860832	KP860677
L. caatinguensis	CMM 1325	*Citrus*	Brazil	I. B. L. Coutinho & J. S. Lima	KT154760	KT008006
L. caatinguensis	IBL 381	*Spondias*	Brazil	J. S. Lima & J. E. Cardoso	KT154757	KT154751
L. chinensis	CGMCC 3.18061	*Unknown*, branch	China	W. He & Z. P. Dou	KX499889	KX499927
L. chinensis	CGMCC 3.18044	*Vaccinium*, branch	China	J. H. Zhao	KX499875	KX499913
L. cinnamomi	CFCC 51997	*Cinnamomum*, branch	China	N. Jiang	MG866028	MH236799
L. cinnamomi	CFCC 51998	*Cinnamomum*, branch	China	N. Jiang	MG866029	MH236800

L. citricola	CBS 124706	*Citrus* sp., twigs	Iran	A. Shekari	GU945353	GU945339
L. citricola	CBS 124707, ex-type	*Citrus*, twigs	Iran	J. Abdollahzadeh & A. Javadi	GU945354	GU945340
L. crassispora	CMW 13488	*Eucalyptus*, wood	Venezuela	S. Mohali	DQ103552	DQ103559
L. crassispora	CBS 118741, ex-type	*Santalum*	Australia	T. I. Burgess & B. Dell	DQ103550	EU673303
L. euphorbicola	CMW 33350	*Adansonia*	Botswana	-	KU887149	KU887026
L. euphorbicola	CMW 36231	*Adansonia*	Zimbabwe	-	KU887187	KU887063
L. exigua	BL 184	*Retama*, branch canker	Tunisia	B. T. Linaldeddu	KJ638318	KJ638337
L. exigua	CBS 137785, ex-type	*Retama*, branch canker	Tunisia	B. T. Linaldeddu	KJ638317	KJ638336
L. gilanensis	CBS 124704, ex-type	*Citrus*, fallen twigs	Iran	J. Abdollahzadeh & A. Javadi	GU945351	GU945342
L. gilanensis	CBS 124705	*Citrus* sp., fallen twigs	Iran	J. Abdollahzadeh & A. Javadi	GU945352	GU945341
L. gonubiensis	CMW 14077, ex-type	*Syzygium*	South Africa	D. Pavlic	AY639595	DQ103566
L. gonubiensis	CMW 14078, ex-paratype	*Syzigium*	South Africa	D. Pavlic	AY639594	DQ103567
L. gravistriata	CMM 4564	*Anacardium*, stems	Brazil	M. S. B. Netto	KT250949	KT250950
L. gravistriata	CMM 4565	*Anacardium*, stems	Brazil	M. S. B. Netto	KT250947	KT266812
L. hormozganensis	CBS 124708	*Mangifera*, twigs	Iran	J. Abdollahzadeh & A. Javadi	GU945356	GU945344

L. hormozganensis	CBS 124709, ex-type	*Olea*, twigs	Iran	J. Abdollahzadeh & A. Javadi	GU945355	GU945343
L. hyalina	CGMCC 3.17975	*Acacia*, cankered stems	China	Y. Zhang & Y. P. Zhou	KX499879	KX499917
L. hyalina	CGMCC 3.18383	unidentified woody plant, cankered branches	China	Z. P. Dou & Z. C. Liu	KY767661	KY751302
L. iraniensis	IRAN 1520C, ex-type	*Salvadora*, twigs	Iran	J. Abdollahzadeh & A. Javadi	GU945346	GU945334
L. iraniensis	IRAN 1502C	*Juglans*, twigs	Iran	A. Javadi	GU945347	GU945335
L. laeliocattleyae	CBS 167.28	*Laeliocattleya*, leaves	Italy	C. Sibilia	KU507487	KU507454
L. laeliocattleyae	CBS 130992	*Mangifera*, leaves	Egypt	A. M. Ismail	JN814397	JN814424
L. lignicola	CBS 134112	Wood	Thailand	-	JX646797	KU887003
L. macrospora	CMM 3833, ex-type	*Jatropha*, collar and root rot	Brazil	A. R. Machado & O. L. Pereira	KF234557	KF226718
L. mahajangana	CMW 27801, ex-type	*Terminalia*, healthy branches	Madagascar	J. Roux	FJ900595	FJ900641
L. mahajangana	CMW 27820	*Terminalia*, healthy branches	Madagascar	J. Roux	FJ900597	FJ900643
L. margaritacea	CBS 122519, ex-type	*Adansonia*, dying twigs	Australia	T. I. Burgess & M. J. Wingfield	EU144050	EU144065
L. margaritacea	CBS 122065	*Adansonia gibbosa*, dying twigs	Australia	T. I. Burgess	EU144051	EU144066
L. mediterranea	CBS 137783, ex-type	*Quercus*, branch canker	Italy	B. T. Linaldeddu	KJ638312	KJ638331
L. mediterranea	CBS 137784	*Vitis*, brown stripe under the bark	Italy	S. Serra	KJ170150	KJ170151

L. missouriana	CBS 128311, ex-type	Wedge-shape canker of grapevine cv. Catawba (complex hybrid of North America *Vitis* species)	USA	K. Striegler & G. M. Leavitt	HQ288225	HQ288267
L. missouriana	CBS 128312	Wedge-shape canker of grapevine cv. Catawba (complex hybrid of North America *Vitis* species)	USA	K. Striegler & G. M. Leavitt	HQ288226	HQ288268
L. parva	CBS 456.78, ex-type	Cassava-field soil	Colombia	O. Rangel	EF622083	EF622063
L. parva	CBS 494.78	Cassava-field soil	Colombia	O. Rangel	EF622084	EF622064
L. plurivora	STE-U 5803, ex-type	*Prunus*, wood canker	South Africa	U. Damm	EF445362	EF445395
L. plurivora	STE-U 4583	*Vitis*, symptomatic	South Africa	F. Halleen	AY343482	EF445396
L. pontae	CMM 1277	*Spondias*, necrotic canker	Brazil	J.S. Lima & F.C.O. Freire	KT151794	KT151791
L. pseudotheobromae	CBS 116459, ex-type	*Gmelina*, twigs	Costa Rica	J. Carranza- Velazquez	EF622077	EF622057
L. pseudotheobromae	CGMCC 3.18047	*Pteridium*, twigs	China	-	KX499876	KX499914
L. pyriformis	CBS 121770, ex-type	*Pinus*, fruiting structures	Namibia	F. J. J. van der Walt & J. Roux	EU101307	EU101352
L. pyriformis	CBS 121771	*Pinus*, fruiting structures	Namibia	F. J. J. van der Walt & J. Roux	EU101308	EU101353
L. rubropurpurea	WAC 12535, ex-type	*Eucalyptus*, canker	Australia	T. I. Burgess	DQ103553	EU673304

L. rubropurpurea	WAC 12536	*Eucalyptus*, canker	Australia	T. I. Burgess	DQ103554	DQ103572
L. sterculiae	CBS 342.78, ex-type	*Sterculia*	Germany	S. Bruhn	KX464140	KX464634
L. subglobosa	CMM 3872, ex-type	*Jatropha*, collar and root rot	Brazil	A. R. Machado & O. L. Pereira	KF234558	KF226721
L. subglobosa	CMM 4046	*Jatropha*	Brazil	A. R. Machado & O. L. Pereira	KF234560	KF226723
L. thailandica	CGMCC 3.18382	*Podocarpus*, cankered branch	China	D. Zhipeng & L. Zuchen	KY767662	KY751303
L. thailandica	CGMCC 3.18384	*Albizia*, cankered branch	China	Z.P. Dou & Z.C. Liu	KY767663	KY751304
L. theobromae	CBS 164.96, ex-neotype	Fruit along coral reef coast	Papua New Guinea	A. Aptroot	AY640255	AY640258
L. theobromae	CBS 111530	*Leucospermum*	USA	J. E. Taylor	EF622074	EF622054
L. venezuelensis	WAC 12539, ex-type	*Acacia*, wood	Venezuela	S. Mohali	DQ103547	EU673305
L. venezuelensis	WAC 12540	*Acacia*, wood	Venezuela	S. Mohali	DQ103548	DQ103569
L. viticola	CBS 128313, ex-type	Wedge-shape canker of grapevine cv. Vignoles (complex hybrid of North America *Vitis* species)	USA	R. D. Cartwright & W. D. Gubler	HQ288227	HQ288269
L. viticola	UCD 2604MO	*Vitis*	USA	J. R. Urbez-Torres	HQ288228	HQ288270
L. vitis	CBS 124060, ex-type	*Vitis*, wood fragment	Italy, Sicily	S. Burruano	KX464148	KX464642
D seriata	CBS 112555	*Vitis*, dead stems	Portugal	A. J. L. Phillips	AY259094	AY573220

D mutila	CBS 112553	*Vitis*	Portugal	A. Alves	AY259093	AY573219

[1] BL: Personal number of B.T. Linaldeddu; CBS: CBS-KNAW Fungal Biodiversity Centre, Utrecht, The Netherlands; CFCC: China Forestry Culture Collection Center, Beijing, China; CMM: Culture Collection of Phytopathogenic Fungi “Prof. Maria Menezes”, Universidade Federal Rural de Pernambuco, Recife, Brazil; CMW: Tree Patholgy Co-operative Program, Forestry and Agricultural Biotechnology Institute, University of Pretoria, South Africa; IRAN: Iranian Fungal Culture Collection, Iranian Research Institute of Plant Protection, Iran; WAC: Department of Agriculture, Western Australia Plant Pathogen Collection, South Perth, Western Australia.

Supplementary figure S1.

Maximum Parsimony phylogenetic tree resulting from the analysis of the combined ITS and *tef1-α* sequence data from *Lasiodiplodia* species. The tree was rooted to *Diplodia mutila* and *Diplodia seriata*.

Dear Dr. Katharina B Budde,

We would like to thank you for considering our manuscript PONE-D-19-29810, entitled "*Lasiodiplodia mitidjana* sp. nov. and other *Botryosphaeriaceae* species causing branch canker and dieback of *Citrus sinensis* in Algeria", for publication in PLOS ONE Journal.

We would like to thank you for the helpful comments and constructive observations. We have made all the suggested revisions. A detailed point-by-point response is reported here below and all new changes have been highlighted in yellow in the revised version of our manuscript.

All authors read and approved the submitted new version of the manuscript.

Best regards,

Dr. AKILA BERRAF-TEBBAL

Reviewer Comments to Author:

Answers to the reviewer 1

General comments:
Akila Berraf-Tebbal and collaborators present in this manuscript the results of a survey on Botryosphaeriaceae diversity and pathogenicity affecting symptomatic sweet orange (Citrus sinensis) in Algeria. Botryosphaeriacea species were identified in every orchard tested (n=10) and a total of five different species have been isolated from symptomatic samples, with frequencies ranging from 5.4% to 14.1% of the samples. One species appears to form a distinct monophyletic group - not described before - and is claimed by the authors to be a new species. The pathogenicity of two representative isolates for every species isolated (n=5) was tested experimentally on Citrus shoot and Koch's postulate was verified for all of them. Differences in pathogenicity was observed between isolates.
Overall, the manuscript is well written and there is no doubt about the efforts made by the authors to produce this work. The results presented are interesting and alert about the potential spread and threats brought by these pathogens, as mentioned by the authors.
My main comments to improve this manuscript is that authors often goes on conclusions that are not completely supported by the results. I suggest therefore to reconsider these conclusions or change the way they are presented to stick more on the facts.

We took into consideration all the comments regarding the conclusions and we made sure to do all the required modifications.

Furthermore, the GenBank accession for the Tef1-α sequences are not available which hamper my reviewing conclusions. This is even more problematic because the genetic difference observed to discriminate the new species are brought by this marker.

The ITS and the *tef1*-α sequences have been deposited into GenBank; However, the *tef1*-α sequences are not automatically deposited into GenBank after being accessioned. Each sequence record is individually examined and processed by the GenBank annotation staff to ensure that it is free of errors or problems.

In this sense, a new species (Lasiodiplodia mitidja) is introduced in this study. This introduction is based on a two loci phylogeny, as well as morphological observations. I'm not a taxonomist myself, but are two SNPs (which I could not verified, and that is not illustrated by an alignment neither in the manuscript) and a bootstrap of 80 enough to consider the organism a new species? Concerning the morphological differences, as the authors mentioned, conidia "tend" to be larger and L/W ratio is different but for both measurements, no statistical significance is brought to the observations to confirm the difference. Can it represent a subpopulation of L. citricola? I presume it is not possible to test if those "species" outcross but if we have to be more rigorous, I would recommend to stay more prudent about the "new species" terminology and presented it more as a suggestion, or inform the readers that all the criteria to say it's a new species are not completely fulfil.

We agree that this may be debatable. In fact, we have discussed this previously within the team. However, it is clearly aligned with the current trend for introduction of novel *Lasiodiplodia* species.

In the future it may well be proven that in fact it is not a new species different from *L. citricola*. But taxonomy is dynamic and hence frequently changing. For the moment we would like to introduce the new species. The fact is that eventually it will be described as a new species, if not by our group, then by someone else.

As an example of a case which is similar to ours, here are the nucleotide differences for the following mentioned species: ***L. chinensis* vs *L. lignicola* vs *L. pseudotheobromae*.**
For all 3 species ITS is 100 % identical

As for Tef1:

L. chinensis* vs *L. lignicola: 1 nucleotide difference

L. chinensis* vs *L. pseudotheobromae: 3 nucleotides differences

My second concern is the ambiguity made between the types of wood alteration/symptoms observed in Citrus trees and the presence and implication of Botryosphaeriaceae. Botryosphaeriaceae species can be isolated from certain types of alteration and yet not being responsible of these alterations. Knowing the "opportunist" behaviour of these

pathogens, I would not be surprised if they take over the habitat after a disequilibrium was induced into the microbiome of trees following another pathogen attack.

We agree with the reviewer. After considering your other comments on the same part and your suggestion of removing it and given that it is not relevant for the paper we decided to delete it.

The fact that the isolates were able to provoke symptoms experimentally does not necessarily mean they are responsible of the ones observed on the diseased trees, especially since the symptoms observed after artificial inoculation are not correlated to the ones observed on fields.

We agree with the reviewer, however the goal was to test pathogenicity of the isolates and this is the way to do it. Of course we cannot be sure that they will behave the same way in the field but they have the potential to do so.

Similarly, the presence of basidiocarps on heavily symptomatic Citrus is confusing for me, at least the way it is presented. What is the link between the Botryosphaeriaceae and the basidiocarps emergence, which species correspond to this basidiocarp?

We described the health status of the orchards where the sampling has been carried out (branch and shoot cankers, abnormal growth of epicormic shoots; defoliation and leaf chlorosis). Basidiocarps are the fruiting bodies of the decay-causing fungi. Their presence on the trunk means that it is an already rotting trunk and that some ascomycetes (Botryosphaeriaceae, Diatrypaceae…) and Basidiomycetes (Fomitiporia, Phellinus….) have already colonized the trunk.

In a similar fashion, Figure 4 is confusing as my conclusion on this figure is that Botryosphaeriaceae can be isolated from different types of symptoms and not that one species is more isolated from one type of symptoms than another as the authors tend to say. There is no statistic proving so, and a quick interpretation (but false) from a hurried reader would be that such species is responsible of such symptoms. At this point, those results are more detrimental that beneficial to the study. I either recommend to delete this part or erase those ambiguities by a deeper discussion and a clearer result presentation. What do we know about the multifactorial aspects of dieback diseases? Is there only one pathogen involved? I think study conducted on Botryosphaeriaceae and grapevine trunk disease can be related to this case. Furthermore, if this part is conserved, more insights on what is known about the different symptoms that can cause Botryosphaeriaceae could/should be presented in the introduction.

We agree with the reviewer and his comments. After pointing out these remarks we thoughtfully considered them and decided to delete the figure 4 as well as the paragraphs related to it.

Finally, the statistical methods used to test pathogenicity differences is either not well presented or the conclusions are not correct. This part needs to be improve. Have you tested species effect? Isolate effect? What are the p-value attributed to each ANOVA test, which factor has been tested by the ANOVA? Is LSD method (which is not described by the way) the more appropriate in your case?

We took into consideration your valuable comments and we made sure to change this part and we removed all the ambiguities.

Minor Comments:

L30: 14.1% percent of the samples and 13% of the samples

R: Revised as recommended

L31: what is the difference between widespread and abundant?

R: Widespread means that it is found or distributed over a large area. However, abundant means that it is existing or available in large quantities (it could have the same meaning as plentiful)

L42: I would erase (pomelos)

R: Revised as recommended

L43: Despite the high adaptation capacity of citrus trees to different climates (reference is missing)

R: Revised as recommended

L47: Citrus diseases are numerous and diverse, and are caused by phytopathogenic agents belonging to viruses, viroids, phytoplasmas, bacteria, and fungi (reference is missing).

R: Revised as recommended

L57: reference is missing

R: Revised as recommended

L63: colonize or affect?

R: We deleted 'affect'

This part on Botryosphaeriaceae should be more documented: classification of Botryosphaeriaceae, how many genera, endophytes with symptomless period, etc…

R: Revised as recommended

L82: Surveys were conducted in ten commercial orchards in the northern region of Algeria, specifically, in the Mitidja plain at the base of the Tell Atlas Mountains (Table 1).

Table 1: I would add coordinates of the orchards and years of sampling.

R: Revised as recommended

L94: was the scalpel sterilized?

R: Yes, the scalpel was sterilized. This detail has been added to the manuscript.

L112: In this paragraph, can you add more infos about the PCR conditions?
R: Revised as recommended

L120-121: pyrosequencing?
R: the company used Sanger sequencing method.

L123: Newly generated sequences were deposited in GenBank (Table 2): the Tef1-α isn't accessible.
R: The sequences are available in GenBank.

L124: Sequences for both DNA regions were retrieved in BLAST searches from GenBank [34].
Check the meaning of this sentence. For example: "Homological sequences of the newly sequenced ones were retrieved from the GenBank by Blast."

R: Revised as recommended

L125: Table 2: Can you add more info on this table, like type of tissue (trunk/branches), type of symptoms (under your classification), Orchard, etc….

R: Revised as recommended

L132: Please specify the request you made, or put the sequence in the supplemental files.

R: Revised as recommended (the sequences are in the supplementary files)

L136: what kind of adjustments?

R: The ITS and *tef1*-α sequences were initially aligned separately using ClustalX v. 1.83. The alignments were manually optimized by coding the missing sequences as "?". Ambiguous sequences at the start and the end were deleted and gaps were adjusted in BioEdit.

L153: diameter

R: Revised as recommended

L164: this part need to be more precise/improve. Which threshold to accept the significance of the ANOVA, which factor tested, what LSD means for, which soft did you use?

R: Revised as recommended

Detailed responses:

Which threshold to accept the significance of the ANOVA

R: when the P value is below the threshold (0.05), the difference between the means is considered as significant.

Which factor tested?

R: We tested the lesions produced by each fungal isolate of the different species.

What LSD means for?

R: We changed the statistical test by using Tukey's honestly significant difference (HSD) test.

Which soft did you use?

R: The R v. 3.5.1 statistical software was used to perform the statistical analysis.

L171: nothing is said about the distribution

R: We removed the word 'distribution' from the title

L172-173 and L174-175 could be fused for clarity purposes

R: We removed this paragraph as recommended.

L175: the total number of samples… Samples = branches?

R: The samples mean the different necrotic lesions found in the branches and the trunks of the 80 trees.

L176 – 177: this measurement is completely arbitrary and according to me abusive. If one pathogen would have occurred at 80% frequency, the difference between 11%(very frequent) and 4%(infrequent) would be meaningless. My advice, stick to the numbers and do not try to interpreted it in a frequent or infrequent way, that's too subjective.

R: We removed the paragraph related to the frequency of occurrence, as recommended.

L186: On heavily infected trees, basidiocarps emerged: that's ambiguous, as said before.
R: We removed the description of the basidiocarps from the photoplate as well as from the text, as recommended.

L189: why wedge-shaped necrosis is not name WSN?

R: Revised as recommended

L191: similar BCN instead of BCN and YSW instead of NCC.

R: Revised as recommended

L196: the "e." is missing on the picture, but maybe see in it as a sign for not putting this picture…

R: Revised as recommended

L214: Why MP tree is not shown? At least in supplemental file?

R: Revised as recommended (the tree is in supplementary file)

L218: why 23 isolates and not 24 (10+14)

R: The sequence of one isolate was not good enough to use it for the phylogenetic analysis.

L228: The phylogenetic tree of only one method is presented, although the bootstrap values of the two methods are shown. Can we see the tree constructed with the second method in the supplemental files?

R: Revised as recommended

L279: can you have the sequence accessible please?

R: The sequence has been submitted to GenBank. It will be available online after verification of the annotation. We have included the accession number into the table 2.

L311: from what I've read, LSD test is not recommended anymore as sensitive to multiple comparison. Furthermore, as mentioned above, this test is not well conducted and presented. A histogram would be better, with a sign for significant difference, either at the isolate level and species level, with threshold use for significance. The 100% of re-isolation frequency for every isolates are not necessary in the table according to me, if you say it in the text.

R: Revised as recommended.

L317: Ambiguous: are you speaking of distribution in the wood? If yes, cut this paragraph in two: Frequency of occurrence / Distribution of Botryosphaeriaceae in the wood.

R: Revised as recommended

L320: I comment already the frequent and very frequent ranking, that's abusive according to me.

R: We deleted this paragraph.

L323: For each orchard, at least two different species were isolated, average per orchard?

R: In the paragraph L323, we described the widespread of the species in the orchards. It was only to mention their presence on each orchard.

For more details, here is a table containing all the information about species distribution among the surveyed orchards.

	**Region**										
	**Oued El Alleug**				**Chiffa**		**Boufarik**		**Staoueli**		
**Species/ Orchards**	**1**	**2**	**3**	**4**	**5**	**6**	**7**	**8**	**9**	**10**	**Total**
D. seriata	-	-	-	-	2	1	-	-	3	4	10
D. mutila	-	-	-	-	3	2	-	-	-	-	5
L. mediterr.	2	2	3	1	-	-	2	2	-	-	12
L. mitidjana	0	3	3	3	-	-	2	2	-	-	13

Doth. vitic.	1	-	1	-	1	2	-	-	1	1	7
**Total**	3	5	7	4	6	5	4	4	4	5	47

L328: new paragraph or no paragraph at all as mentioned above, I found this part confusing.

R: Revised as recommended (We removed the paragraph).

L370-371: According to these authors, L. mediterranea has been found only on V-shaped necrotic sectors of grapevine while it has been isolated from all the lesion types of citrus trees in this study.

R: Revised as recommended

L373: what the results of Andolfi et al. bring to your results?

R: Andolfi et al. (2016) isolated and characterized the main secondary metabolites produced by *L. mediterranea*. They also, evaluated its phytotoxic and antifungal activities. These findings support our results, which show the ability of this fungal species to colonize and cause damages in the wood.

L374-375: maybe if you had isolated only the 4 or 5 isolates that were from brown central necrosis, would you have said that the species was exclusively found in brown central necrosis? This part of the discussion is not really constructive.

R: we removed the paragraph, as well as the figure 4.

L387: More interesting that this, it confirms its wide geographical range.

R: revised as recommended

L402: Wedge shaped lesion?

R: The paragraph is about the pathogenicity trial and the lesions produced by each *Botryosphaeriaceae* species. We did not consider the shape of the lesions, for the pathogenicity test.

L403-405: this part of the discussion goes beyond what your data show, either improve your statistical analysis or moderate the message.

R: Revised as recommended

L408-415: similar, hard to have this kind of discussion with two isolates per strains, with one phenotyping trial.

R: Revised as recommended

L446: References have to be reformatted: species not in italic, capital letter on every first word letter for some references, etc.; some other problem like L508 Phililips is written bizarrely.

R: Revised as recommended

<u>Answers to the reviewer 2</u>

Reviewer #2: This is a nice study of Botryosphaeriaceae which are important plant pathogens, including fruit trees. Algeria is unexplored both from mycological and pathological perspective and it is nice to see collaborations like this resulting in a good piece of work. I agree with authors regarding Lasiodiplodia mediterranea and L.vitis situation, especially because pcr atifacts introduced by primer sequences are unfortunately a common thing these days (my personal experience). I have small suggestions that would improve the paper.

Abstract, line 25-of Botryosphaeriaeceae

R: Revised as recommended

Abstract, line 29- Delete which, add Lasiodiplodia mitidjana is described in this paper as a new species

R: Revised as recommended

Abstract, line 62- delete effect

R: Revised as recommended

Materials-line 98-dried on sterilized paper (towels, filter paper?)

R: Revised as recommended

Materials, line 100-The mycelium emerging from wood pieces was transferred…

R: Revised as recommended

Materials, line 105-Isolates that lacked pycnidia production…

R: Revised as recommended

Materials, line 150-How did you select representative isolates?

R: We selected two isolates, from each phylogenetically resolved species.

Materials, line 151-Shoots? But above you mentioned branches (line 148)

R: Revised as recommended (We have standardized using shoot instead of branch).

Materials, line 157-"… well watered and maintained under favorable conditions" What do you mean by this? Were the cuttings in soil or in water? How many times per week did you change the water? Ambient temperature? Light?

R: The inoculated cuttings were wrapped with wet sterile cotton to avoid the desiccation of the agar plug. The shoots were immediately transplanted into pots containing sterilized water as a growth substrate (10 shoots per pot), which were incubated at the ambient room temperature, under daily photoperiod. The water of the container was changed twice a week.

Results, line 182-Degrees of intensity? Where are they?

R: The degrees of intensity refer to the different levels of the dieback symptoms observed in the orchards.

Results, line 186-Basidiocarps of which species or genera?

R: We did not identify the basidiocarps. We described all the symptoms related to the citrus trees dieback, including the fruiting bodies emerged from the trunks.

Results, line 309-What about control plants?

R: We did not isolate any of the tested species from the negative control.

Results, line 313-Now you mention branches again

R: revised as recommended

Discussion, line 360-and seriously affected trees can become

R: revised as recommended

Discussion, lines 403-404- “However, D. seriata was significantly different compared to the rest of the isolates” But previously you said that both D. seriata and L. mediterranea were most aggressive species (based on lesion lengths). So which species was in fact most aggressive? Also, what about differences in aggressiveness between different isolates of the same species?

So which species was in fact most aggressive?

R: The significant difference was made based on a comparison between all the tested isolates. It was not about the pairwise comparisons that take one isolate and compare it with each of the rest of isolates. *D. seriata* and *L. mediterranea* were the most aggressive species when compared to the rest of the species. However, *D. seriata* was the most aggressive species, considering the length of the lesion for each isolate, separately.

Also, what about differences in aggressiveness between different isolates of the same species?

R: Significant variation in aggressiveness can occur within and among isolates from the same species. This aggressiveness refers to the quantitative variation of pathogenicity on the susceptible host infection efficiency, the latent period, the spore production rate and the infectious period of each strain. These components are closely related to the genetic variability within the strains of the same species.

Discussion, lines 408-412-Was this previous study also about Bot on citrus trees?

R: the study was about *Lasiodiplodia* species (*Botryosphaeriaceae*) on grapevine.

Discussion, lines 409 and 411-In line 404 you are talking about aggressiveness. Now about virulence. In line 413 you talk again about aggressiveness. Virulence and aggressiveness don’t mean the same thing. Replace the term virulence with aggressiveness in lines 409 and 411.

R: Revised as recommended

Answers to the academic editor

L.273 ..we have shown…

R: Revised as recommended

L. 279 "…these 2 nt are not real." Are not real is confusing, maybe instead use " were correctly determined"?

R: Revised as recommended

L. 335 "The remaining species…" better mention the species' name here.

R: Revised as recommended

L. 351 A similar situation has been…

R: Revised as recommended

L. 363 worLd

R: Revised as recommended

L. 373 compared instead of comparing

R: Revised as recommended

L. 389 "…known for targeting economically important plants…" targeting sounds as if they are selecting the hosts based on the economical value. Maybe better say: …known to cause damage on several economically important species..."

R: Revised as recommended

l. 393 "The later…", better say "This latter species.." or just "It..."

R: Revised as recommended

L. 408 …with a previous study…

R: Revised as recommended

I would suggest to add some research perspectives at the end of the discussion. What kind of studies could help to better understand and develop management recommendations against citrus dieback?

R: Revised as recommended
