# Supplementary material for: Lasiodiplodia mitidjana sp. nov. and other Botryosphaeriaceae species causing branch canker and dieback of Citrus sinensis in Algeria
Source: PLoS One. 2020 May 20;15(5):e0232448. doi: 10.1371/journal.pone.0232448 (PMC7239386; doi:10.1371/journal.pone.0232448)
Supplement: Attachment — Submitted filename: Response to Reviewers.docx [file pone.0232448.s004.docx]

**Dear Dr. Katharina B Budde,**

**We would like to thank you for considering our manuscript PONE-D-19-29810, entitled
"Lasiodiplodia mitidjana sp. nov. and other *Botryosphaeriaceae* species causing branch canker and dieback of Citrus sinensis in Algeria", for publication in PLOS ONE Journal.**

**We would like to thank you for the helpful comments and constructive observations. We have made all the suggested revisions. A detailed point-by-point response is reported here below and all new changes have been highlighted in yellow in the revised version of our manuscript.**

**All authors read and approved the submitted new version of the manuscript.**

**Best regards,**

**Dr. AKILA BERRAF-TEBBAL**

**Reviewer Comments to Author:**

**Answers to the reviewer 1**

**General comments**:
Akila Berraf-Tebbal and collaborators present in this manuscript the results of a survey on Botryosphaeriaceae diversity and pathogenicity affecting symptomatic sweet orange (Citrus sinensis) in Algeria. Botryosphaeriacea species were identified in every orchard tested (n=10) and a total of five different species have been isolated from symptomatic samples, with frequencies ranging from 5.4% to 14.1% of the samples. One species appears to form a distinct monophyletic group - not described before - and is claimed by the authors to be a new species. The pathogenicity of two representative isolates for every species isolated (n=5) was tested experimentally on Citrus shoot and Koch’s postulate was verified for all of them. Differences in pathogenicity was observed between isolates.
Overall, the manuscript is well written and there is no doubt about the efforts made by the authors to produce this work. The results presented are interesting and alert about the potential spread and threats brought by these pathogens, as mentioned by the authors.
My main comments to improve this manuscript is that authors often goes on conclusions that are not completely supported by the results. I suggest therefore to reconsider these conclusions or change the way they are presented to stick more on the facts.

We took into consideration all the comments regarding the conclusions and we made sure to do all the required modifications.

Furthermore, the GenBank accession for the Tef1-α sequences are not available which hamper my reviewing conclusions. This is even more problematic because the genetic difference observed to discriminate the new species are brought by this marker.

The ITS and the *tef1*-α sequences have been deposited into GenBank; However, the *tef1*-α sequences are not automatically deposited into GenBank after being accessioned. Each sequence record is individually examined and processed by the GenBank annotation staff to ensure that it is free of errors or problems.

In this sense, a new species (Lasiodiplodia mitidja) is introduced in this study. This introduction is based on a two loci phylogeny, as well as morphological observations. I’m not a taxonomist myself, but are two SNPs (which I could not verified, and that is not illustrated by an alignment neither in the manuscript) and a bootstrap of 80 enough to consider the organism a new species? Concerning the morphological differences, as the authors mentioned, conidia “tend” to be larger and L/W ratio is different but for both measurements, no statistical significance is brought to the observations to confirm the difference. Can it represent a subpopulation of L. citricola? I presume it is not possible to test if those “species” outcross but if we have to be more rigorous, I would recommend to stay more prudent about the “new species” terminology and presented it more as a suggestion, or inform the readers that all the criteria to say it’s a new species are not completely fulfil.

We agree that this may be debatable. In fact, we have discussed this previously within the team. However, it is clearly aligned with the current trend for introduction of novel *Lasiodiplodia* species.

In the future it may well be proven that in fact it is not a new species different from *L. citricola*. But taxonomy is dynamic and hence frequently changing. For the moment we would like to introduce the new species. The fact is that eventually it will be described as a new species, if not by our group, then by someone else.

As an example of a case which is similar to ours, here are the nucleotide differences for the following mentioned species: ***L. chinensis* vs *L. lignicola* vs *L. pseudotheobromae*.** For all 3 species ITS is 100 % identical

As for Tef1:

***L. chinensis* vs *L. lignicola*:** 1 nucleotide difference

***L. chinensis* vs *L. pseudotheobromae*:** 3 nucleotides differences

My second concern is the ambiguity made between the types of wood alteration/symptoms observed in Citrus trees and the presence and implication of Botryosphaeriaceae. Botryosphaeriaceae species can be isolated from certain types of alteration and yet not being responsible of these alterations. Knowing the “opportunist” behaviour of these pathogens, I would not be surprised if they take over the habitat after a disequilibrium was induced into the microbiome of trees following another pathogen attack.

We agree with the reviewer. After considering your other comments on the same part and your suggestion of removing it and given that it is not relevant for the paper we decided to delete it.

The fact that the isolates were able to provoke symptoms experimentally does not necessarily mean they are responsible of the ones observed on the diseased trees, especially since the symptoms observed after artificial inoculation are not correlated to the ones observed on fields.

We agree with the reviewer, however the goal was to test pathogenicity of the isolates and this is the way to do it. Of course we cannot be sure that they will behave the same way in the field but they have the potential to do so.

Similarly, the presence of basidiocarps on heavily symptomatic Citrus is confusing for me, at least the way it is presented. What is the link between the Botryosphaeriaceae and the basidiocarps emergence, which species correspond to this basidiocarp?

We described the health status of the orchards where the sampling has been carried out (branch and shoot cankers, abnormal growth of epicormic shoots; defoliation and leaf chlorosis). Basidiocarps are the fruiting bodies of the decay-causing fungi. Their presence on the trunk means that it is an already rotting trunk and that some ascomycetes (Botryosphaeriaceae, Diatrypaceae…) and Basidiomycetes (Fomitiporia, Phellinus….) have already colonized the trunk.

In a similar fashion, Figure 4 is confusing as my conclusion on this figure is that Botryosphaeriaceae can be isolated from different types of symptoms and not that one species is more isolated from one type of symptoms than another as the authors tend to say. There is no statistic proving so, and a quick interpretation (but false) from a hurried reader would be that such species is responsible of such symptoms. At this point, those results are more detrimental that beneficial to the study. I either recommend to delete this part or erase those ambiguities by a deeper discussion and a clearer result presentation. What do we know about the multifactorial aspects of dieback diseases? Is there only one pathogen involved? I think study conducted on Botryosphaeriaceae and grapevine trunk disease can be related to this case. Furthermore, if this part is conserved, more insights on what is known about the different symptoms that can cause Botryosphaeriaceae could/should be presented in the introduction.

We agree with the reviewer and his comments. After pointing out these remarks we thoughtfully considered them and decided to delete the figure 4 as well as the paragraphs related to it.

Finally, the statistical methods used to test pathogenicity differences is either not well presented or the conclusions are not correct. This part needs to be improve. Have you tested species effect? Isolate effect? What are the p-value attributed to each ANOVA test, which factor has been tested by the ANOVA? Is LSD method (which is not described by the way) the more appropriate in your case?

We took into consideration your valuable comments and we made sure to change this part and we removed all the ambiguities.

**Minor Comments:**

**L30: 14.1% percent of the samples and 13% of the samples**

R: Revised as recommended

**L31: what is the difference between widespread and abundant?**

R: Widespread means that it is found or distributed over a large area. However, abundant means that it is existing or available in large quantities (it could have the same meaning as plentiful)

**L42: I would erase (pomelos)**

R: Revised as recommended

**L43: Despite the high adaptation capacity of citrus trees to different climates (reference is missing)**

R: Revised as recommended

**L47: Citrus diseases are numerous and diverse, and are caused by phytopathogenic agents belonging to viruses, viroids, phytoplasmas, bacteria, and fungi (reference is missing).**

R: Revised as recommended

**L57: reference is missing**

R: Revised as recommended

**L63: colonize or affect?**

R: We deleted ‘affect’

**This part on Botryosphaeriaceae should be more documented: classification of Botryosphaeriaceae, how many genera, endophytes with symptomless period, etc…**

R: Revised as recommended

**L82: Surveys were conducted in ten commercial orchards in the northern region of Algeria, specifically, in the Mitidja plain at the base of the Tell Atlas Mountains (Table 1).**

**Table 1: I would add coordinates of the orchards and years of sampling.**

R: Revised as recommended

**L94: was the scalpel sterilized?**

R: Yes, the scalpel was sterilized. This detail has been added to the manuscript.

**L112: In this paragraph, can you add more infos about the PCR conditions?**
R: Revised as recommended

**L120-121: pyrosequencing?**
R: the company used Sanger sequencing method.

**L123: Newly generated sequences were deposited in GenBank (Table 2): the Tef1-α isn’t accessible.**
R: The sequences are available in GenBank.

**L124: Sequences for both DNA regions were retrieved in BLAST searches from GenBank [34].
Check the meaning of this sentence. For example: “Homological sequences of the newly sequenced ones were retrieved from the GenBank by Blast.”**

R: Revised as recommended

**L125: Table 2: Can you add more info on this table, like type of tissue (trunk/branches), type of symptoms (under your classification), Orchard, etc….**

R: Revised as recommended

**L132: Please specify the request you made, or put the sequence in the supplemental files.**

R: Revised as recommended (the sequences are in the supplementary files)

**L136: what kind of adjustments?**

R: The ITS and *tef1*-α sequences were initially aligned separately using ClustalX v. 1.83. The alignments were manually optimized by coding the missing sequences as “?”. Ambiguous sequences at the start and the end were deleted and gaps were adjusted in BioEdit.

**L153: diameter**
R: Revised as recommended

**L164: this part need to be more precise/improve. Which threshold to accept the significance of the ANOVA, which factor tested, what LSD means for, which soft did you use?**

R: Revised as recommended

Detailed responses:

**Which threshold to accept the significance of the ANOVA**

R: when the P value is below the threshold (0.05), the difference between the means is considered as significant.

**Which factor tested?**

R: We tested the lesions produced by each fungal isolate of the different species.

**What LSD means for?**

R: We changed the statistical test by using Tukey's honestly significant difference (HSD) test.

**Which soft did you use?**

R: The R v. 3.5.1 statistical software was used to perform the statistical analysis.

**L171: nothing is said about the distribution**

R: We removed the word ‘distribution’ from the title

**L172-173 and L174-175 could be fused for clarity purposes**

R: We removed this paragraph as recommended.

**L175: the total number of samples… Samples = branches?**

R: The samples mean the different necrotic lesions found in the branches and the trunks of the 80 trees.

**L176 – 177: this measurement is completely arbitrary and according to me abusive. If one pathogen would have occurred at 80% frequency, the difference between 11%(very frequent) and 4%(infrequent) would be meaningless. My advice, stick to the numbers and do not try to interpreted it in a frequent or infrequent way, that’s too subjective.**

R: We removed the paragraph related to the frequency of occurrence, as recommended.

**L186: On heavily infected trees, basidiocarps emerged: that’s ambiguous, as said before.**
R: We removed the description of the basidiocarps from the photoplate as well as from the text, as recommended.

**L189: why wedge-shaped necrosis is not name WSN?**

R: Revised as recommended

**L191: similar BCN instead of BCN and YSW instead of NCC.**

R: Revised as recommended

**L196: the “e.” is missing on the picture, but maybe see in it as a sign for not putting this picture…**

R: Revised as recommended

**L214: Why MP tree is not shown? At least in supplemental file?**

R: Revised as recommended (the tree is in supplementary file)

**L218: why 23 isolates and not 24 (10+14)**

R: The sequence of one isolate was not good enough to use it for the phylogenetic analysis.

**L228: The phylogenetic tree of only one method is presented, although the bootstrap values of the two methods are shown. Can we see the tree constructed with the second method in the supplemental files?**

R: Revised as recommended

**L279: can you have the sequence accessible please?**

R: The sequence has been submitted to GenBank. It will be available online after verification of the annotation. We have included the accession number into the table 2.

**L311: from what I’ve read, LSD test is not recommended anymore as sensitive to multiple comparison. Furthermore, as mentioned above, this test is not well conducted and presented. A histogram would be better, with a sign for significant difference, either at the isolate level and species level, with threshold use for significance. The 100% of re-isolation frequency for every isolates are not necessary in the table according to me, if you say it in the text.**

R: Revised as recommended.

**L317: Ambiguous: are you speaking of distribution in the wood? If yes, cut this paragraph in two: Frequency of occurrence / Distribution of Botryosphaeriaceae in the wood.**

R: Revised as recommended

**L320: I comment already the frequent and very frequent ranking, that’s abusive according to me.**

R: We deleted this paragraph.

**L323: For each orchard, at least two different species were isolated, average per orchard?**

R: In the paragraph L323, we described the widespread of the species in the orchards. It was only to mention their presence on each orchard.

For more details, here is a table containing all the information about species distribution among the surveyed orchards.

|  | **Region** | | | | | | | | | |  |
| --- | --- | --- | --- | --- | --- | --- | --- | --- | --- | --- | --- |
|  | **Oued El Alleug** | | | | **Chiffa** | | **Boufarik** | | **Staoueli** | |  |
| **Species/ Orchards** | **1** | **2** | **3** | **4** | **5** | **6** | **7** | **8** | **9** | **10** | **Total** |
| ***D. seriata*** | - | - | - | - | 2 | 1 | - | - | 3 | 4 | 10 |
| ***D. mutila*** | - | - | - | - | 3 | 2 | - | - | - | - | 5 |
| ***L. mediterr.*** | 2 | 2 | 3 | 1 | - | - | 2 | 2 | - | - | 12 |
| ***L. mitidjana*** | 0 | 3 | 3 | 3 | - | - | 2 | 2 | - | - | 13 |
| ***Doth. vitic.*** | 1 | - | 1 | - | 1 | 2 | - | - | 1 | 1 | 7 |
| **Total** | 3 | 5 | 7 | 4 | 6 | 5 | 4 | 4 | 4 | 5 | 47 |

**L328: new paragraph or no paragraph at all as mentioned above, I found this part confusing.**

R: Revised as recommended (We removed the paragraph).

**L370-371: According to these authors, L. mediterranea has been found only on V-shaped necrotic sectors of grapevine while it has been isolated from all the lesion types of citrus trees in this study**.

R: Revised as recommended

**L373: what the results of Andolfi et al. bring to your results?**

R: Andolfi et al. (2016) isolated and characterized the main secondary metabolites produced by *L. mediterranea*. They also, evaluated its phytotoxic and antifungal activities. These findings support our results, which show the ability of this fungal species to colonize and cause damages in the wood.

**L374-375: maybe if you had isolated only the 4 or 5 isolates that were from brown central necrosis, would you have said that the species was exclusively found in brown central necrosis? This part of the discussion is not really constructive.**

R: we removed the paragraph, as well as the figure 4.

**L387: More interesting that this, it confirms its wide geographical range.**

R: revised as recommended

**L402: Wedge shaped lesion?**

R: The paragraph is about the pathogenicity trial and the lesions produced by each *Botryosphaeriaceae* species. We did not consider the shape of the lesions, for the pathogenicity test.

**L403-405: this part of the discussion goes beyond what your data show, either improve your statistical analysis or moderate the message.**

R: Revised as recommended

**L408-415: similar, hard to have this kind of discussion with two isolates per strains, with one phenotyping trial.**

R: Revised as recommended

**L446: References have to be reformatted: species not in italic, capital letter on every first word letter for some references, etc.; some other problem like L508 Phililips is written bizarrely.**

R: Revised as recommended

**Answers to the reviewer 2**

**Reviewer #2: This is a nice study of Botryosphaeriaceae which are important plant pathogens, including fruit trees. Algeria is unexplored both from mycological and pathological perspective and it is nice to see collaborations like this resulting in a good piece of work. I agree with authors regarding Lasiodiplodia mediterranea and L.vitis situation, especially because pcr atifacts introduced by primer sequences are unfortunately a common thing these days (my personal experience). I have small suggestions that would improve the paper.**

**Abstract, line 25-of Botryosphaeriaeceae**

R: Revised as recommended

**Abstract, line 29- Delete which, add Lasiodiplodia mitidjana is described in this paper as a new species**

R: Revised as recommended

**Abstract, line 62- delete effect**

R: Revised as recommended

**Materials-line 98-dried on sterilized paper (towels, filter paper?)**

R: Revised as recommended

**Materials, line 100-The mycelium emerging from wood pieces was transferred…**

R: Revised as recommended

**Materials, line 105-Isolates that lacked pycnidia production…**

R: Revised as recommended

**Materials, line 150-How did you select representative isolates?**

R: We selected two isolates, from each phylogenetically resolved species.

**Materials, line 151-Shoots? But above you mentioned branches (line 148)**

R: Revised as recommended (We have standardized using shoot instead of branch).

**Materials, line 157-“… well watered and maintained under favorable conditions” What do you mean by this? Were the cuttings in soil or in water? How many times per week did you change the water? Ambient temperature? Light?**

R: The inoculated cuttings were wrapped with wet sterile cotton to avoid the desiccation of the agar plug. The shoots were immediately transplanted into pots containing sterilized water as a growth substrate (10 shoots per pot), which were incubated at the ambient room temperature, under daily photoperiod. The water of the container was changed twice a week.

**Results, line 182-Degrees of intensity? Where are they?**

R: The degrees of intensity refer to the different levels of the dieback symptoms observed in the orchards.

**Results, line 186-Basidiocarps of which species or genera?**

R: We did not identify the basidiocarps. We described all the symptoms related to the citrus trees dieback, including the fruiting bodies emerged from the trunks.

**Results, line 309-What about control plants?**

R: We did not isolate any of the tested species from the negative control.

**Results, line 313-Now you mention branches again**

R: revised as recommended

**Discussion, line 360-and seriously affected trees can become**

R: revised as recommended

**Discussion, lines 403-404- “However, D. seriata was significantly different compared to the rest of the isolates” But previously you said that both D. seriata and L. mediterranea were most aggressive species (based on lesion lengths). So which species was in fact most aggressive? Also, what about differences in aggressiveness between different isolates of the same species?**

**So which species was in fact most aggressive?**

R: The significant difference was made based on a comparison between all the tested isolates. It was not about the pairwise comparisons that take one isolate and compare it with each of the rest of isolates. *D. seriata* and *L. mediterranea* were the most aggressive species when compared to the rest of the species. However, *D. seriata* was the most aggressive species, considering the length of the lesion for each isolate, separately.

**Also, what about differences in aggressiveness between different isolates of the same species?**

R: Significant variation in aggressiveness can occur within and among isolates from the same species. This aggressiveness refers to the quantitative variation of pathogenicity on the susceptible host infection efficiency, the latent period, the spore production rate and the infectious period of each strain. These components are closely related to the genetic variability within the strains of the same species.

**Discussion, lines 408-412-Was this previous study also about Bot on citrus trees?**

R: the study was about *Lasiodiplodia* species (*Botryosphaeriaceae*) on grapevine.

**Discussion, lines 409 and 411-In line 404 you are talking about aggressiveness. Now about virulence. In line 413 you talk again about aggressiveness. Virulence and aggressiveness don’t mean the same thing. Replace the term virulence with aggressiveness in lines 409 and 411.**

R: Revised as recommended

**Answers to the academic editor**

**L.273 ..we have shown…**

R: Revised as recommended

**L. 279 “…these 2 nt are not real.” Are not real is confusing, maybe instead use “ were correctly determined”?**

R: Revised as recommended

**L. 335 “The remaining species…” better mention the species’ name here.**

R: Revised as recommended

**L. 351 A similar situation has been…**

R: Revised as recommended

**L. 363 worLd**

R: Revised as recommended

**L. 373 compared instead of comparing**

R: Revised as recommended

**L. 389 “…known for targeting economically important plants…” targeting sounds as if they are selecting the hosts based on the economical value. Maybe better say: …known to cause damage on several economically important species...”**

R: Revised as recommended

**l. 393 “The later…”, better say “This latter species..” or just “It...”**

R: Revised as recommended

**L. 408 …with a previous study…**

R: Revised as recommended

**I would suggest to add some research perspectives at the end of the discussion. What kind of studies could help to better understand and develop management recommendations against citrus dieback?**

R: Revised as recommended
