# Supplementary material for: Lasiodiplodia mitidjana sp. nov. and other Botryosphaeriaceae species causing branch canker and dieback of Citrus sinensis in Algeria
Source: PLoS One. 2020 May 20;15(5):e0232448. doi: 10.1371/journal.pone.0232448 (PMC7239386; doi:10.1371/journal.pone.0232448)
Supplement: Attachment — Submitted filename: Response to Reviewers.docx [file pone.0232448.s006.docx]

**Dear Dr. Katharina B Budde,**

**We would like to thank you for considering our manuscript PONE-D-19-29810, entitled
"Lasiodiplodia mitidjana sp. nov. and other *Botryosphaeriaceae* species causing branch canker and dieback of Citrus sinensis in Algeria", for publication in PLOS ONE Journal.**

**Once again, we appreciate your efforts and the reviewers’ efforts in providing helpful comments to improve the manuscript. We made sure to answer all the questions and to bring the necessary changes to the manuscript, as advised by the reviewers. A detailed point-by-point response is reported here below and all new changes have been highlighted in yellow in the revised version of the manuscript.**

**Best regards,**

**Dr. AKILA BERRAF-TEBBAL**

**Answers to the academic editor**

I recommend starting out the introduction with a wider scope. Citrus cultivation is not only important in Algeria but in many countries and the fruits are being exported and eaten in even more countries, therefore I would start out with some sentences about the importance of Citrus in general. This could make the publication more attractive to a wider readership.

**R: Revised as recommended**

The authors mention at the end of the introduction that Botryosphaeriaceae have not been studied in detail in Algeria. Have other pathogen families or groups on Citrus already been studied in more detail? I think this should be introduced here in detail! This could also explain why the authors only focus on Botryosphaeriaceae and not check which other pathogens are causing the symptoms in their samples. Please explain that in more detail!

**R: In this part of the introduction, we mentioned that there has not been any thorough study about citrus in Algeria. Until today, trunk diseases caused by *Botryosphaeriaceae* species has been widely reported in Algeria and has been associated with many important crops such as grapevine, date palm and some other fruit and forest trees. This fact led us to focus only on *Botryosphaeriaceae* in this study.**

**Regarding citrus dieback, the only previous study done so far is the one published by Linaldeddu et al. (2015) where we described *Lasiodiplodia mediterranea* based on few samples. Thus, in the present study, we decided to collect more samples from several orchads in order to sudy the diversity of the *Botryosphaeriaceae* and their association with citrus diaback. Indeed, this study represents the most comprehensive study on the presence, diversity and pathogenicity of *Botryosphaeriaceae* species associated with declining citrus trees in Algeria.**

The objectives at the end of the introduction should be described in more detail! The authors do not even mention that they also aimed at testing the aggressiveness of the the isolates!

**R: Revised as recommended**

Table 1, I agree with Benoit Laurent that it would be relevant (if possible) to add the GPS coordinates of each orchard in each region as it would give an idea of how far apart the orchards are. If this information cannot be given, I would rather remove the GPS coordinates of the cities.

**R: Revised as recommended (Unfortunately, we were not able to get the exact GPS coordinates of each orchard. Thus, as advised, we removed the GPS coordinates from the table).**

l. 145 Mention which was the best fitting DNA evolution model.

**R: Revised as recommended**

Please improve the description of the statistical analyses and test specifically for differences in aggressiveness between species as suggested by Benoit Laurent.

**R: Revised as recommended**

I am a little surprised about the paragraph called “Nomenclature”. I am not familiar with the description of new fungal species but I have never seen such a justification. Is this typical? Otherwise, it should be removed. I suggest to name this paragraph “Taxonomy” which should be mentioned that it has been described in detail and that the new names have been submitted to MycoBank.

**R: Regarding the Nomenclature, we included it because it was one of the requirements of the journal, for the description of new species. I believe that adding it or removing it all depends on the choice of the editor. I checked two papers, one of them has it and the other does not.**

In the results the authors mention that they obtained 47 fungal colonies belonging to Botryosphaeriaceae. Did they also observe other fungi in their isolates? What happened to the other samples?

**R: In this study*, Botryosphaeriaceae* species accounted for more than 50% of the isolates, making this by far the most common taxonomic group associated with branch cankers in declining citrus trees. As mentioned previously in the introduction, we wanted to focus our study only on *Botryosphaeriaceae* due to their important impact worldwide. Regarding the remaining isolates, in addition to the wood samples which did not develop any fungus, we obtained the following genera: *Alternaria*, *Pestalotiopsis* and some sterile fungi, which we could not identify. These samples were not considered in the study due to their low frequency as well as to our focus on *Botryosphaeriaceae.***

l. 41 clementines

**R: Revised as recommended**

l. 62-64 This sentence is unclear. Do the authors refer to a single species of the Botryosphaeriaceae family or several that are all important pathogens? Please clarify!

**R: Towards the end of the same paragraph, we have mentioned all the *Botryosphaeriaceae* species belonging to several genera that impact the citrus, as described in different studies.**

l. 77 …citrus trees in Algeria…

**R: Revised as recommended**

l. 350 …similar situations have been described…

**R: Revised as recommended**

l. 351 …according to some authors… is not a good formulation!

**R: Revised as recommended (we replaced it by, ‘According to previous studies’)**

**Answers to the reviewer 1**

**General comments**:
Reviewer #1: I would like to thank Akila Berraf-Tebbal and his collaborators to the answers they brought to my questions and comments. The data on the tef1 alpha are now available, which was one major concern of my last review, and allow me to verify the monophyletic group corresponding to the introduction of a new species. The debate on this introduction is still questionable but does not question the quality and the presentation of the work. I don’t think that following a trend is really constructive and yet scientific, but I endorse their point of view about the dynamicity of the taxonomy.

Besides, I’m rather surprised that, in response to my comment about the lack of general information on Botryosphaeriaceae, a sentence is brought from a reference and citing “It comprises 24 genera encompassing 222 species”, which is a rather static way of introducing the phylogeny of Botryosphaeriaceae. I would rather say “It comprises at least 24 genera encompassing 222 species”, or “It comprises at least 24 known genera encompassing 222 known species”.

**R: Revised as recommended**

Overall, I find the manuscript clearer as it is and have only one major suggestion to make this manuscript in the standard for publication. It concerns the pathogenicity test, the statistical analysis conducted on it and its interpretation. First of all, I suggest to add in the material and method (Pathogenicity test section) that lesion size was used as a proxy to measure the aggressiveness of the isolate. As you mentioned to one of the other reviewer comment, aggressiveness is a complex trait and proxies are often used, and are always good to define in the text.

**R: Revised as recommended (we mentioned that internal lesion length was used as a proxy to measure the aggressiveness of the isolates)**

Then, the "Statistical analyzes" section from the Materials and Methods has still a margin of improvement. In your case, the ANOVA was used to test the genotype effect on the lesion size variation, with significance accepted for P<0.05. The test used to test the normality of the data could be also presented. Finally, what you can say so far is that some isolates belonging to some species was more aggressive than other isolates, belonging maybe to other species. But, you didn’t test the species effect, which would appreciate the variation of the means per species and test if the means are different. Hence you cannot say that one species is more aggressive than another one (L403 in the discussion, and the whole paragraph suggest so as it is presented). If the standard deviation is too important (D. seriata for example), therefore you’ll need more genotype per species to reach statistical significance and confirm those species effects, and then used HSD to identify which species is statistically different from the others. Therefore, I suggest to adapt the discussion in consequence, or add more genotypes in the pathogenicity test if possible.
Finally, I hope this section will be more accurately presented and this manuscript will be published as the results from this work will be beneficial for the community.

**R: Revised as recommended (The Shapiro-Wilk test conducted for normality checking was presented. As for the species effect, we agree with the reviewer that we need to conduct further tests on other isolates for each species in order to get clear conclusion about differences in aggressiveness between species. Unfortunately, due to some circumstances, it would be almost impossible to perform these tests. Thus, we made sure to adapt the discussion accordingly and take into consideration the reviewer’s comments.)**

Minor comments:
I’m surprised by the answer of one of my comment about the basidiocarp observed in some citrus orchard:“Basidiocarps are the fruiting bodies of the decay-causing fungi. Their presence on the trunk means that it is an already rotting trunk and that some ascomycetes (Botryosphaeriaceae, Diatrypaceae…) and Basidiomycetes (Fomitiporia, Phellinus….) have already colonized the trunk.” Is that always true? For me, some decay-causing fungi are able to provoke the rotting of the trunk by themselves (e.g. Heterobasidion on pine trees).

**R: I agree with the reviewer. What I meant, back in the first review, is that in our case, all the basidiocarps were observed in already rotting trunk. Indeed, it is true that it is not always the case. As the reviewer mentioned, some decay-causing fungi are able to provoke the rotting of the trunk by themselves.**

L95: There may have a misunderstanding in my comment. I was more interested in the GPS location of the orchards than the GPS location of the city. If the authors don’t have access to this information, this is not a big deal. The aim of my comment was to give additional information that could feed the discussion about the distribution pattern of the Bot species. Was L. mitidjana sp. nov. isolated both year? In similar orchard (tree ages?)

**R: Regarding the GPS coordinates, unfortunately, we were not able to obtain the exact coordinates of the orchards. As advised by the editor, we will remove the city coordinates since they are not relevant. Regarding the isolates, different samples from different orchards have been collected during the time period from 2013 to 2015. Thus, replying to question of the reviewer, we did not collect samples from the same orchard each year. All the prospected orchards had approximately the same age (between 25 and 30 years old).**

L374: “L. mitidjana sp. nov. was found in the five surveyed orchards. “ suggests that L. mitidjana sp. nov. was isolated from all the surveyed orchards. I suggest “L. mitidjana sp. nov. was found on five orchards out of ten”, or “L. mitidjana sp. nov. was found in five of the surveyed orchards”.

**R: Revised as recommended**

Furthermore, do you have some elements to discuss the distribution of the species? Is there any particularity on these locations? The table containing all the information about species distribution and presented in response to my comment is particularly interesting. I’m surprised this table is not presented, at least in supplemental file.

**R: Revised as recommended (the table is in the supplementary files)**

L397: “our country” is maybe to personal

**R: Revised as recommended (we replaced it by Algeria)**

**Answers to the reviewer 3**

Reviewer #3: Dear Authors
I found this piece of work really interesting, well done, with a very good language, robust methodology and, much important, the article has been strongly improved after the first revisions.

A very few small notes are included in the attached pdf.

**R: Revised as recommended**

Furthermore, my only big concern, as mentioned by another reviewer, is about the description of the new Lasiodiplodia species. But I have read what you authors already have replied to the first reviewer and I can understand your point of view.
So, I accept your reasons and your determination to consider this as a novel species.
Probably, just to be clearer in the manuscript, it could be useful to sequence the other fundamental locus for the Botryosphaeriaceae identification, tubulin.
Of course it would be crazy to re-open the phylogenetic analysis, but at least, right because you consider it a new species, having the TUB sequences deposited in Genbank would be nice for future studies. And maybe, you can also mention and discuss something about nucleotide differences in TUB between your new species and L. citricola.
But I confirm, the analysis and phylogenetic tree are good and I consider them great to achieve the aim of this study.

**R: Thank you for your comments and for understanding. We agree with your point. We could sequence the TUB and deposit it in GenBank and have them available for future studies. To be honest, in the meantime, it is hard to sequence the TUB due to the present circumstances and all what is happening around the word. We agree that this may be debatable. In fact, we have discussed this previously with the second reviewer. However, our point is that our work is clearly aligned with the current trend for introduction of novel *Lasiodiplodia* species. In fact, in all studies, TEF1 is considered as the most informative locus used to ID species for the *Botryosphaeriaceae*. We understand that the taxonomy is dynamic and hence frequently changing. Yet, for the moment we would like to introduce the new species. The fact is that eventually it will be described as a new species, if not by our group, then by someone else. This is why, if possible, we would appreciate if the acceptance for publication of this manuscript is not delayed because of that.**

**As an example of a case which is similar to ours, here are the nucleotide differences for the following mentioned species: *L. chinensis* vs *L. lignicola* vs *L. pseudotheobromae*. For all 3 species ITS is 100 % identical. We aligned the TUB sequences of these species and obtained 100% of similarity between them. As for Tef1: *L. chinensis* vs *L. lignicola*: 1 nucleotide difference *L. chinensis* vs *L. pseudotheobromae*: 3 nucleotides differences**

The authors cite the articles number 8,9,10,11. I don’t think the paper number 11 is related with Diaporthe in Europe. It should be removed. On the contrary, recent studies on DIaporthe which should be cited are:

-Guarnaccia, V., & Crous, P. W. (2018). Species of Diaporthe on Camellia and Citrus in the Azores Islands. Phytopathologia Mediterranea

-Guarnaccia, V., & Crous, P. W. (2017). Emerging citrus diseases in Europe caused by species of Diaporthe. IMA fungus, 8(2), 317-334.

**R: Revised as recommended (we removed the reference 11 and added the suggested ones)**

Personally, I agree with this atypical inoculatin way, I mean for the cut twigs. However, it should be better if the authors support this method with other article(s) where the same method has been used on Citrus spp.

**R: Revised as recommended**

Please start the sentence with the entire name Lasiodiplodia. Do not abbreviate the first word.

**R: In this particular line, we did not include the full name since it has already been cited using the entire name when it has been first mentioned in the manuscript.**
